# Contribution of the Twin Arginine Translocation system to the exoproteome of *Pseudomonas aeruginosa*

**DOI:** 10.1038/srep27675

**Published:** 2016-06-09

**Authors:** Geneviève Ball, Haike Antelmann, Paul Roger Claude Imbert, Maxime Rémi Gimenez, Romé Voulhoux, Bérengère Ize

**Affiliations:** 1 Laboratoire d’Ingénierie des Systèmes Macromoléculaires (LISM-UMR7255) CNRS/Aix-Marseille Université, Institut de Microbiologie de la Méditerranée, Marseille, France; 2 Institute for Biology-Microbiology, Freie Universität Berlin, Königin-Luise-Strasse 12-16, D-14195 Berlin, Germany

## Abstract

The opportunistic pathogen *Pseudomonas aeruginosa* uses secretion systems to deliver exoproteins into the environment. These exoproteins contribute to bacterial survival, adaptation, and virulence. The Twin arginine translocation (Tat) export system enables the export of folded proteins into the periplasm, some of which can then be further secreted outside the cell. However, the full range of proteins that are conveyed by Tat is unknown, despite the importance of Tat for the adaptability and full virulence of *P. aeruginosa*. In this work, we explored the *P. aeruginosa* Tat-dependent exoproteome under phosphate starvation by two-dimensional gel analysis. We identified the major secreted proteins and new Tat-dependent exoproteins. These exoproteins were further analyzed by a combination of *in silico* analysis, regulation studies, and protein localization. Altogether we reveal that the absence of the Tat system significantly affects the composition of the exoproteome by impairing protein export and affecting gene expression. Notably we discovered three new Tat exoproteins and one novel type II secretion substrate. Our data also allowed the identification of two new start codons highlighting the importance of protein annotation for subcellular predictions. The new exoproteins that we identify may play a significant role in *P. aeruginosa* pathogenesis, host interaction and niche adaptation.

Protein secretion allows proteins to be transported out of the cell to the surface or into the extracellular milieu. Several secretion pathways fulfill this task in either one (Type I, III, IV and VI) or two steps (Type II, V and IX)^1^,^2^. In the type II secretion pathway, proteins are initially delivered into the periplasm by one of two inner membrane (IM) protein export systems, Sec or Tat (for Twin Arginine Translocation), before being transported through the outer membrane (OM) *via* the General secretion pathway (Xcp in *Pseudomonas aeruginosa*).

Both Sec and Tat-dependent proteins are synthesized as precursors with cleavable N-terminal signal peptides which allow specific targeting to the respective machineries. Signal peptides possess a positively charged N-region, a central hydrophobic H-region, and a polar C-terminal region that contains the signal peptidase cleavage site to allow the liberation of the mature form in the periplasm^3^. Tat signal peptides harbor a conserved S/TRRxFLK consensus motif, where the twin arginine is invariant and in the vast majority of cases essential for efficient export^4^,^5^. Tat signal peptides tend to be longer than Sec signal peptides and the H-region is less hydrophobic with more glycine but less leucine residues^6^. *In silico* methods using these criteria allow the prediction of N-terminal signal peptides (SignalP) and Tat signal peptides (TatP, TatFind and TatPred)^7^,^8^,^9^,^10^,^11^.

While the Sec system exports unfolded substrates, the Tat system transports proteins that require cytoplasmic folding. The Tat pathway is involved in the export of redox proteins that function in energy metabolism but also in numerous other processes such as cellular division, motility, adaptation of bacteria to particular environments, and biofilm formation^12^. Tat substrates have been well characterized in *Escherichia coli* K12 and *Streptomyces* but very few have been experimentally identified in pathogenic Gram-negative bacteria despite the involvement of this pathway in the virulence of numerous plant and human pathogens including *P. aeruginosa*^13^ (for a review see ref. 14). *P. aeruginosa* is a ubiquitous Gram-negative bacterium and an important opportunistic human pathogen. *P. aeruginosa* is responsible for chronic lung infections and mortality in cystic fibrosis patients, for life-threatening infections in immune-compromised humans, and causes 10% of all nosocomial hospital-acquired infections. So far only three extracellular proteins (or exoproteins) have been experimentally demonstrated to be Tat-dependent in *P. aeruginosa*: PlcH and PlcN, two phospholipases C and GlpQ, a phosphodiesterase involved in the hydrolysis of deacylated phospholipids^15^,^16^. All three proteins are produced under phosphate starvation conditions and are secreted by the T2SS/Xcp machinery^16^,^17^,^18^.

Here, we set out to identify the full repertoire of *P. aeruginosa* Tat-dependent proteins secreted into the growth medium (or exoproteome as defined in ref. 19) under nutrient rich and phosphate starvation conditions by two-dimensional (2D) gel analysis. Although no Tat-dependent exoproteins were identified under nutrient rich conditions we found that Tat substrates make a major contribution to the exoproteome during phosphate starvation, a condition that induces the synthesis of virulence factors^20^. We discovered and rigorously verified a number of new Tat substrates. We propose that these Tat-dependent exoproteins as well as other new T2SS exoproteins that we identified are likely to play significant roles in *Pseudomonas* virulence and colonization of the environment.

## Results

### Comparison of *P. aeruginosa* wild type and *tat* mutant exoproteomes

In numerous bacteria, inactivation of the Tat system leads to a defect in cell envelope integrity^21^,^22^,^23^. In *E. coli* this is linked to the export defect of two Tat-dependent cell wall amidases, AmiA and AmiC which are involved in peptidoglycan remodeling^24^,^25^. Consequently, *E. coli tat* mutants show a cell envelope defect that is characterized by leakage of periplasmic proteins, hypersensitivity to drugs and detergents, and a chain-forming phenotype^21^,^25^. Notably, in *Salmonella* Typhimurium, this Tat-dependent envelope defect has been linked to the virulence defect of the *tat* mutant^26^. *P. aeruginosa* PAO1 contains two cell wall amidase homologues, PA5538 (orthologue to *E. coli* AmiC) predicted to contain a Tat signal peptide and PA4947 (orthologue to *E. coli* AmiB) containing a typical Sec signal peptide. In order to exclude the possibility that the reduced virulence of the *P. aeruginosa* tat mutant is a result of similar Tat-dependent envelope defects and to ensure that the study of protein secretion was feasible in the *P. aeruginosa tat* mutant, we investigated whether the *P. aeruginosa tat* mutants of the PAO1 and PA14 wild type (WT) strains also displayed a cell envelope defect. We show that the *P. aeruginosa tat* mutant does not form chains of cells and is not sensitive to SDS (Supplementary Figure S1). These results indicate that the envelope of the *P. aeruginosa tat* mutant does not show the same Tat-dependent defects that have been observed in other bacteria and are associated with attenuated pathogenicity and the leakage of periplasmic proteins. Our results are supported by data recently obtained by Yakhnina *et al* showing that deletion of the cell wall amidase homologue PA5538 in the PAO1 or PA14 strains had no effect on cell growth or morphology^27^.

**Figure 1:**
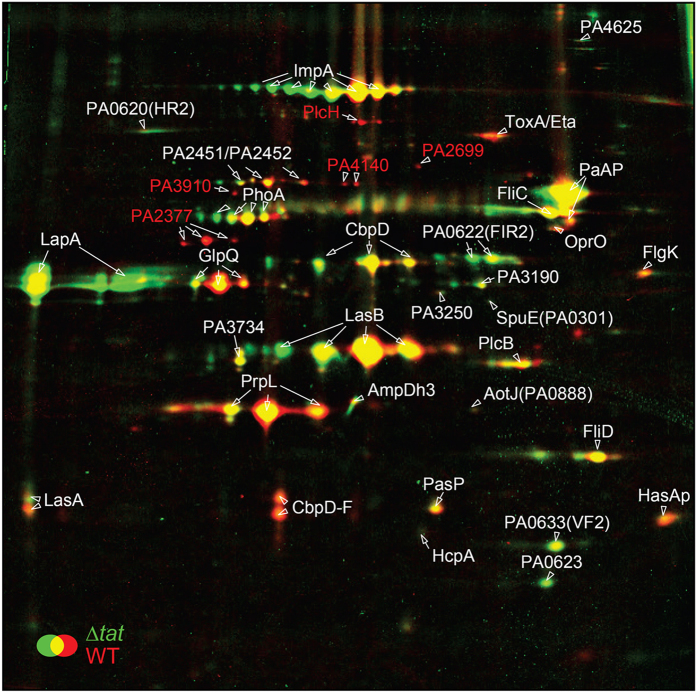
The Tat dependent exoproteome under phosphate starvation conditions. Overlay images of the exoproteome of *P. aeruginosa* PAO1 WT (red) and PAO∆*tat* mutant (∆*tat)* (green) grown in phosphate depleted medium. Exoproteins were separated by 2D-PAGE and quantitative image analysis performed using Decodon Delta 2D. The red spots correspond to proteins that are less abundant in the ∆*tat* mutant and the green spots to proteins that are more abundant in the ∆*tat* mutant. Proteins were identified by MALDI-TOF MS/MS, labelled in the 2D gels and listed in Table 1 and Supplementary Table S1. One of four replicate gels is shown; the other replicates are shown in Supplementary Figure S2.

Next, to identify *P. aeruginosa* Tat-dependent exoproteins, PAO1 WT and *tat* mutants were grown in rich medium or under phosphate starvation and proteins were separated using 2D-gel electrophoresis. We chose phosphate starvation because it is a condition known to influence both the production of virulence factors and known Tat exoproteins^16^,^20^. The extracellular 2D-gel profiles of WT and *tat* strains grown in rich medium did not show any visible differences and were not analyzed further. In contrast, under phosphate starvation conditions the extracellular profiles of WT and *tat* mutant were noticeably different (Fig. 1). In all, a total of 36 different proteins were identified and their levels quantified in both the WT and *tat* mutant (Tables 1, 2 and Supplementary Table S1). The subcellular localizations and the mechanisms of secretion for each of the 36 proteins are listed in Table 1. The exoproteome analysis revealed that secreted proteins represent the major and most abundant part of the exoproteome with only minor contamination by proteins from other bacterial fractions. These include proteins such as flagella-related proteins, that are likely to originate from flagella that are shed from cells in shaking cultures^28^ as well as proteins that are known to be secreted in outer membrane vesicles (OMV)^29^,^30^.

In agreement with our visual examination our quantitative mass spectrometry analysis revealed significant differences in the levels of at least 8 proteins between the WT and *tat* strains (Table 2). Five proteins were present at 3-8-fold lower levels in the *tat* mutant strain and so represented potential Tat-dependent exoproteins (red spots). These proteins were identified as PlcH, a known Tat substrate, the putative alkaline phosphatase PA3910 and the hypothetical proteins PA4140, PA2377 and PA2699 (Fig. 1 and Table 1). Notably, PA3910 is homologous to PhoD, a well-known Tat substrate in *Bacillus subtilis* and *Streptomyces coelicolor*^31^,^32^. In addition we found that the *tat* mutant exoproteome contained 2-fold lower levels of exotoxin A (Eta/ToxA) and the CbpD-fragment (CbpD-F), and 3-fold higher levels of the low molecular weight alkaline phosphatase LapA. We also noted that GlpQ, which we previously identified as a Tat substrate in *P. aeruginosa* PAK strain^16^, could be detected in the *tat* mutant exoproteome although at 1.7-fold lower levels than the WT. Altogether these results reveal for the first time the phosphate-starvation exoproteome of *P. aeruginosa*, and identify the fraction that is dependent on the Tat system for secretion.

### Deletion of the Tat system modifies gene expression

**Figure 2:**
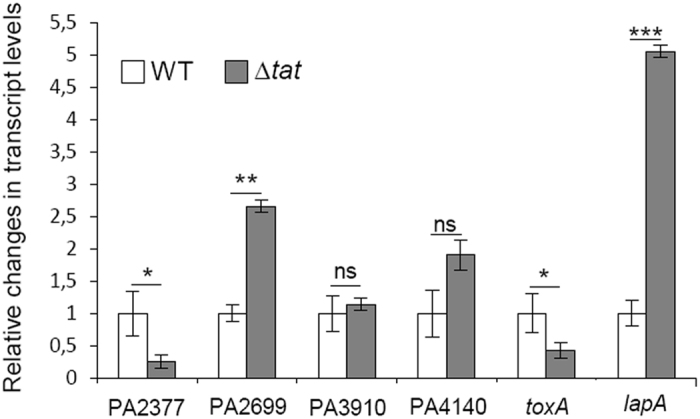
Expression of genes encoding proteins with altered abundance in the Tat exoproteome. qRT-PCR was performed to compare the transcript level of indicated genes in the *P. aeruginosa* PAO1 (WT) and PAOΔ*tat* (Δ*tat*) strains grown in phosphate starvation conditions. Relative fold-changes in transcript levels were assessed after normalization to 16S rRNA. Error bars represent the standard error of the mean of relative fold change in expression from independent experiments (WT n = 3; Δ*tat* n = 4). Two-tailed p values were calculated using Student’s *t*-test (*P < 0.1, **P < 0.01, ***P < 0.001 relative to WT).

The absence of the Tat system unexpectedly leads to a decreased amount of ToxA in the extracellular medium (Fig. 1). ToxA is a well-known T2SS substrate that is believed to be exported through the Sec pathway. Interestingly, in 2002, Ochsner and collaborators showed that the transcription of the *toxA* gene was decreased in a *tat* mutant strain to 25% of the WT^13^. Therefore, in order to differentiate Tat-dependent exoproteins from exoproteins whose genes are misregulated in the *tat* mutant, we performed quantitative RT-PCR (qRT-PCR) (Fig. 2). We detected significantly reduced transcript levels for the PA2377 and *toxA* genes in the *tat* mutant compared to the WT strain. Therefore, the reduced levels of these proteins in the *tat* exoproteome are likely to be caused by reduced transcription and not by impaired Tat-dependent protein export. Notably, PA2699, PA3910 and PA4140 expression levels were either higher or similar in the *tat* mutant suggesting that these genes may encode true Tat substrates.

We also analyzed transcript levels for *lapA* whose gene product is present at 3-fold higher levels in the exoproteome of the *tat* mutant than in the WT (Table 2). We found that *lapA* expression was induced 5 fold in the *tat* mutant, and is therefore likely to explain the higher abundance of this protein in the *tat* mutant exoproteome (Fig. 2).

### GlpQ is not a Tat-dependent exoprotein

**Figure 3:**
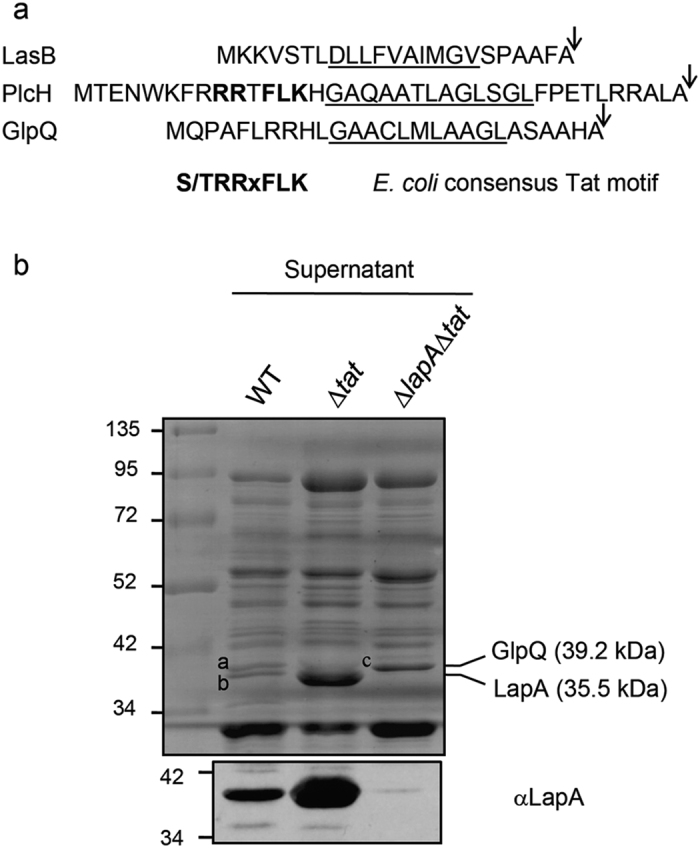
GlpQ is not a Tat substrate. **(a)** Amino acid sequences of LasB (PA3724, protein id AAG07111), PlcH (PA0844, protein id AAG04233) and GlpQ (PA0347, protein id AAG03736) signal peptides. Residues common to the consensus *E. coli* twin arginine motif are shown in bold, the hydrophobic H-regions are underlined and the signal peptidase I cleavage sites are indicated by arrows. **(b)** Supernatant fractions of PAO1 (WT), *tat* mutant (Δ*tat*), and *lapA tat* double mutant (Δ*lapA*Δ*tat*) grown in phosphate depleted medium were separated by SDS-PAGE and stained with Coomassie blue (top panel) or analyzed by immunoblot with a polyclonal anti-LapA antibody (bottom panel). Mass spectrometry identified GlpQ (in bands a and c) and LapA (in b and b). Molecular weight markers (kDa) are indicated on the left of the blot.

The exoproteome analysis revealed that GlpQ was still present in the PAO1 *tat* mutant exoproteome, although at a 1.7-fold decreased level (Fig. 1 and Table 2). This rather weak response was surprising because we previously identified GlpQ as a Tat-dependent exoprotein in *P. aeruginosa* strain PAK^16^. This is probably not due to strain differences because GlpQ from the *P. aeruginosa* PAO1 and PAK strains share 99.47% sequence identity and have the same signal peptide. Interestingly, while the N-terminal signal peptide of GlpQ contains two consecutives arginines, GlpQ is not predicted to be a Tat substrate by Tat prediction programs (Fig. 3a).

In order to re-investigate the Tat-dependence of GlpQ secretion we compared extracellular fractions of WT and *tat* mutant strains (Fig. 3b). Inspection of the gel at the size expected for mature GlpQ (39.2 kDa) showed two closely migrating bands in the WT (annotated a and b) which were identified as GlpQ and LapA by mass spectrometry. In the ∆*tat* mutant however, overproduction of LapA, confirmed by immunoblot analysis, obscured the visibility of GlpQ. Next, analysis of GlpQ secretion in the ∆*lapA*∆*tat* double mutant confirmed that the overproduced protein corresponds to LapA and revealed that GlpQ is still present in the *tat* mutant (annotated c on Fig. 3b). We conclude from this experiment that GlpQ is still secreted in the *tat* mutant and therefore is not a Tat-dependent exoprotein.

### PA2377 is a new Xcp-dependent exoprotein

**Figure 4:**
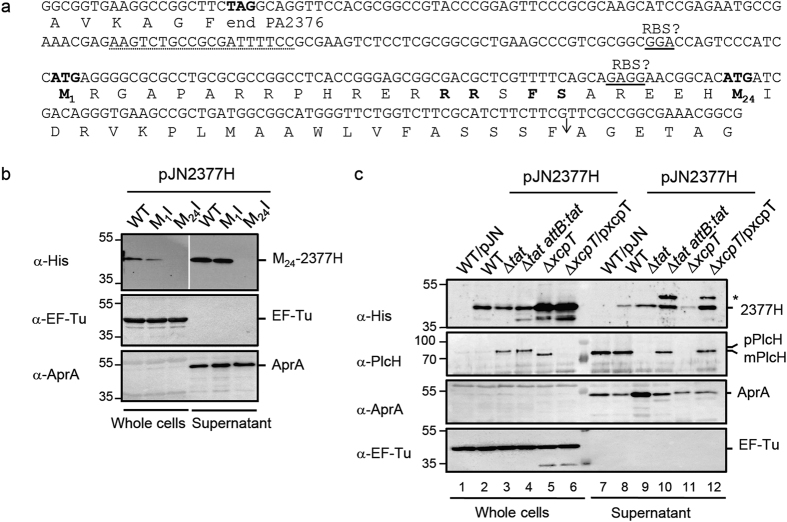
PA2377 is a new Tat-independent and T2SS/Xcp-dependent exoprotein. **(a)** Nucleotide sequence of the *PA2377* promoter region. The C-terminal and N-terminal amino acid sequences of PA2376 and PA2377, respectively, are given below the nucleotide sequence in one-letter code. Putative RBS sequences are underlined. The translation start site annotated on the genome (M_1_) and the alternative translation start site (M_24_) are indicated in bold. The forward primer used to amplify the *PA2377* upstream region is underlined (dotted line). Residues common to the *E. coli* consensus twin arginine motif are shown in bold, the hydrophobic H-region is in italics and the signal peptidase I cleavage site is indicated by an arrow. **(b)** Immunoblot analysis of whole cells and supernatant fractions of PA14 carrying pJN2377H (WT), pJN2377H-M_1_I (M_1_I) or pJN2377H-M_24_I (M_24_I), grown in LB + arabinose (0.4%) medium; the same results were obtained when cells were grown in phosphate limiting medium. The predicted sizes of PA2377H precursor forms are 47.6 kDa from M_1_ and 44.7 kDa from M_24_. The predicted size for PA2377H mature form is 42.4 kDa. **(c)** Immunoblot analysis of whole cells and supernatant fractions of PA14 carrying the empty vector (WT/pJN), and of PA14, PA14Δ*tat*, PA14Δ*tat attB::tat*, PA14Δ*xcpT*, and PA14 Δ*xcpT*/p*xcpT* carrying pJN2377H grown in phosphate depleted medium. PlcH (82.7 kDa for the precursor form, 78.3 kDa for the mature form) is used as a Tat- and Xcp-dependent control while EF-Tu (43.3 kDa) and AprA (50.4 kDa) are used as markers for whole cells and supernatants. Molecular weight markers are indicated on the left of the blot.

Our qRT-PCR analysis showed that PA2377 transcription was decreased in the *tat* mutant giving a possible explanation for its absence from the *tat* exoproteome (Figs 1 and 2). However the N-terminal region of PA2377 shows some of the characteristics of a Tat signal peptide with a long signal peptide and a possible twin arginine motif RRxF albeit not localized next to the H-region (Fig. 4a and ref. 6). Moreover *in silico* analysis of the PA2377 genomic region suggested that there is a second start codon AUG_2_ (encoding M_24_) associated with a ribosomal binding site (RBS) sequence and located 66 bp downstream of the annotated start codon AUG_1_ (encoding M_1_) (Fig. 4a). An algorithm developed by Kolaskar and Reddy for the identification of start codons predicts that AUG_2_ is a good start codon while AUG_1_ is not^33^. If AUG_2_ was used PA2377 would possess a typical Sec-signal peptide. Finally, while PA2377 is annotated as a putative ABC transporter periplasmic iron-binding protein, we clearly identified it in the extracellular medium of the WT strain.

To investigate whether PA2377 synthesis is initiated from M_1_ and/or M_24_ we constructed the plasmid pJN2377H and two variants where each of the putative initiation codons was mutated to AUA causing a methionine-to-isoleucine replacement (pJN2377H-M_1_I and pJN2377H-M_24_I). Since PA2377 transcription was down-regulated in the *tat* mutant (Fig. 2) we put PA2377 and its variants under the control of an arabinose inducible promoter (P_BAD_) and with the addition of a C-terminal hexa-histidine (His_6_) epitope-tag. Cells carrying pJN2377H and pJN2377H-M_1_I but not pJN2377H-M_24_I produced an exoprotein of ~50 kDa corresponding to the expected size of PA2377H (Fig. 4b). This result indicates that PA2377 synthesis is initiated from M_24_ and not from M_1_, and demonstrates that PA2377 is a new exoprotein. The initiation of PA2377 translation from M_24_ would suggest that PA2377 export occurs Tat-independently (Fig. 4a). To determine how PA2377 is secreted out of the cell, we compared the secretion profile of a WT strain, a ∆*tat* mutant, a ∆*xcpT* mutant and the respective complemented strains. As expected, the known Tat- and T2SS/Xcp-dependent exoprotein PlcH was secreted as a mature protein (mPlcH) in the WT strain, but accumulated as an uncleaved precursor (pPlcH) in the ∆*tat* mutant and probably as a periplasmic mature protein in the ∆*xcpT* mutant (Fig. 4c, lanes 7, 8, 3, and 5; and ref. 16). PlcH secretion could be restored in ∆*tat* and ∆*xcpT* mutant strains by *cis*- and *trans*-complementation respectively (Fig. 4c, lanes 10 and 12). Interestingly, PA2377H was secreted in the ∆*tat* strain but accumulated largely intracellularly in the ∆*xcpT* mutant (Fig. 4c lanes 9 and 5). These results indicate that PA2377H export is not Tat-dependent and that it is secreted *via* the T2SS machinery. Interestingly, the PA2377 homologue in *E. carotovora*, ECA2134, is predicted to contain a Sec signal peptide and was also shown to be present in the extracellular medium and secreted by the T2SS/Out machinery adding confidence to our data here^34^. Altogether, we conclude that the reduced levels of PA2377 observed in the *tat* mutant exoproteome are a result of transcriptional downregulation, that PA2377 is neither a Tat substrate nor a periplasmic protein, and that PA2377 is instead a novel T2SS/Xcp dependent exoprotein with a typical Sec signal peptide.

### PA3910 and PA4140 are new Tat/Xcp-dependent exoproteins

**Figure 5:**
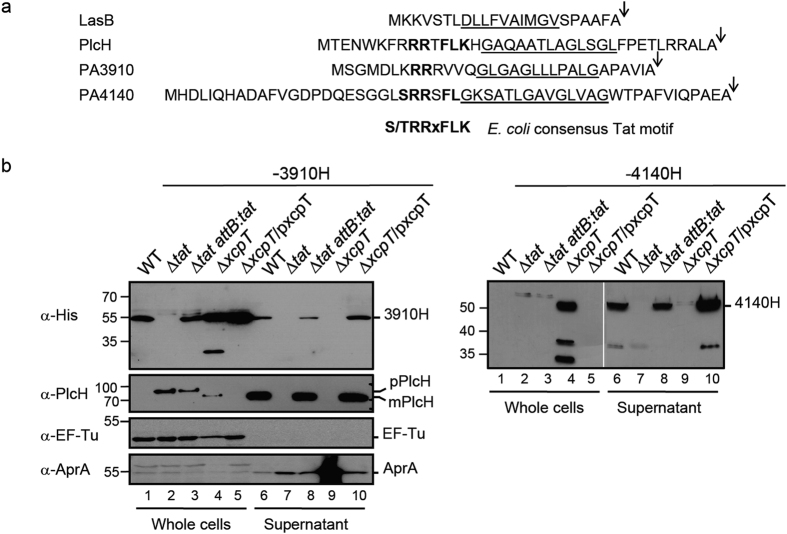
PA3910 and PA4140 are two new Tat/Xcp-dependent exoproteins. **(a)** Amino acid sequences of LasB (PA3724, protein id AAG07111), PlcH (PA0844, protein id AAG04233), PA3910 and PA4140 (protein id AAG07297 and AAG07527 respectively) signal peptides. Residues common to the consensus *E. coli* twin arginine motif are shown in bold, the hydrophobic H-regions are underlined and the signal peptidase I cleavage sites are indicated by arrows. **(b)** Whole cells and supernatant fractions of the PA14 (WT), PA14Δ*tat*, PA14Δ*tat attB*:*tat*, PA14Δ*xcpT*, and PA14Δ*xcpT*/pxcpT grown in phosphate depleted medium and carrying a chromosomal encoded C‐terminal His_6_ epitope-tagged PA3910 (−3910H; 58.7 kDa for the precursor form, 55.6 kDa for the mature form) or PA4140 (−4140H; 65.3 kDa for the precursor form, 60.6 kDa for the mature form). PlcH (82.7 kDa for the precursor form, 78.3 kDa for the mature form), EF-Tu a cytoplasmic protein (43.3 kDa) and AprA a T1SS-dependent exoprotein (50.4 kDa) are used as Tat/Xcp markers, whole cells and supernatant markers.

PA3910 and PA4140 are candidates for new Tat-substrates since they are present at significantly lower levels in the *tat* mutant exoproteome (Fig. 1 and Table 2) and their transcript levels are similar in the WT and the *tat* mutant (Fig. 2). PA3910 and PA4140 are also predicted to contain a Tat signal peptide by the TatFind and TatP prediction programs (Fig. 5a).

To confirm Tat dependence, cellular localization and find out how PA3910 and PA4140 are secreted in the extracellular medium, both genes were replaced by genes encoding His_6_ epitope-tagged proteins (PA4140H and PA3910H) on the chromosome of PA14 WT, ∆*tat* and ∆*xcpT* mutants and their respective complemented strains (Fig. 5b). These constructs allowed us to show that PA3910H and PA4140H are both secreted in a Tat-dependent manner (Fig. 5b lanes 2 and 7) *via* the Xcp/T2SS machinery (Fig. 5b lanes 4 and 9). However, we note that PA3910H secretion is not very efficient since the protein can still be detected in whole cells of the wild type and the *tat* complemented strain. The presence of the mature size protein in whole cells indicates that the protein has been exported through the IM and successfully processed but that subsequent secretion through the OM is limiting. We hypothesize that the presence of the histidine tag on PA3910H may impair periplasmic T2SS recognition. Taken together, these data confirm and support our proteomic data and show that PA3910 and PA4140 are authentic new Tat substrates in *P. aeruginosa* and that both are exoproteins that are secreted into the extracellular medium by the T2SS/Xcp machinery.

### PA2699 is a new Tat-substrate translated from a corrected translation start site

**Figure 6:**
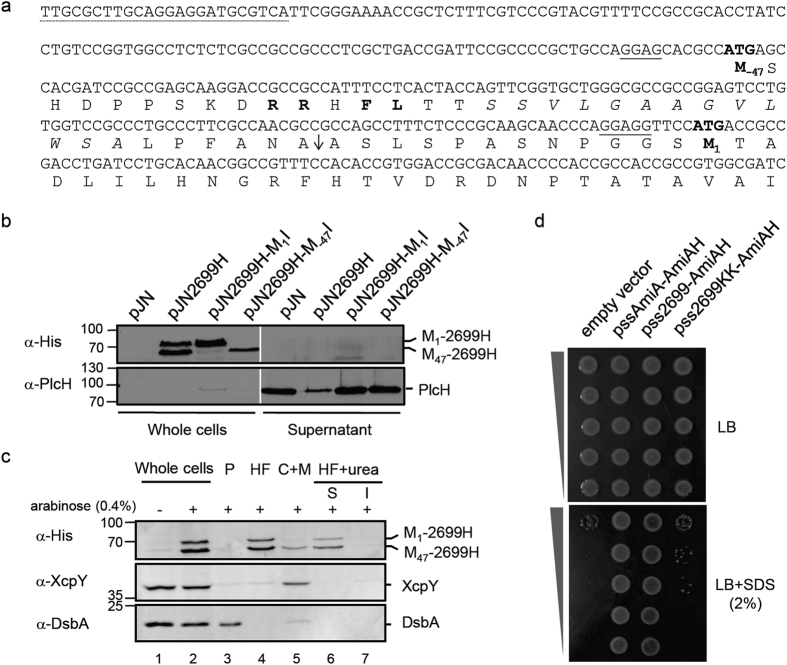
PA2699 is a Tat-dependent exoprotein when translated from an upstream alternative initiation codon. **(a)** Nucleotide sequence of the *PA2699* upstream region. The N-terminal amino acid sequence of PA2699 is given below the nucleotide sequence in one-letter code. Putative RBS sequences are underlined. The translation start site annotated in the genome (M_1_) and the alternative translation start site (M_−47_) are indicated in bold. The forward primer used to PCR amplifies the PA2699 upstream region is underlined (dotted line). Residues common to the *E. coli* consensus twin arginine motif are shown in bold, the hydrophobic H-region is in italics and the signal peptidase I cleavage site is indicated by an arrow. **(b)** Immunoblot analysis of whole cells and supernatant fractions of PA14 carrying the empty vector (pJN), pJN2699H, pJN2699H-M_1_I or pJN2699H-M_−47_I, grown in phosphate depleted medium supplemented with arabinose (0.4%). The predicted sizes of PA2699H forms are 73.5 kDa (from M_−47_) and 68.8 kDa (from M_1_). PlcH (precursor: 82.7 kDa, mature form: 78.3 kDa) is used as supernatant quality control. Molecular weight markers are indicated on the left of the blot. **(c)** Immunoblot analysis of whole cells, periplasmic (P), cytoplasmic and membranes (C + M), and heavy (HF) fractions of PA14 carrying pJN2699H grown in phosphate depleted medium supplemented or not with arabinose (+/−0.4%). The protein disulfide isomerase DsbA (23.3 kDa) and General secretion pathway protein L XcpY (41.3 kDa) are shown as periplasmic and membrane controls respectively. **(d)** SDS viability assay of *E. coli* MCDSSAC carrying pUNI-PROM (empty vector), pssAmiA-AmiAH, pss2699-AmiAH and pss2699KK-AmiAH.

PA2699 was identified as another candidate Tat-substrate since it is absent from the *tat* mutant exoproteome and its transcription is not downregulated in the *tat* mutant (Figs 1 and 2). However, the annotated PA2699 does not contain a Tat signal peptide. We inspected the genomic sequence around the annotated start codon AUG_1_ (encoding M_1_) and noticed that another putative start codon AUG_2_ (encoding M_−47_) and a canonical RBS were located 141 bp upstream of AUG_1_ in the same open reading frame (Fig. 6a). However, neither codon quite meets the threshold value for consideration as an initiation codon using the Kolaskar and Reddy prediction algorithm (both score 24 and a score of ≥26 is considered an initiation codon)^33^. If M_−47_ was used as translation start site PA2699 would then possess a typical Tat signal peptide with a RRHFL motif and a signal peptidase I cleavage site ANA (Fig. 6a).

We first attempted to test whether PA2699 is a new Tat-dependent exoprotein by replacing the PA2699 chromosomal gene with a His_6_ epitope-tagged protein. However, we were unable to detect the protein in phosphate depleted or rich media. Therefore, we next placed PA2699 under control of a P_BAD_ promoter in the plasmid pJN2699H. Remarkably upon induction we could detect two distinct forms consistent with translation initiation from AUG_1_ and AUG_2_ . Independent inactivation of these AUG start codons in pJN2699H-M_1_I and pJN2699H-M_−47_I showed that either AUG could be used as a translation initiation codon (Fig. 6b). Surprisingly, however neither form was localized in supernatant. To determine where both forms of PA2699 were localized in whole cells we further fractionated WT cells carrying pJN2699H. We found that both forms of PA2699H fractionated mainly with the heavy fraction consisting of protein aggregates and could be solubilized with 8 M urea (Fig. 6c). It is therefore likely that the presence of the His_6_ epitope-tag may impair PA2699 conformation and thus correct folding and export.

To further address the Tat-dependence of PA2699, we next used an *E. coli* Tat reporter assay based on the amidase AmiA which allows the identification of Tat signal peptides^35^. When fused to a Tat signal peptide, AmiA is exported into the periplasm and allows the rescue of the SDS sensitive phenotype in an *E. coli* MCDSSAC reporter strain that lacks periplasmic AmiA and AmiC activities. Critically, AmiA does not rescue SDS sensitivity when fused to a Sec signal peptide^25^. When fused to AmiA, the putative Tat signal peptide of PA2699 (from M_−47_ to A_−13_; Fig. 6a) allowed the complementation of the chain-forming and SDS sensitive phenotypes of the *E. coli* reporter strain (Fig. 6d). The fusion protein failed to confer SDS resistance when produced in a *tat* mutant or when the conserved twin arginine was replaced by twin lysine, confirming that ssPA2699-AmiAH is exported in a Tat-dependent manner (Fig. 6d). Altogether these data show that PA2699 contains a cryptic upstream start codon that allows the production of a second isoform of the protein possessing a functional and in frame Tat signal peptide. This is in agreement with the data obtained in our proteomic analysis which indicated that PA2699 is a Tat-dependent exoprotein (Fig. 1).

## Discussion

Our characterization of the *P. aeruginosa* exoproteome has provided the first global map of *P. aeruginosa* proteins that are secreted under phosphate starvation, a condition known to influence virulence factor production^20^. We identified 36 major proteins present in the supernatant of the WT PAO1 strain (Fig. 1 and Table 1). Out of these 36 proteins, 24 were known soluble exoproteins or proteins associated with OMV secretion (Table 1). In addition, we identified new *P. aeruginosa* exoproteins whose localization was previously unknown. These include a probable protein binding component of an ABC sugar transporter PA3190, the putative alkaline phosphatase PA3910 and the hypothetical proteins PA2377, PA2451, PA2452, PA2699, PA3250, PA3734 and PA4140 (Table 1). We further used cell fractionation to verify that three of these (PA2377, PA3910 and PA4140) are indeed new exoproteins secreted by the T2SS (Figs 4, 5, 6).

The *P. aeruginosa* genome has been screened for the presence of Tat signal peptides, and depending on the stringency of the method used, between 18 and 57 candidate substrates have been identified^13^,^36^,^37^. To date, only five of these candidates have been experimentally shown to be Tat substrates: two secreted phospholipases^16^ and three periplasmic proteins involved in pyoverdine biosynthesis and agmatine metabolism^38^,^39^,^40^. However, *in silico* approaches can only provide an estimate of the true number of Tat substrates and cannot predict whether a protein exported into the periplasm will subsequently be secreted through the OM. Therefore, to gain experimental evidence for new secreted Tat substrates, we compared the *P. aeruginosa* WT and *tat* mutant exoproteomes in nutrient- rich and phosphate starvation conditions. Although we did not detect Tat-dependent proteins under nutrient-rich conditions we identified and experimentally verified three new Tat substrates, PA3910, PA4140 and PA2699, produced and secreted in phosphate depleted medium. While PA3910 and PA4140 possess predicted Tat signal peptides, PA2699 does not (Figs 5 and 6). Interestingly, we show that translation of PA2699 can be initiated at two translation start sites M_1_ and M_−47_ (Fig. 6a,b). Whilst PA2699 initiated at M_1_ does not possess a signal peptide, PA2699 initiated at M_−47_ contains a fully functional Tat signal peptide (Fig. 6d). Phylogenetic analysis indicates that PA2699 is conserved in all *Pseudomonas* groups but that the upstream Tat signal peptide has been completely lost in the plant-associated *Pseudomonads*, *P. fluorescens*, *P. syringae* and *P. chlororaphis* (Supplementary Figures S3 and S4). Our identification of PA2699 in the exoproteome of PAO1 and the fact that the PA2699 Tat signal peptide is conserved in some *Pseudomonas* species strongly supports the physiological relevance of the alternative initiation codon AUG_2_ in *P. aeruginosa*. We additionally showed that PA2699 translation is also initiated from AUG_1_ to produce an isoform lacking a Tat signal peptide, albeit when expressed from a plasmid. To our knowledge this could be the first example of a bacterial protein whose two distinct cellular localizations depend on two distinct start codons.

As discussed above we show that the genome annotation for PA2699 missed an authentic Tat peptide. We also show that the initiation codon is miss-assigned for PA2377. The correct initiation codon gives a typical Sec signal peptide rather than the long and unusual signal peptide encoded by the annotated gene (Fig. 4). Together, these results emphasize the limitation of genome annotation for the prediction of subcellular localization and export pathway targeting. We therefore propose that the number of proteins exported through the IM (thus possessing a signal peptide) is probably under estimated in *P. aeruginosa* because of initiation codon missannotation.

We found that the other two new Tat substrates, PA3910 and PA4140, are secreted *via* the general T2SS/Xcp machinery (Fig. 5). Only two other *P. aeruginosa* exoproteins were previously known to be secreted via the Tat-T2SS/Xcp pathway, PlcH and PlcN (Figs 4 and 5 and ref. 16). These data strengthen the mosaic model for T2SS secretion where substrates can be exported into the periplasm by the Sec or Tat machineries before being secreted out of the cell by the T2SS/Xcp secreton. This would also suggest that Tat substrates are not an exception but rather a general feature of the T2SS.

Although the Tat system exports only a few substrates in most organisms, the deletion of *tat* genes is associated with a large array of defects including virulence in plants and human pathogens^13^,^14^. However the molecular mechanisms underlying the loss of virulence in *tat* mutants are generally unknown. One exception comes from a study in *Salmonella* Typhimurium where the virulence defect of the *tat* mutant is shown to be likely indirect and primarily due to envelope defects^26^. Notably, we demonstrate in this work, that the *P. aeruginosa tat* mutants have an intact envelope (Supplementary Figure S1). Surprisingly, an orthologue of the *E. coli* and *S.* Typhimurium AmiC is encoded on the *P. aeruginosa* genome and is most probably a Tat substrate since it possesses a typical Tat signal peptide. This implies that the cell division mechanism is probably different between *E. coli, S.* Typhimurium and *P. aeruginosa* and strongly suggests that envelope defects cannot explain the attenuated virulence of the *P. aeruginosa tat* mutant.

In some organisms, the contribution of the Tat system to virulence is at least partially direct since individual substrates were shown to be involved in the Tat-dependent virulence^22^,^23^. In *P. aeruginosa,* the Tat system allows the export of PlcH, a known virulence determinant in a variety of mammal infection models^41^,^42^. PvdN and PvdP, both involved in the biosynthesis of Pyoverdine, an iron binding siderophore essential for *P. aeruginosa* full virulence, are also Tat substrates^13^,^38^,^43^. Although it has not been directly demonstrated, loss of PlcH secretion and/or Pyoverdine biosynthesis is probably one of the reasons for the attenuation of virulence of the *P. aeruginosa tat* mutant. Here we show that, the deletion of the *tat* genes affects, directly or indirectly, the secretion of multiple exoproteins, all of which are or could be involved in virulence. One of these proteins, ToxA, is reduced (a 2.46-fold decrease) in the *tat* mutant (Fig. 1 and Table 2) due to transcriptional downregulation (Fig. 2 and ref. 13). Since ToxA has a major role in *P. aeruginosa* virulence^44^, its reduced level in the extracellular medium is likely to significantly contribute to the reduced virulence of the *tat* mutant. We also demonstrate that the absence of the Tat system induces a 7.7-fold decrease in the extracellular level of a new exoprotein, PA2377, also because of transcriptional downregulation (Figs 1 and 2 and Table 2). PA2377 is annotated as a hypothetical protein with similarity to the periplasmic iron-binding component of an ABC transporter. However, the absence of neighboring genes encoding an ABC transporter and the fact that we find PA2377 secreted in the external medium casts doubt on the predicted function. As mentioned by Coulthurst and collaborators, the similarity of PA2377/ECA2134 to iron-binding proteins raises the possibility that it may be a novel iron-binding secreted protein involved in host interactions^45^. In agreement with this hypothesis it is interesting to note that PA2377 expression is upregulated when PAO1 cells are exposed to human airway epithelia^46^ or are grown in acute and chronic murine models of infection^47^. Undoubtedly the molecular genetic mechanisms that lead to the differential gene expression of *toxA*, *lapA*, and PA2377 in the *tat* mutant will be interesting to study in greater detail.

We also show that Tat is directly required for the secretion of three new exoproteins (PA2699, PA3910 and PA4140) whose functions have not yet been demonstrated. Interestingly, transcriptome analyses of *P. aeruginosa* in interaction with human airway epithelial cells or grown in acute and chronic murine models of infection indicate that all three of these genes are upregulated during infection^46^,^47^,^48^. Moreover we found that these three proteins were only present in the exoproteome during phosphate starvation, a condition known to stimulate virulence factor production. Taken together, these findings suggest that these proteins might be important for *P. aeruginosa* infection of host cells and survival. PA2699 is predicted to encode a protein with homology to members of the YtcJ-like family (Pfam PF07969, amidohydrolase_3 family) which is part of the metal-dependent amidohydrolase superfamily. The second new Tat-dependent exoprotein, PA3910, is predicted to encode a phosphodiesterase/alkaline phosphatase D. PA3910 has also been named EddA for Extracelullar DNA (eDNA) degradation protein because it is part of a two gene operon with EddB/PA3909, an extracellular DNase involved in eDNA degradation^49^. Interestingly *P. aeruginosa* is able to grow on eDNA as sole source of carbon, nitrogen or phosphate^49^. Use of DNA as a nutrient may provide *P. aeruginosa* with a competitive advantage in environments where eDNA accumulates such as in biofilms or during interactions with other organisms^50^. Because PA3910 is predicted to encode a phosphodiesterase/alkaline phosphatase D and because we show that it is extracellular, we hypothesize that PA3910 acts synergistically with PA3909 to degrade eDNA. Finally, the third new Tat-dependent exoprotein, PA4140, is predicted to encode a hypothetical protein with a substrate binding domain found in cholesterol oxidases (CHOs; Pfam 09129). CHOs are bacterial flavoproteins that catalyse the first step in the degradation of cholesterol^51^. Phylogenetic analysis showed that PA4140 is part of the family of class II CHOs where the FAD redox cofactor is covalently bound to the enzyme through a conserved histidine. This analysis also revealed that all class II CHOs possess a highly conserved twin arginine motif, which suggests that the use of the Tat pathway is a general feature for this family of enzymes (data not shown). In pathogenic bacteria CHOs have been shown to be involved in degradation of eukaryotic membranes and thus might help during the infection process^52^.

In conclusion, we present a global picture of the *P. aeruginosa* exoproteome under phosphate starvation, a condition known to stimulate virulence factor production. We have also identified novel exoproteins whose localization is affected directly or indirectly by the absence of a functional Tat system. These new “Tat-dependent” exoproteins are good virulence factors candidates, and their reduced levels could explain the attenuated virulence of the *P. aeruginosa tat* mutant. Future work will focus on the elucidation of the molecular mechanisms that may link these individual proteins to the attenuation of virulence in the *P. aeruginosa tat* mutant.

## Experimental Procedure

### Bacterial strains, plasmids and growth conditions

Bacterial strains, plasmids and growth conditions used and constructed in this study are described in Supplementary methods and listed in Supplementary Table S2. For clarity, PAO1 PA numbers were systematically used instead of PA14 PA numbers.

### Analysis of RNA by quantitative Real Time PCR

For qRT-PCR, total bacterial RNA was isolated with the SV Total RNA Isolation System (Promega) as described previously^53^. Next, RNAs were precipitated, treated with DnaseI (RTS Dnase kit - Ozyme), and cDNAs were synthesized by reverse transcription using Superscript III reverse transcriptase and random hexamers as described by the manufacturer (Invitrogen). To check for residual contaminating genomic DNA, control reactions without reverse transcriptase were analyzed using the 16S-RNAup/down primers. The cDNAs obtained were stored at −20 °C until use. The expression levels of the different genes were assessed using SoFast EvaGreen Supermix (Bio-Rad) and the CFX96 Real Time System (Bio-Rad). Two microliters of a 1:25 dilution of the cDNA was used in a total volume of 15 μL. The protocol was 98 °C, 2 min followed by 45 cycles of 98 °C, 5 s; 60 °C, 10 s; and 72 °C, 1 s. Data were acquired after each cycle at 72 °C. A melt curve was run at the end of the 45 cycles to test for the presence of a unique PCR reaction product. The primers used for the PCR amplification of cDNA, were designed using the primer3Plus program available at the website (http://www.bioinformatics.nl/cgi-bin/primer3plus/primer3plus.cgi/) and are shown in Supplementary Table S3. The gene transcription levels were normalized in each strain to the 16S ribosomal RNA gene (16SrRNA) and expressed as ratios to the values of the WT strain (set to 1). Samples were assayed in triplicate for each condition.

### Cellular fractionation, SDS-PAGE and immunoblotting

Whole cells and extracellular medium were prepared based on published procedure^54^. Briefly, cells were harvested and extracellular medium collected by centrifugation at 2000 × *g* for 10 min at room temperature. The pellet corresponding to whole cells was washed with Tris 40 mM pH 7.6, resuspended in SDS-PAGE loading buffer containing β-mercaptoethanol and heated at 95 °C for 10 min. Two third of the supernatant corresponding to the extracellular medium was carefully pipetted into a new tube and centrifuged once again for 5 min at 13,000 × *g* at room temperature. Next, extracellular proteins corresponding to two third of the supernatant were concentrated by adding TCA to a final concentration of 10% (vol/vol) and incubating on ice for at least 1h. The proteins were collected by centrifugation at 13,000 × *g* for 30 min at 4 °C, washed twice with ice-cold acetone, and collected again by centrifugation. The pellet corresponding to extracellular proteins was resuspended in SDS-PAGE loading buffer containing β-mercaptoethanol and heated at 95 °C for 10 min.

Fractionation of cells into spheroplast (cytoplasm and membranes) and periplasmic fractions were done as described^54^. Briefly cells corresponding to 5 uDO were pelleted and washed in 1 ml of 50 mM Tris-HCl (pH 7.6) before to be resuspended in 0,5 ml of 50 mM Tris-HCl (pH 7.6) −200 mM MgCl_2_ buffer. The mixture was incubated for 30 min at 30 °C with gentle shaking, left on ice for 5 min and then allowed to stand 15 min at room temperature. These steps were repeated a second time. The spheroplasts were then pelleted at 8,000 × *g* for 10 min at 4 °C and the supernatant was saved as periplasmic fraction. The pellet corresponding to the spheroplasts was resuspended in 1 ml of 50 mM Tris-HCl (pH 7.6), recentrifuged and the pellet, recovered in 1 ml of 50 mM Tris-HCl (pH 7.6), was disrupted by sonication 4 times for 15 s. Unbroken spheroplasts and protein aggregates (heavy fraction, HF) were collected by a low speed centrifugation at 2,000 × *g* for 10 min at 4 °C and the supernatant was saved as cytoplasm and membranes fraction (C + M). Protein aggregates present in the heavy fraction were solubilized in 1 ml of 50 mM Tris-HCl (pH7.6) 100 mM Glycine 8 M urea. The samples were incubated for 60 min on a roller drum at room temperature, and then ultracentrifuged at 120,000 × *g* for 60 min at 4 °C. The supernatant fraction containing the solubilized proteins and the pellet fraction containing insoluble material were retained. Each pellet was resuspended in SDS-PAGE loading buffer containing β-mercaptoethanol and heated at 95 °C for 10 min. Proteins corresponding to the periplasm, to the cytoplasm and periplasm fractions, or to insoluble material were precipitated as described above before to be resuspended in loading buffer containing β-mercaptoethanol and heated at 95 °C for 10 min.

Proteins were analysed by SDS-PAGE and immunoblotting as described in details in Supplementary methods.

### Extracellular proteome analysis and MALDI-TOF-TOF Mass Spectrometry

Extracellular proteome preparation and analysis were performed as described previously^55^. For preparation of the extracellular proteins, the equivalent of 200 OD units of cell culture supernatant was TCA precipitated overnight at 4 °C. The extracellular proteins were collected by centrifugation at 10,000 rpm for 90 min at 4 °C and the resulting protein pellet were washed 5–6 times with 96% ethanol (v/v) before to be dried. Separation of 200 μg extracellular proteins was performed using the immobilized pH gradients in the pH range 3–10. In the second dimension, the equilibrated IEF strips were separated by 12,5% SDS-polyacrylamide gel electrophoresis and visualized with Coomassie brilliant blue. Quantitative image analysis was performed from the Coomassie-stained 2D gels using the DECODON Delta 2D software (http://www.decodon.com). The 2D gel images from wild type and the Δ*tat* mutant exoproteomes were aligned using a warp transformation. Before spot detection and quantification was performed, a fused 2D gel of both images was created using the ‘union fusion’ algorithm of Delta2D. Spot detection was performed in the fusion gel containing all spots present in both images according to the automatically suggested parameters for background subtraction, average spot size, and spot sensitivity. The resulting spot shapes were reviewed and manually edited in the fusion gel if necessary. This reviewed spot mask served as a spot detection consensus for all gel images, which was applied to both images to guide the spot detection and quantification. This enables spot quantification in all gels at the same locations resulting in 100% matching and in a reliable analysis of complete expression profiles. Normalization was performed by calculating the quantity of each single spot in percentage related to the total spot quantity per gel. Experiments were performed and quantified for two independent biological replicates and repeated technically twice.

For identification of the proteins from 2D gels, spot cutting, tryptic digestion of the proteins and spotting of the resulting peptides onto the MALDI-targets (Voyager DE-STR, PerSeptive Biosystems) were performed using the Ettan Spot Handling Workstation (Amersham-Biosciences, Uppsala, Sweden) as described previously^56^. The MALDI-TOF-TOF measurement of spotted peptide solutions was carried out on a Proteome-Analyzer 4800 (Applied Biosystems, Foster City, CA, USA) as described previously^56^. The Mascot search was performed against the available *P. aeruginosa* database (http://www.pseudomonas.com).

### *In silico* analysis/computational methods

The presence and location of N-terminal signal peptide cleavage sites were predicted using the SignalP 3.0 server (http://www.cbs.dtu.dk/services/SignalP-3.0/) and the SignalP-HMM output^7^. Tat signal peptides were predicted using TatFind (http://signalfind.org/tatfind.html), TatP 1.0 (http://www.cbs.dtu.dk/services/TatP/) and TatPred (http://www.compgen.org/tools/PRED-TAT/submit). Identification of RBS was based on the GGAGG core sequence^57^. Identification of the most likely start codon was achieved using the Kolaskar and Reddy method that analyse the −18 to +18 nucleotides around the ATG/GTG^33^.

## Additional Information

**How to cite this article**: Ball, G. *et al.* Contribution of the Twin Arginine Translocation system to the exoproteome of *Pseudomonas aeruginosa. Sci. Rep.* **6**, 27675; doi: 10.1038/srep27675 (2016).

## Supplementary Material

Supplementary Information

## Figures and Tables

**Table 1:** List of proteins present in the exoproteome of the WT and Δ*tat* mutant grown under phosphate starvation and identified by MALDI-TOF MS/MS analysis after tryptic digestion.

PA01 gene number	protein name	function	predicted localization (*computational* or experimental)^a^	Secretion system b
PA0026	PlcB	phospholipase C	Extracellular^58^	
PA0301	SpuE	polyamine transport protein	Periplasmic^59^	
PA0347	GlpQ	glycerophosphoryl diester phosphodiesterase	Extracellular^16^	T2SS^60^
PA0423	PasP	hypothetical protein	Extracellular^61^	
PA0572	ImpA	hypothetical protein	Extracellular^62^	T2SS^62^
PA0620	HR2	R-type pyocin, related to P2 phage; tail fibre	*Extracellular*/OMV^29^	
PA0622	FIR2	R-type pyocin, related to P2 phage; tail sheath	Unknown /OMV^29^	
PA0623		R-type pyocin, related to P2 phage; tail tube	Unknown /OMV^29^	
PA0633	VF2	F-type pyocin, related to l phage; major tail protein	Unknown /OMV^29^	
PA0688	LapA	phosphate ABC transporter, phosphate-binding protein	Extracellular^63^	T2SS (Hxc)^63^
PA0807	AmpDh3	N-acetyl-anhydromuramyl-L-alanine amidase	*Cytoplasm* /OMV^29^	
PA0844	PlcH	hemolytic phospholipase C precursor	Extracellular^17^	T2SS^16^
PA0852	CbpD	chitin-binding protein precursor	Extracellular^64^	T2SS^64^
PA0888	AotJ	arginine/ornithine binding protein	Periplasm^59^/OMV^29^	
PA1086	FlgK	flagellar hook-associated protein 1	*Extracellular*/*Flagelle* /OMV^29^	T3SS, FEA^65^
PA1092	FliC	flagellin type B	*Extracellular*/*Flagelle* /OMV^29^	T3SS, FEA^65^
PA1094	FliD	flagellar capping protein	*Extracellular*/*Flagelle*/OMV^29^	T3SS, FEA^65^
PA1148	ToxA/Eta	Exotoxin A	Extracellular	T2SS^66^
PA1512	HcpA	Secreted protein Hcp	Extracellular^67^	T6SS^67^
PA1871	LasA	LasA protease precursor	Extracellular^66^	T2SS^68^,^69^
PA2377		hypothetical protein	Unknown	
PA2451		hypothetical protein	*Cytoplasm*	
PA2452		hypothetical protein	Unknown	
PA2699		hypothetical protein	Unknown	
PA2939	PaAP	probable aminopeptidase	Extracellular^69^	T2SS^69^
PA3190		probable binding protein component of ABC sugar transporter	*Periplasmic*	
PA3250		hypothetical protein	Unknown	
PA3280	OprO	Pyrophosphate-specific outer membrane porin	OM^70^	
PA3296	PhoA	alkaline phosphatase	Extracellular	T2SS^71^
PA3407	HasAp	heme acquisition protein	Extracellular^72^	T1SS^72^
PA3724	LasB	Elastase	Extracellular^69^	T2SS^69^
PA3734		hypothetical protein	*Cytoplasmic membrane*	
PA3910		Phosphodiesterase/alkaline phosphatase	Unknown	
PA4140		hypothetical protein	Unknown	
PA4175	PrpL	protease IV	Extracellular^73^	
PA4625	CdrA	cyclic diguanylate-regulated TPS partner A, CdrA	Extracellular^74^	T5SS (TPS)^74^

^a^Subcellular localizations were either previously reported or predicted by (i) PSORTb for protein localization sites^75^, (ii) the presence of a putative signal peptide for export across the IM, and (iii) sequence homology to other characterized proteins.

^b^FEA Flagellar export system; T1SS Type I secretion system; T2SS Type II secretion system; T5SS Type V secretion system; T6SS Type VI secretion system; TPS Two partner secretion system.

**Table 2:** Quantification of exoprotein levels in the wild type versus Δ*tat* mutant.

Proteins	1-Ratio WT/Δ*tat*	2-Ratio WT/Δ*tat*	3-Ratio WT/Δ*tat*	4-Ratio WT/Δ*tat*	AV-Ratio WT/Δ*tat*
AmpDh3	0.65	0.61	0.46	0.82	0.63
AotJ(PA0888)	1.34	1.30	0.83	0.61	1.02
CbpD	0.84	1.01	0.80	0.71	0.84
CbpD-F	2.57	2.58	1.77	1.97	2.22
FlgK	1.13	1.29	1.35	1.30	1.27
FliC	0.82	1.00	0.89	0.44	0.79
FliD	0.74	0.80	0.90	0.90	0.83
GlpQ	1.88	1.53	1.53	1.83	1.69
HasAp	1.19	0.93	1.24	1.18	1.13
HcpA	0.97	0.81	1.17	1.11	1.02
ImpA	0.71	0.68	0.78	0.67	0.71
LasA	1.48	n.d	1.09	1.29	1.29
LasB	1.02	1.03	0.94	0.77	0.94
OprO	1.54	1.74	0.88	0.62	1.20
PA0620 (HR2)	0.60	0.40	0.70	0.93	0.66
PA0622 (FIR2)	0.41	0.61	0.56	0.48	0.51
PA0623	0.51	0.68	0.41	0.35	0.49
PA0633 (VF2)	0.60	0.66	0.39	0.38	0.51
PA2377	13.95	6.68	6.94	3.28	7.71
PA2451/PA2452	1.65	1.37	1.24	1.25	1.37
PA2699	6.76	7.04	9.05	4.10	6.74
PA3190	0.62	0.70	0.69	0.61	0.65
PA3250	0.68	0.92	0.58	0.79	0.74
PA3734	0.94	1.06	0.91	1.46	1.10
PA3910	3.52	3.65	3.87	4.94	4.00
PA4140	2.65	4.12	2.18	3.67	3.15
PA4625	0.63	0.73	1.17	0.43	0.74
PaAP	1.33	0.91	0.72	0.48	0.86
PasP	1.29	1.55	1.07	0.94	1.21
PhoA	0.96	0.78	0.79	1.29	0.95
PlcB	1.29	1.47	1.01	0.84	1.15
PlcH	3.05	3.30	1.89	2.87	2.78
PrpL	1.40	1.74	1.41	1.13	1.42
LapA	0.24	0.32	0.51	0.35	0.36
SpuE (PA0301)	0.50	0.64	0.54	0.51	0.55
ToxA/Eta	2.20	2.24	2.05	3.34	2.46

Exoproteins were quantified from dual-channel images of the extracellular proteome of *P. aeruginosa* PAO1 wild type cells (WT) in comparison to the PAOΔ*tat* mutant (Δ*tat*). Proteins in bold are secreted at >2-fold higher levels in the wild type than in the *tat* mutant. These include the confirmed Tat-substrates PlcH, PA3910, PA4140 and PA2699. The level of the LapA protein (in bold and underlined) is higher in the Δ*tat* mutant exoproteome than in the *tat* mutant due to increased transcription. The quantification was performed from 2 biological replicates (labelled 1 and 3) with two technical replicates (labeled 2 and 4) found in Supplementary Figure S2.
