# Supplementary material for: Contribution of the Twin Arginine Translocation system to the exoproteome of Pseudomonas aeruginosa
Source: Sci Rep. 2016 Jun 9;6:27675. doi: 10.1038/srep27675 (PMC4899797; doi:10.1038/srep27675)
Supplement: Supplementary Information [file srep27675-s1.pdf]

SUPPLEMENTARY INFORMATION

Contribution of the Twin arginine Translocation system to the exoproteome of *Pseudomonas aeruginosa*

Geneviève Ball[1], Haike Antelmann[2], Paul Roger Claude Imbert [1#], Maxime Rémi Gimenez [1], Romé Voulhoux[1] and Bérengère Ize[1*]

Laboratoire d'Ingénierie des Systèmes Macromoléculaires (LISM-UMR7255) CNRS/Aix-Marseille Université, Institut de Microbiologie de la Méditerranée, Marseille, France [1]; Institute for Biology-Microbiology, Freie Universität Berlin, Königin-Luise-Strasse 12-16, D-14195 Berlin, Germany [2]

* Address correspondence to Bérengère Ize, berengere.ize@imm.cnrs.fr

# Present address: Cell Biology of Bacterial Pathogenesis (C2BP) BMSSI - CNRS UMR 5086 - Université Lyon 1- Institute of Biology and Chemistry of Proteins, Lyon, France

**Supplementary methods**	p.1
**Supplementary references**	p.6
**Supplementary Legends (Figures and Tables)**	p.10
**Supplementary Figure S1**	p.12
**Supplementary Figure S2**	p.13
**Supplementary Figure S3**	p.15
**Supplementary Figure S4**	p.16
**Supplementary Table S1**	p.17
**Supplementary Table S2**	p.39
**Supplementary Table S3**	p.41

Supplementary methods

Bacterial strains, plasmids and growth conditions

E. coli strains were cultured in Luria-Broth (LB) with antibiotics as required (50 µg ml^{-1} ampicillin (Ap), 25 µg ml^{-1} kanamycin (Kan), 25 µg ml^{-1} tetracycline (Tc), 15 µg ml^{-1} gentamicin (Gm), 30 µg ml^{-1} streptomycin (Sm). The *E. coli* CC118λpir strain was used to propagate pKNG101 (1) derivatives, *E. coli* SM10 to propagate Mini-CTX1 (2) derivatives, while DH5α and TOP10F′ strains were used for other plasmids. Recombinant plasmids were introduced in *P. aeruginosa* using pRK2013 (3) and transconjugants selected on *Pseudomonas* isolation agar (PIA, Difco Laboratories) supplemented with antibiotics as required (500 µg ml^{-1} carbenicillin (Cb), 150 µg ml^{-1} Gm, 2000 µg ml^{-1} Sm, 200 µg ml^{-1} Tc). *P. aeruginosa* strains were cultured in LB with antibiotics as required (150 µg ml^{-1} Cb, 50 µg ml^{-1} Gm, 500 µg ml^{-1} Sm, 50 µg ml^{-1} Tc). For proteomic analysis of *P. aeruginosa* culture supernatant, quantitative RT-PCR or cell fractionation of chromosomal His_6 epitope-tagged strains, cells were grown at 30°C in low inorganic phosphate medium containing 0.4% glucose as described before (4, 5) during stationary phase (18h of growth). For proteomic analysis of *P. aeruginosa* culture supernatant in rich medium, cells were grown in TSB medium (Difco Laboratories) at 37°C. For cell fractionation of *P. aeruginosa* strains carrying pJN105 derivative plasmids, arabinose (between 0.05-0.4%) was added at the beginning of the growth as indicated.

Plasmid and strain construction

All PCR primers employed in this study are listed in Table S3 (Supplementary material), and are based on the PAO1 or PA14 genome sequence (www.pseudomonas.com; 6, 7). All amplifications were carried out with Expand high fidelity PCR system (Roche), using PAO1 or PA14 genomic DNA as template. PCR products were sub-cloned into pCR2.1 and sequenced to verify the absence of any mutation (GATC-biotech).

Unmarked, in-frame deletions and strains chromosomally encoding His_6 epitope-tagged version of the various substrates were constructed by allelic exchange (8). Briefly, N-terminal and C-terminal regions flanking the genes of interest (for deletions) or flanking the stop codon (for His_6 epitope-tag insertion) were PCR amplified using primer pairs indicated in Table S3 and designed for amplifying fragments with overlapping 3′ and 5′ ends. The amplified regions were ligated by overlapping PCR using the most-upstream and -downstream primers in a second PCR with a mix of the two fragments as the matrix. The resulting PCR products were cloned into the pCR2.1 plasmid (TA cloning, Invitrogen) before to be subcloned into the suicide pKNG101 vector (1). The resulting constructs were transformed into *E. coli* CC118λpir and mobilized into PAO1 or PA14 by triparental mating. The strains in which the chromosomal integration event occurred were selected on *Pseudomonas* isolation agar plates containing 2,000 μg of streptomycin per ml. Excision of the plasmid, resulting in the deletion of the chromosomal target gene or insertion of the His_6 epitope-tag, was performed after selection on Luria-Bertani (LB) plates containing 6% sucrose. Clones that became sucrose resistant and streptomycin sensitive were confirmed to contain the gene deletion or tag insertion by PCR analysis.

For the generation of the *tat* cis-complemented strain, PA14Δ*tat attB*::*tat*, the *tatABC* genes along with a 500 bp fragment corresponding to the putative promoter region for the *tatABC* cluster were cloned into the mini-CTX1 vector (2) yielding MiniCTX-*tat*. The cloning was done in two steps. Firstly, the DNA fragment was PCR amplified using Promtat/Endtat and cloned into pCR2.1 (Invitrogen). Secondly, this construct was cut using *Eco*R1 and cloned into the Mini-CTX1 vector. Transfer of this plasmid in *P. aeruginosa* Δ*tat* strain was carried out by triparental mating using *E. coli* SM10 as the donor. The recombinant clones containing the mini-CTX inserted at the *attB* locus on the *P. aeruginosa* genome were selected on tetracycline-containing PIA generating PA14Δ*tat attB::tat*.

pJN2377H (encoding a C-terminal hexa-histidine tag on PA2377) was constructed by PCR amplification of *pa2377* and its 70 bp putative promoter region from *P. aeruginosa* strain PA14 in two steps. First, *pa2377* was amplified using 2377SDup/2377hisdown and cloned into pCR2.1. Six histidine codons followed by the stop codon were included in the reverse primer. Secondly, this construct was cloned into the *Eco*RI site of pJN105 under the transcriptional control of an arabinose-inducible promoter (9). pJN2377H-M_1I and pJN2377H-M_{-47}I were constructed by QuickChange site-directed mutagenesis (Stratagene) using 2377M_1IFor/2377M_1IRev and 2377M_{-24}IFor/2377M_{-24}IRev respectively and pJN2377H as template.

pJN2699H (encoding a C-terminal hexa-histidine tag on PA2699) was constructed as described above for pJN2377H using 2699SDup/2699hisdown. pJN2699H-M_1I and pJN2699H-M_{-47}I were constructed by QuickChange site-directed mutagenesis (Stratagene) using 2699M_1IFor/2699M_1IRev and 2699M_{-47}IFor/2699M_{-47}IRev respectively and pJN2699H as template.

pssAmiA-AmiA-H was constructed by PCR amplification of a DNA fragment corresponding to the native *tat* promoter (cloned EcoRI-BamHI) followed by a fusion between AmiA signal peptide (cloned BamHI-XbaI) and mature AmiA (cloned XbaI-HindIII) using T7/AmiAR as primers and pssAmiA-AmiA as template. The product was digested with EcoRI and HindIII and cloned into similarly digested pT7-5 (10). The plasmid pss2699-AmiA-H was constructed by overlapping PCR. First the DNA encoding the putative Tat signal peptide of PA2699 was amplified with 2699ssFor/2699ssRev using PA14 chromosomal DNA as template. Next, mature amiA (minus signal peptide) was amplified with AmiAEcFor/T7.5Rev2 from pssAmiA-AmiA-H. PA2699 signal peptide and mature AmiA were ligated in a third PCR with 2699ssFor /T7.5Rev2 as primers and a mix of the two fragments as matrix. The resulting PCR product was digested by BamHI-HindIII and cloned into similarly digested pUNI-

PROM (11). pss2699KK-AmiA-H was constructed by QuickChange site-directed mutagenesis (Stratagene) using 2699KKFor/2699KKRev and pss2699-AmiA-H as template.

Microscopy and Outer membrane integrity assay

For phase contrast microscopy, cells were grown to mid-exponential phase at 37 °C in LB medium and mounted on microscope slides covered with poly-l-lysine (Sigma-Aldrich) to immobilized cells. Fixed cells were imaged with a Zeiss AxioImager m2 equipped with an Hamamatsu OrcaR2 camera with a 100× phase-contrast objective. The images were acquired with AxioVision software. For viability measurements, overnight cultures were adjusted to an OD_{600} of 1, serial dilutions from 10^{-1} to 10^{-6} were prepared in LB, and 5 µL of each dilution were spotted onto LB agar or LB agar + 2% (w/v) SDS. Plates were incubated overnight at 37°C and photographed.

SDS-PAGE and immunoblotting

Proteins were separated by SDS-PAGE and visualized by staining with Coomassie blue or by immunoblotting. Protein samples derived from equivalent amounts of culture (*i.e.* optical density equivalents) were loaded in each lane and compared. Approximately equal protein loading was confirmed by total protein staining of the gel or the nitrocellulose membrane. After electrophoresis, the proteins were transferred to a nitrocellulose membrane for 1 h at 200 mA according to the method of Towbin *et al*. (12). Immunoblotting was performed as described (13) using primary polyclonal antibodies directed against LapA (laboratory stock; dilution 1:10000), AprA (laboratory stock, dilution 1:5000), XcpY (14, dilution 1:2000), DsbA (kindly gifted by K.E. Jaeger – university of Heinrich-Heine, dilution 1:25000) and PlcH (kindly gifted by M.L. Vasil – University of Colorado, dilution 1:500), or monoclonal antibodies directed against EF-Tu (Hycult-biotech, dilution 1:20000) and His_6 epitope-tag (Penta His, Qiagen, dilution 1:1000). For anti His_6 detection, manufacturer instructions were followed. Peroxidase-conjugated anti-Mouse or anti-Rabbit IgGs (Sigma, dilution 1:5000)

were used as secondary antibodies. The membranes were developed with homemade enhanced chemiluminescence and exposed to X-ray film for the appropriate time or were scanned using ImageQuant TL analysis software (GE Healthcare Life sciences).

Supplementary references

1. Kaniga K., Delor I., Cornelis G. R. A wide-host-range suicide vector for improving reverse genetics in gram-negative bacteria: inactivation of the *blaA* gene of *Yersinia enterocolitica*. *Gene.* **109,** 137-41 (1991).

2. Hoang T. T., Kutchma A. J., Becher A., Schweizer H. P. Integration-proficient plasmids for *Pseudomonas aeruginosa*: site-specific integration and use for engineering of reporter and expression strains. *Plasmid.* **43,** 59-72 (2000).

3. Figurski D. H., Helinski D. R. Replication of an origin-containing derivative of plasmid RK2 dependent on a plasmid function provided in trans. *Proc Natl Acad Sci U S A.* **76,** 1648-52 (1979).

4. Cheng K. J., Ingram J. M., Costerton J. W. Alkaline phosphatase localization and spheroplast formation of *Pseudomonas aeruginosa*. *Can J Microbiol.* **16,** 1319-24 (1970).

5. Faure L. M., Llamas M. A., Bastiaansen K. C., de Bentzmann S., Bigot S. Phosphate starvation relayed by PhoB activates the expression of the *Pseudomonas aeruginosa* σvreI ECF factor and its target genes. *Microbiology*.**159**, 1315-27 (2013).

6. Stover C.K. et al. Complete genome sequence of *Pseudomonas aeruginosa* PAO1, an opportunistic pathogen. *Nature*. **406,** 959-64 (2000).

7. Winsor G. L. et al. *Pseudomonas* Genome Database: improved comparative analysis and population genomics capability for *Pseudomonas* genomes. *Nucleic Acids Res.* **39,** D596-600 (2011).

8. Schweizer H. P., Hoang T. T. An improved system for gene replacement and xylE fusion analysis in *Pseudomonas aeruginosa*. *Gene.* **158,** 15-22 (1995).

9. Newman J. R., Fuqua C. Broad-host-range expression vectors that carry the L-arabinose-inducible *Escherichia coli* araBAD promoter and the araC regulator. *Gene*. **227,** 197-203 (1999).

10. Tabor S., Richardson C. C. A bacteriophage T7 RNA polymerase/promoter system for controlled exclusive expression of specific genes. *Proc Natl Acad Sci U S A.* **82,** 1074-8 (1985).

11. Jack R. L. et al. Coordinating assembly and export of complex bacterial proteins. *EMBO J.* **23**: 3962-72 (2004).

12. Towbin H., Staehelin T., Gordon J. Electrophoretic transfer of proteins from polyacrylamide gels to nitrocellulose sheets: procedure and some applications. *Proc Natl Acad Sci U S A.* **76,** 4350-4 (1979).

13. Viarre V. et al. HxcQ liposecretin is self-piloted to the outer membrane by its N-terminal lipid anchor. *J Biol Chem.* **284,** 33815-23 (2009).

14. Michel G, Bleves S, Ball G, Lazdunski A, Filloux A. Mutual stabilization of the XcpZ and XcpY components of the secretory apparatus in *Pseudomonas aeruginosa*. *Microbiology.* **144,** 3379-86 (1998).

15. Dereeper A. et al. Phylogeny.fr: robust phylogenetic analysis for the non-specialist. *Nucleic Acids Res.* **36,** W465-9 (2008).

16. Dereeper A. et al. BLAST-EXPLORER helps you building datasets for phylogenetic analysis. *BMC Evol Biol.* **10,** 8 (2010).

17. Kaniga K., Delor I., Cornelis G. R. A wide-host-range suicide vector for improving reverse genetics in gram-negative bacteria: inactivation of the *blaA* gene of *Yersinia enterocolitica*. *Gene*. **109,** 137-41 (1991).

18. Casadaban M. J., Cohen S. N**.** Lactose genes fused to exogenous promoters in one step using a Mu-lac bacteriophage: in vivo probe for transcriptional control sequences. *Proc Natl Acad Sci U S A.* **76,** 4530-3 (1979).

19. Bogsch E. G. et al. An essential component of a novel bacterial protein export system with homologues in plastids and mitochondria. *J Biol Chem*. **273,** 18003-6 (1998).

20. Ize B., Stanley N. R., Buchanan G., Palmer T. Role of the *Escherichia coli* Tat pathway in outer membrane integrity. *Mol Microbiol*. **48,** 1183-93 (2003).

21. Voulhoux R., Filloux A., Schalk I. J. Pyoverdine-mediated iron uptake in *Pseudomonas aeruginosa*: the Tat system is required for PvdN but not for FpvA transport. *J Bacteriol*. **188,** 3317-23 (2006).

22. Hoang T. T., Kutchma A. J., Becher A., Schweizer H. P. Integration-proficient plasmids for *Pseudomonas aeruginosa*: site-specific integration and use for engineering of reporter and expression strains. *Plasmid*. **43,** 59-72 (2000).

23. Fürste J. P. et al. Molecular cloning of the plasmid RP4 primase region in a multi-host-range tacP expression vector. *Gene* **48,** 119-31 (1986).

24. Hoang T. T., Karkhoff-Schweizer R. R., Kutchma A. J., Schweizer H. P. A broad-host-range Flp-FRT recombination system for site-specific excision of chromosomally-located DNA sequences: application for isolation of unmarked *Pseudomonas aeruginosa* mutants. *Gene*. **212,** 77-86 (1998).

25. Figurski D. H., Helinski D. R. Replication of an origin-containing derivative of plasmid RK2 dependent on a plasmid function provided in trans. *Proc Natl Acad Sci U S A.* **76,** 1648-52 (1979).

26. Ball G., Durand E., Lazdunski A., Filloux A. A novel type II secretion system in *Pseudomonas aeruginosa*. *Mol Microbiol*. **43**, 475-85 (2002).

27. Tabor S., Richardson C. C. A bacteriophage T7 RNA polymerase/promoter system for controlled exclusive expression of specific genes. *Proc Natl Acad Sci U S A.* **82,** 1074-8 (1985).

28. Durand E. et al. The assembly mode of the pseudopilus: a hallmark to distinguish a novel secretion system subtype. *J Biol Chem.* **286,** 24407-16 (2011).

29. Newman J. R., Fuqua C. Broad-host-range expression vectors that carry the L-arabinose-inducible *Escherichia coli araBAD* promoter and the *araC* regulator. *Gene*. **227,** 197-203 (1999).

30. Jack R. L. et al. Coordinating assembly and export of complex bacterial proteins. *EMBO J*. **23,** 3962-72 (2004).

Supplementary Legends

Figure S1. Inactivation of the Tat pathway in *P. aeruginosa* has no effect on cell envelope integrity. (a) Microscopic analysis and **(b)** SDS sensitivity assay of *P. aeruginosa* WT strains (PAO1 and PA14) and their *tat* derivatives (PAOΔ*tat* and PA14Δ*tat*). *E. coli* strains MC4100 (parental strain) and B1LK0 (Δ*tat*C) are used as controls. Scale bars, 5 μm. Note that the presence of SDS in the solid media causes colonies to spread in comparison with LB-only.

Figure S2. Overlay images of the extracellular proteome of *P. aeruginosa* wild type (red image) in comparison to the Δ*tat* mutant (green image) of two biological replicates (labelled 1 and 3) and two technical replicates (labelled 2 and 4).

Figure S3. Phylogenetic relationship of different *Pseudomonas* strains encoding PA2699 and the reference *E. coli* MG1655 strain based on RecA. *Pseudomonas* strains where PA2699 possesses a Tat signal peptide are shown in black, and strains where PA2699 lacks a signal peptide are in violet. The phylogenetic tree was constructed from a multiple amino acid sequence alignment of the RecA protein using MAFFT (Multiple Alignment using Fast Fourier Transform http://mafft.cbrc.jp/alignment/server/) Multiple Sequence Alignment program and phylogeny was inferred using the PhyML maximum likelihood method on the phylogeny.fr platform (15,16). Support values for the tree are indicated above each branch. The tree is drawn to scale, with branch lengths in the same units as those of the evolutionary distances used to infer the phylogenetic tree. The scale for branch length (0.1 substitutions/site) is shown below the tree.

Figure S4. Multiple sequence alignment of the first 50 amino-acid residues of PA2699 proteins containing a signal peptide from different *Pseudomonas* strains. The alignment was generated with the MAFFT Multiple Sequence Alignment program. Residues color coding is from Clustal W: red, small aliphatic, hydrophobic, and aromatics -Y; blue, acidic; magenta, basic - H; green, hydroxyl, sulphydryl, amine, and glycine. Symbols for residues

conservation are from Clustal W: Asteriks indicate conserved residues, two dots residues with high similarity, and one dot residues with low similarity.

Table S1. Identification of exoproteins in the secretome of the WT and Δ*tat* mutant using MALDI-TOF MS/MS

Table S2. Strains and plasmids used in this study.

Table S3. Primer sequences used in this study.

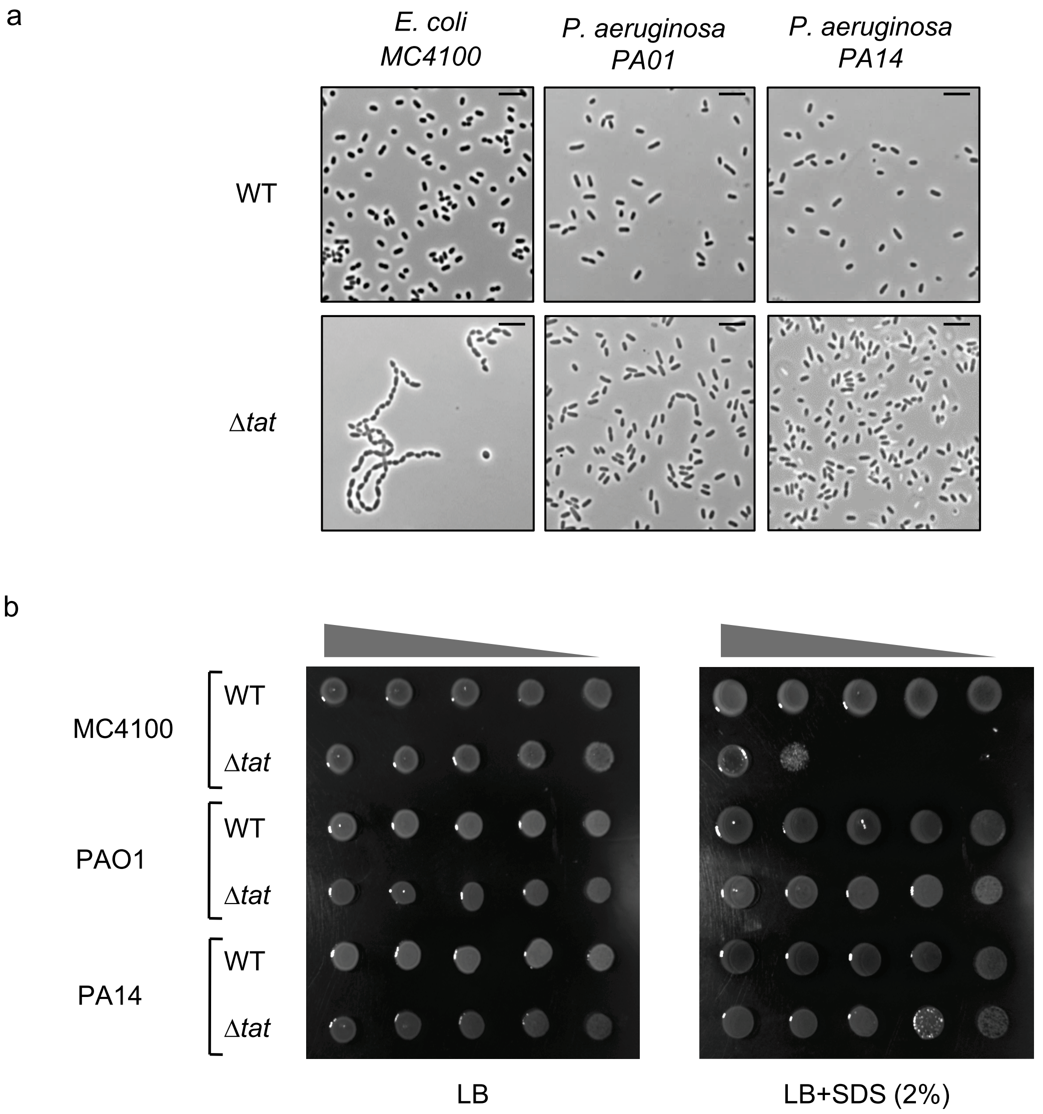
a
E. coli
MC4100
P. aeruginosa
PA01
P. aeruginosa
PA14
WT
Δtat
b
MC4100
WT
Δtat
PAO1
WT
Δtat
PA14
WT
Δtat
LB
LB+SDS (2%)

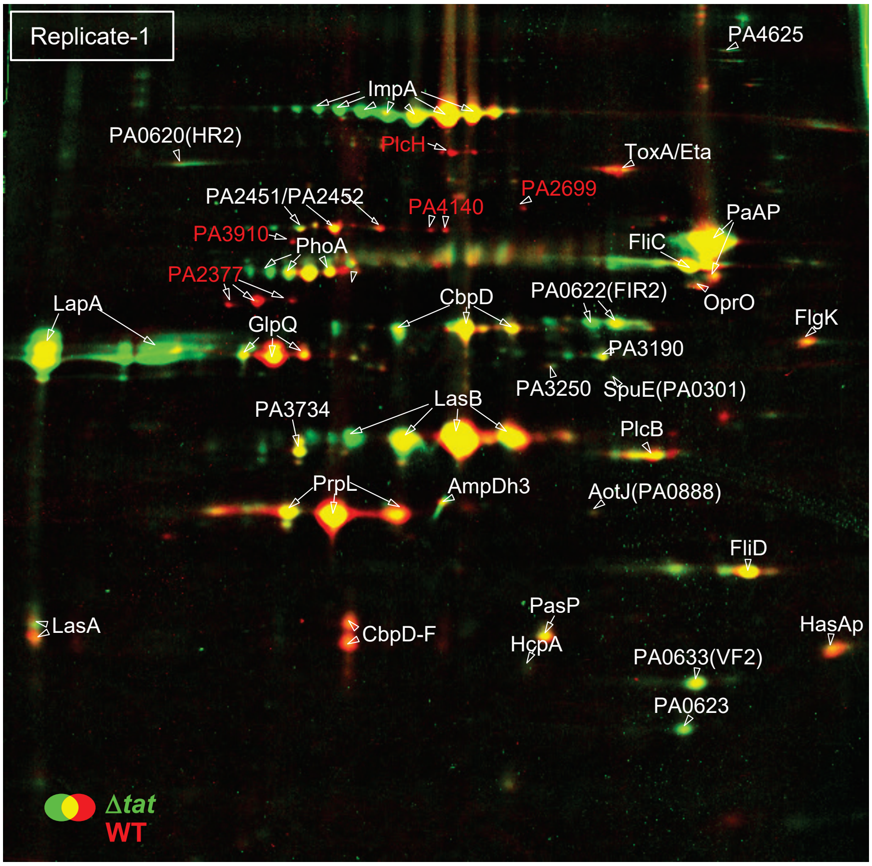

Replicate-1
PA4625
ImpA
PA0620(HR2)
PlcH
ToxA/Eta
PA2451/PA2452
PA4140
PA2699
PA3910
PhoA
PaAP
FliC
PA2377
LapA
CbpD
PA0622(FIR2)
OprO
FlgK
GlpQ
PA3190
PA3250
SpuE(PA0301)
PA3734
LasB
PlcB
PrpL
AmpDh3
AotJ(PA0888)
FliD
PasP
LasA
CbpD-F
HcpA
HasAp
PA0633(VF2)
PA0623
Δ*tat*
WT

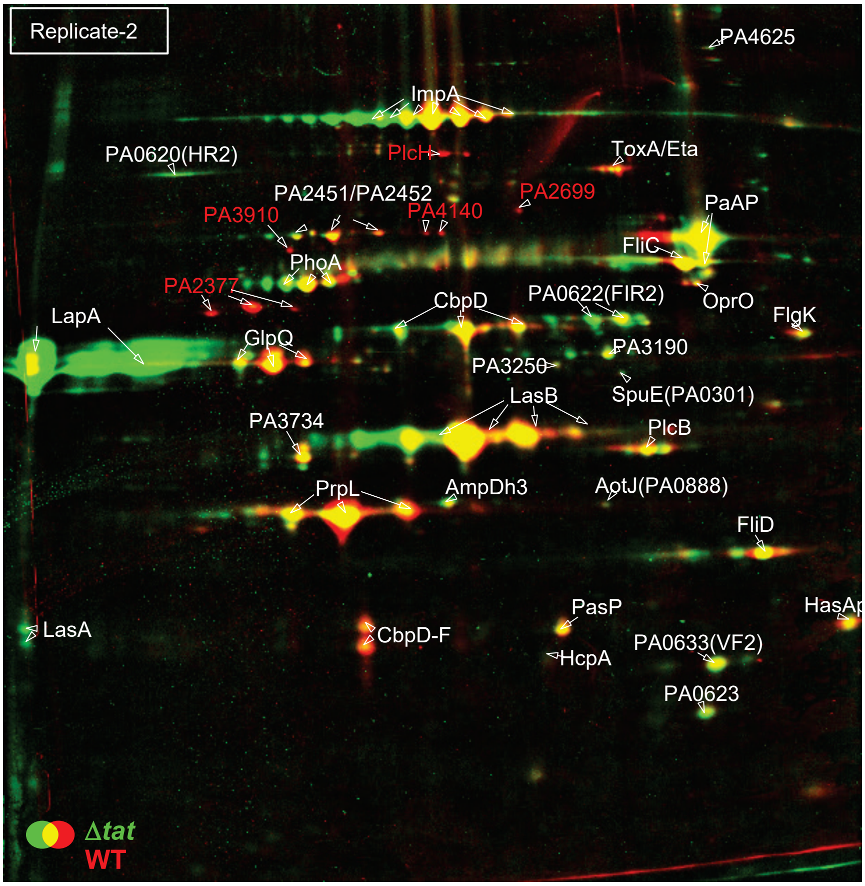

Replicate-2
PA4625
ImpA
PA0620(HR2)
PlcH
ToxA/Eta
PA2451/PA2452
PA2699
PA3910
PA4140
PaAP
FliC
PhoA
PA2377
CbpD
PA0622(FIR2)
OprO
LapA
FlgK
GlpQ
PA3190
PA3250
SpuE(PA0301)
LasB
PA3734
PlcB
PrpL
AmpDh3
AotJ(PA0888)
FliD
PasP
HasAp
LasA
CbpD-F
PA0633(VF2)
HcpA
PA0623
Δ*tat*
WT

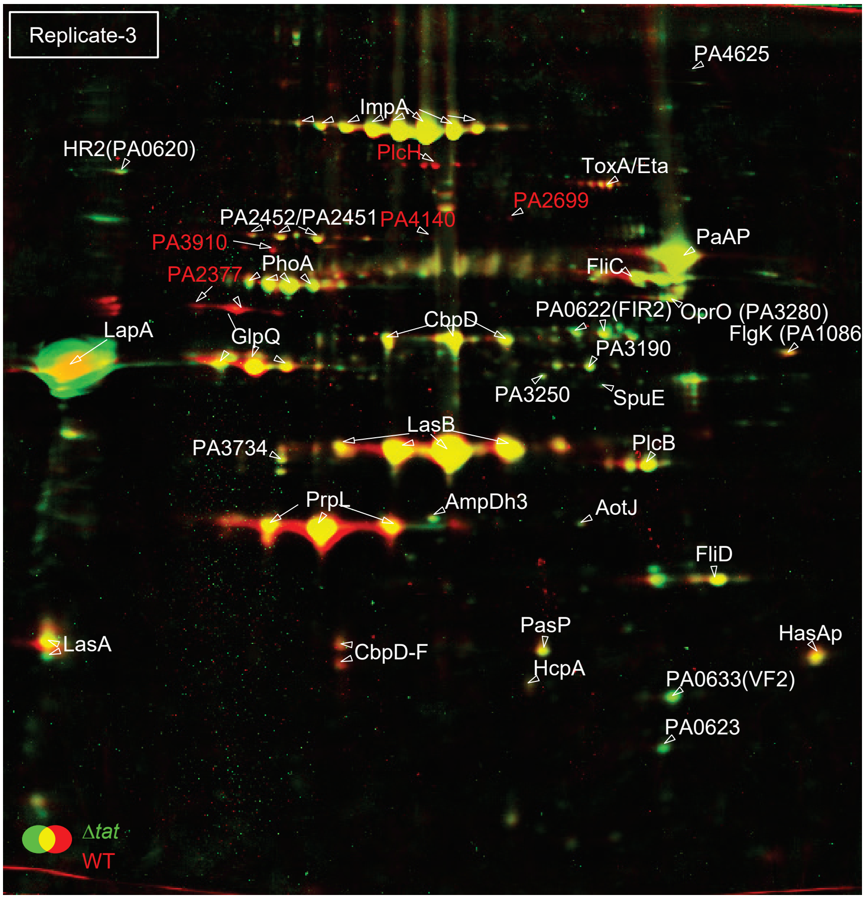
Replicate-3
PA4625
ImpA
HR2(PA0620)
PlcH
ToxA/Eta
PA2699
PA2452/PA2451
PA4140
PA3910
PaAP
PhoA
PA2377
FliC
PA0622(FIR2)
OprO (PA3280)
LapA
GlpQ
CbpD
FlgK (PA1086)
PA3190
PA3250
SpuE
LasB
PA3734
PlcB
PrpL
AmpDh3
AotJ
FliD
PasP
HasAp
LasA
CbpD-F
HcpA
PA0633(VF2)
PA0623
Δtat
WT

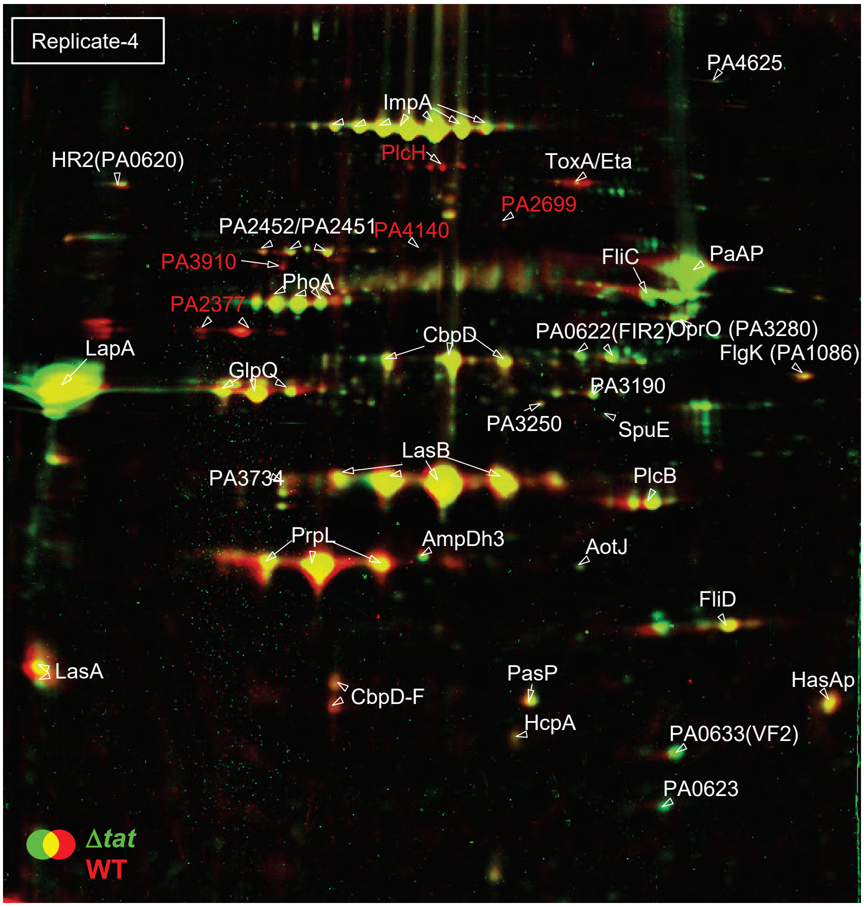
Replicate-4
PA4625
ImpA
HR2(PA0620)
PlcH
ToxA/Eta
PA2699
PA2452/PA2451
PA4140
PA3910
FliC
PaAP
PhoA
PA2377
PA0622(FIR2)
OprO (PA3280)
CbpD
LapA
FlgK (PA1086)
GlpQ
PA3190
PA3250
SpuE
LasB
PA3734
PlcB
PrpL
AmpDh3
AotJ
FliD
LasA
PasP
HasAp
CbpD-F
HcpA
PA0633(VF2)
PA0623
Δtat
WT

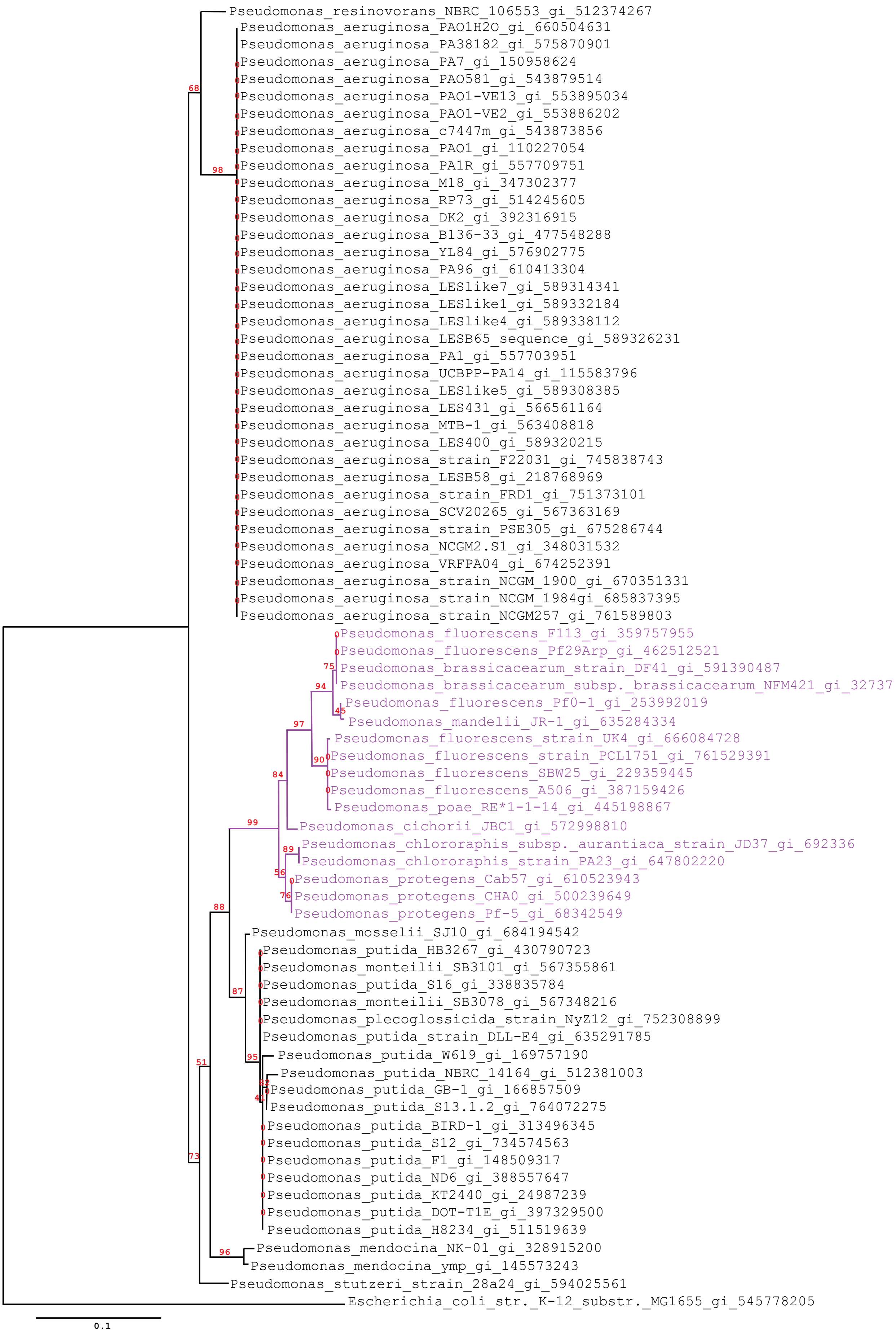
Pseudomonas_resinovorans_NBRC_106553_gi_512374267
Pseudomonas_aeruginosa_PAO1H2O_gi_660504631
Pseudomonas_aeruginosa_PA38182_gi_575870901
Pseudomonas_aeruginosa_PA7_gi_150958624
Pseudomonas_aeruginosa_PAO581_gi_543879514
Pseudomonas_aeruginosa_PAO1-VE13_gi_553895034
Pseudomonas_aeruginosa_PAO1-VE2_gi_553886202
Pseudomonas_aeruginosa_c7447m_gi_543873856
Pseudomonas_aeruginosa_PAO1_gi_110227054
Pseudomonas_aeruginosa_PA1R_gi_557709751
Pseudomonas_aeruginosa_M18_gi_347302377
Pseudomonas_aeruginosa_RP73_gi_514245605
Pseudomonas_aeruginosa_DK2_gi_392316915
Pseudomonas_aeruginosa_B136-33_gi_477548288
Pseudomonas_aeruginosa_YL84_gi_576902775
Pseudomonas_aeruginosa_PA96_gi_610413304
Pseudomonas_aeruginosa_LESlike7_gi_589314341
Pseudomonas_aeruginosa_LESlike1_gi_589332184
Pseudomonas_aeruginosa_LESlike4_gi_589338112
Pseudomonas_aeruginosa_LESB65_sequence_gi_589326231
Pseudomonas_aeruginosa_PA1_gi_557703951
Pseudomonas_aeruginosa_UCBPP-PA14_gi_115583796
Pseudomonas_aeruginosa_LESlike5_gi_589308385
Pseudomonas_aeruginosa_LES431_gi_566561164
Pseudomonas_aeruginosa_MTB-1_gi_563408818
Pseudomonas_aeruginosa_LES400_gi_589320215
Pseudomonas_aeruginosa_strain_F22031_gi_745838743
Pseudomonas_aeruginosa_LESB58_gi_218768969
Pseudomonas_aeruginosa_strain_FRD1_gi_751373101
Pseudomonas_aeruginosa_SCV20265_gi_567363169
Pseudomonas_aeruginosa_strain_PSE305_gi_675286744
Pseudomonas_aeruginosa_NCGM2.S1_gi_348031532
Pseudomonas_aeruginosa_VRFPA04_gi_674252391
Pseudomonas_aeruginosa_strain_NCGM_1900_gi_670351331
Pseudomonas_aeruginosa_strain_NCGM_1984gi_685837395
Pseudomonas_aeruginosa_strain_NCGM257_gi_761589803
Pseudomonas_fluorescens_F113_gi_359757955
Pseudomonas_fluorescens_Pf29Arp_gi_462512521
Pseudomonas_brassicacearum_strain_DF41_gi_591390487
Pseudomonas_brassicacearum_subsp._brassicacearum_NFM421_gi_32737
Pseudomonas_fluorescens_Pf0-1_gi_253992019
Pseudomonas_mandelii_JR-1_gi_635284334
Pseudomonas_fluorescens_strain_UK4_gi_666084728
Pseudomonas_fluorescens_strain_PCL1751_gi_761529391
Pseudomonas_fluorescens_SBW25_gi_229359445
Pseudomonas_fluorescens_A506_gi_387159426
Pseudomonas_poae_RE*1-1-14_gi_445198867
Pseudomonas_cichorii_JBC1_gi_572998810
Pseudomonas_chlororaphis_subsp._aurantiaca_strain_JD37_gi_692336
Pseudomonas_chlororaphis_strain_PA23_gi_647802220
Pseudomonas_protegens_Cab57_gi_610523943
Pseudomonas_protegens_CHA0_gi_500239649
Pseudomonas_protegens_Pf-5_gi_68342549
Pseudomonas_mosselii_SJ10_gi_684194542
Pseudomonas_putida_HB3267_gi_430790723
Pseudomonas_monteilii_SB3101_gi_567355861
Pseudomonas_putida_S16_gi_338835784
Pseudomonas_monteilii_SB3078_gi_567348216
Pseudomonas_plecoglossicida_strain_NyZ12_gi_752308899
Pseudomonas_putida_strain_DLL-E4_gi_635291785
Pseudomonas_putida_W619_gi_169757190
Pseudomonas_putida_NBRC_14164_gi_512381003
Pseudomonas_putida_GB-1_gi_166857509
Pseudomonas_putida_S13.1.2_gi_764072275
Pseudomonas_putida_BIRD-1_gi_313496345
Pseudomonas_putida_S12_gi_734574563
Pseudomonas_putida_F1_gi_148509317
Pseudomonas_putida_ND6_gi_388557647
Pseudomonas_putida_KT2440_gi_24987239
Pseudomonas_putida_DOT-T1E_gi_397329500
Pseudomonas_putida_H8234_gi_511519639
Pseudomonas_mendocina_NK-01_gi_328915200
Pseudomonas_mendocina_ymp_gi_145573243
Pseudomonas_stutzeri_strain_28a24_gi_594025561
Escherichia_coli_str._K-12_substr._MG1655_gi_545778205
0.1

```
                                        10        20        30        40        50
                               ....|....|....|....|....|....|....|....|....|....|
P.aeruginosa_UCBPP_PA14        MSHDPPSKDRRHFLTTSSVLGAAGVLWSALPFANAASLSPASNPGGSMTA

P.aeruginosa_LESlike4          MSHDPPSKDRRHFLTTSSVLGAAGVLWSALPFANAASLSPASNPGGSMTA
P.aeruginosa_LESlike1          MSHDPPSKDRRHFLTTSSVLGAAGVLWSALPFANAASLSPASNPGGSMTA
P.aeruginosa_LESB65            MSHDPPSKDRRHFLTTSSVLGAAGVLWSALPFANAASLSPASNPGGSMTA
P.aeruginosa_LES400            MSHDPPSKDRRHFLTTSSVLGAAGVLWSALPFANAASLSPASNPGGSMTA
P.aeruginosa_LESlike7          MSHDPPSKDRRHFLTTSSVLGAAGVLWSALPFANAASLSPASNPGGSMTA
P.aeruginosa_LESlike5          MSHDPPSKDRRHFLTTSSVLGAAGVLWSALPFANAASLSPASNPGGSMTA
P.aeruginosa_SCV20265          MSHDPPSKDRRHFLTTSSVLGAAGVLWSALPFANAASLSPASNPGGSMTA
P.aeruginosa_LES431            MSHDPPSKDRRHFLTTSSVLGAAGVLWSALPFANAASLSPASNPGGSMTA
P.aeruginosa_MTB-1             MSHDPPSKDRRHFLTTSSVLGAAGVLWSALPFANAASLSPASNPGGSMTA
P.aeruginosa_LESB58            MSHDPPSKDRRHFLTTSSVLGAAGVLWSALPFANAASLSPASNPGGSMTA
P.aeruginosa_strain_F22031     MSHDPPSKDRRHFLTTSSVLGAAGVLWSALPFANAASLSPASNPGGSMTA
P.aeruginosa_strain_NCGM_1984  MSHDPPSKDRRHFLTTSSVLGAAGVLWSALPFANAASLSPASNPGGSMTA
P.aeruginosa_strain_NCGM_1900  MSHDPPSKDRRHFLTTSSVLGAAGVLWSALPFANAASLSPASNPGGSMTA
P.aeruginosa_PAO1H2O           MSHDPPSKDRRHFLTTSSVLGAAGVLWSALPFANAASLSPASNPGGSMTA
P.aeruginosa_PA96              MSHDPPSKDRRHFLTTSSVLGAAGVLWSALPFANAASLSPASNPGGSMTA
P.aeruginosa_YL84              MSHDPPSKDRRHFLTTSSVLGAAGVLWSALPFANAASLSPASNPGGSMTA
P.aeruginosa_PA1R              MSHDPPSKDRRHFLTTSSVLGAAGVLWSALPFANAASLSPASNPGGSMTA
P.aeruginosa_PA1               MSHDPPSKDRRHFLTTSSVLGAAGVLWSALPFANAASLSPASNPGGSMTA
P.aeruginosa_RP73              MSHDPPSKDRRHFLTTSSVLGAAGVLWSALPFANAASLSPASNPGGSMTA
P.aeruginosa_DK2               MSHDPPSKDRRHFLTTSSVLGAAGVLWSALPFANAASLSPASNPGGSMTA
P.aeruginosa_NCGM2.S1          MSHDPPSKDRRHFLTTSSVLGAAGVLWSALPFANAASLSPASNPGGSMTA
P.aeruginosa_strain_PSE305     MSHDPPSKDRRHFLTTSSVLGAAGVLWSALPFANAASLSPSSNPGGSMTA
P.aeruginosa_VRFPA04           MSHDPPSKDRRHFLTTSSVLGAAGVLWSALPFANAASLSPASNPGGSMTA
P.aeruginosa_B136-33           MSHDPPSKDRRHFLTTSSVLGAAGVLWSALPFANAASLSPASNPGGSMTA
P.aeruginosa_strain_FRD1       MSHDPPSKDRRHFLTTSSVLGAAGVLWSALPFANAASLSPASNPGGSMTA
P.aeruginosa_PA38182           MSHDPPSKDRRHFLTTSSVLGAAGVLWSALPFANAASLSPASNPGGSMTA
P.aeruginosa_PAO1-VE13         MSHDPPSKDRRHFLTTSSVLGAAGVLWSALPFANAASLSPASNPGGSMTA
P.aeruginosa_PAO1-VE2          MSHDPPSKDRRHFLTTSSVLGAAGVLWSALPFANAASLSPASNPGGSMTA
P.aeruginosa_PAO581            MSHDPPSKDRRHFLTTSSVLGAAGVLWSALPFANAASLSPASNPGGSMTA
P.aeruginosa_PAO1              MSHDPPSKDRRHFLTTSSVLGAAGVLWSALPFANAASLSPASNPGGSMTA
P.aeruginosa_c7447m            MSHDPPRKDRRHFLTTSSVLGAAGVLWSALPFANAASLSPASNPGGSMTA
P.aeruginosa_M18               MSHDPPSKDRRHFLTTSSVLGAAGVLWSALPFANAASLSPASNPGGSMTA
P.aeruginosa_DNA_strain_NCGM25 MSHDPPSKDRRHFLTTSSVLGAAGVLWSALPFANAASLSPASNPGGSMTA
P.aeruginosa_PA7               MSHDPPSKNRRHFLATSSVLGAAGALWSALPFADAASLSPASNPGGSMTA
P.resinovorans_NBRC_106553     MSEDPSSNSRRQFLAASSVLGAAGALWSALPFAGSTGSAHASTQGGSMTA
P.mosselii_SJ10                MSQDPTDQSRRKFLATSTVLGAAGALWSALPFI   DSAHAALSGDPMSA
P.monteilii_SB3101             MSQDRNDKTRRQFLATSTVLGAAGALWSALPFTGSAGSAHASTQGGSMTA
P.monteilii_SB3078             MSQDRNDKTRRQFLATSTVLGAAGALWSALPFTGSAGSAHASTQGGSMTA
P.putida_S16                   MSQDRNDKTRRQFLATSTVLGAAGALWSALPFTGSAGSAHASTQGGSMTA
P.plecoglossicida_strain_NyZ12 MSQDRNDKTRRQFLATSTVLGAAGALWSALPFTGSAGSAHASTQGGSMTA
P.putida_strain_DLL-E4         MSQDRNDKTRRQFLATSTVLGAAGALWSALPFTGSAGSAHASTQGGSMTA
P.putida_HB3267                MSQDRNDKTRRQFLATSTVLGAAGALWSALPFTGSAGSAHASTQGGSMTA
P.putida_W619                  MSQDPNDKTRRQFLATSTVLGAAGALWSALPFTGAAGSAHASIQGGSMTA
P.putida_GB-1                  MSQDRNDKTRRQFLATSTVLGAAGALWSALPFTGQAGSAHASSQGGSMSA
P.putida_S12                   MSQDRNDKTRRQFLATSTVLGAAGALWSALPFTGHAGSAHASTQGGSMTA
P.putida_BIRD-1                MSQDRNDKTRRQFLATSTVLGAAGALWSALPFTGHAGSAHASTQGGSMTA
P.putida_DOT-T1E               MSQDRNDKTRRQFLATSTVLGAAGALWSALPFTGQAGSAHASTQGGSMTA
P.putida_ND6                   MSQDRNDKTRRQFLATSTVLGAAGALWSALPFTGQAGSAHASSQGGSMTA
P.putida_F1                    MSQDRNDKTRRQFLATSTVLGAAGALWSALPFTGQAGSAHASSQGGSMTA
P.putida_KT2440                MSQDRNDKTRRQFLATSTVLGAAGALWSALPFTGQAGSAHASTQGGSMTA
P.putida_NBRC_14164            MSQDRNDKTRRQFLATSTVLGAAGALWSALPFAGQAGSA ASIQGGSMTA
P.putida_H8234                 MSQDRNDKTRRQFLATSTVLGAAGALWSALPFAGQAGSAHASIQGGSMTA
P.putida_S13.1.2               MSQDRNDKTRRQFLATSTVLGAAGALWSALPFTGQAGSAHASTQGGSMTA
P.stutzeri_strain_28a24        MPQPPFDPDRRRFIATGSLLGAAGALWSSLPFAGTAAGTTHFAAGAHMNA
P.mendocina_ymp                MSQDPSDPKRRHFLATSSVLGAAGALWSTLPFAAMAGDSSASSSGGNMSA
P.mendocina_NK-01              MSRNPHDENRRRILAAGGVLGAAGAIWSALPFATFASDSSTRSPGGSMSA
                               * .      **:::::. :*****.:**:***      :     *  *.*
```

Table S1: Identification of exoproteins in the secretome of the WT and Δ*tat* mutant using MALDI-TOF MS/MS

Rank	Protein Name	Accession No.	Protein Score	Protein Score C. I. %	Total Ion Score	Total Ion C. I. %
1	secreted protein HcpA (PA1512)	gi|15596709|ref|NP_250203.1|	516	100	345	100

Peptide Information

Calc. Mass	Obsrv. Mass	± da	± ppm	Start Seq.	End Seq.	Sequence	Ion Score	C. I. %	Modification	Rank	Result Type
993.5152	993.5339	0.0187	19	94	100	VEIQWYR					Mascot
1020.6564	1020.6675	0.0111	11	64	72	VHKPVVITK					Mascot
1170.5498	1170.5662	0.0164	14	53	63	DPQSGQPTGQR					Mascot
1240.6243	1240.6345	0.0102	8	2	13	ATPAYMSITGTK					Mascot
1256.6191	1256.6357	0.0166	13	2	13	ATPAYMSITGTK			Oxidation (M)[6]		Mascot
1335.7419	1335.7582	0.0163	12	91	100	LTKVEIQWYR	102	100			Mascot
1335.7419	1335.7582	0.0163	12	91	100	LTKVEIQWYR					Mascot
1398.7952	1398.8143	0.0191	14	77	90	ASPLLLAALTSGER	98	100			Mascot
1398.7952	1398.8143	0.0191	14	77	90	ASPLLLAALTSGER					Mascot
1509.9152	1509.9221	0.0069	5	64	76	VHKPVVITKVFDK					Mascot
1741.0219	1741.037	0.0151	9	77	93	ASPLLLAALTSGERLTK					Mascot
1888.0538	1888.0641	0.0103	5	73	90	VFDKASPLLLAALTSGER	145	100			Mascot
1888.0538	1888.0641	0.0103	5	73	90	VFDKASPLLLAALTSGER					Mascot
1933.8676	1933.8779	0.0103	5	151	167	ITWTHEVSGTSGSDDWR					Mascot
2061.9624	2061.9695	0.0071	3	150	167	KITWTHEVSGTSGSDDWR					Mascot
2172.1885	2172.1926	0.0041	2	53	72	DPQSGQPTGQRVHKPVVITK					Mascot
2345.0793	2345.0754	-0.0039	-2	151	172	ITWTHEVSGTSGSDDWRSPVAG					Mascot
2638.3247	2638.3005	-0.0242	-9	101	124	TSAAGTQEHYYTTVLEDAIIVDIK					Mascot

Rank	Protein Name	Accession No.	Protein Score	Protein Score C. I. %	Total Ion Score	Total Ion C. I. %
1	phospholipase C, PlcB, PA0026	gi|15595224|ref|NP_248716.1|	294	100	149	100

Peptide Information

Calc. Mass	Obsrv. Mass	± da	± ppm	Start Seq.	End Seq.	Sequence	Ion Score	C. I. %	Modification	Rank	Result Type
1309.7223	1309.7197	-0.0026	-2	251	263	LNDPAAVANLVGR					Mascot
1383.5812	1383.5811	-0.0001	0	159	169	EDFDTTEAHYR					Mascot
1539.6823	1539.6821	-0.0002	0	158	169	REDFDTTEAHYR					Mascot
1646.6578	1646.6595	0.0017	1	118	132	DGDSHGNPHGGYDYR	88	100			Mascot
1646.6578	1646.6595	0.0017	1	118	132	DGDSHGNPHGGYDYR					Mascot
1835.7938	1835.796	0.0022	1	34	49	DALAFMNSSYATEEMR					Mascot
1851.7888	1851.7889	0.0001	0	34	49	DALAFMNSSYATEEMR			Oxidation (M)[6]		Mascot
1867.7837	1867.7899	0.0062	3	34	49	DALAFMNSSYATEEMR			Oxidation (M)[6,15]		Mascot
1899.9923	1899.9993	0.007	4	251	268	LNDPAAVANLVGRYDPSK					Mascot
2025.2206	2025.214	-0.0066	-3	299	317	VAKELIPESIALTVTVLTK					Mascot
2074.875	2074.8738	-0.0012	-1	118	135	DGDSHGNPHGGYDYRYHK					Mascot
2220.043	2220.0471	0.0041	2	136	154	VDGGIADVDWYAMVYLYNR					Mascot
2236.0378	2236.0413	0.0035	2	136	154	VDGGIADVDWYAMVYLYNR			Oxidation (M)[13]		Mascot
2290.0842	2290.0977	0.0135	6	30	49	NIVKDALAFMNSSYATEEMR					Mascot
2306.0791	2306.0872	0.0081	4	30	49	NIVKDALAFMNSSYATEEMR			Oxidation (M)[10]		Mascot
2322.074	2322.0815	0.0075	3	30	49	NIVKDALAFMNSSYATEEMR			Oxidation (M)[10,19]		Mascot

Calc. Mass	Obsrv. Mass	± da	± ppm	Start Seq.	End Seq.	Sequence	Ion Score	C. I. %	Modification	Rank	Result Type
2354.054	2354.0964	0.0424	18	34	53	DALAFMNSSYATEEMRQAYR					Mascot
2370.0488	2370.062	0.0132	6	34	53	DALAFMNSSYATEEMRQAYR			Oxidation (M)[6]		Mascot
2386.0437	2386.0422	-0.0015	-1	34	53	DALAFMNSSYATEEMRQAYR			Oxidation (M)[6,15]		Mascot
2648.2603	2648.2717	0.0114	4	133	154	YHKVDGGIADVDWYAMVYLYNR					Mascot
2664.2551	2664.259	0.0039	1	133	154	YHKVDGGIADVDWYAMVYLYNR			Oxidation (M)[16]		Mascot
2887.4109	2887.4309	0.02	7	272	296	DLLTQTGQVAYARPEPLYDTSYETR					Mascot
3178.4963	3178.5203	0.024	8	54	83	FYVSAAGSEAQAGEILGQAAFDVDDFKDTR	61	100			Mascot
3178.4963	3178.5203	0.024	8	54	83	FYVSAAGSEAQAGEILGQAAFDVDDFKDTR					Mascot
3243.6279	3243.6475	0.0196	6	269	296	SIRDLLTQTGQVAYARPEPLYDTSYETR					Mascot

Rank	Protein Name	Accession No.	Protein Score	Protein Score C. I. %	Total Ion Score	Total Ion C. I. %
1	polyamine transport protein (SpuE), PA0301	gi|15595498|ref|NP_248992.1|	384	100	209	100

Peptide Information

Calc. Mass	Obsrv. Mass	± da	± ppm	Start Seq.	End Seq.	Sequence	Ion Score	C. I. %	Modification	Rank	Result Type
990.5366	990.5294	-0.0072	-7	89	97	QIQAGAFQK					Mascot
1145.6565	1145.6465	-0.01	-9	256	265	GIDIQYVIPK					Mascot
1346.7427	1346.7369	-0.0058	-4	89	100	QIQAGAFQKLDK					Mascot
1428.8937	1428.8931	-0.0006	0	340	352	LYVSAVLPAKVLR					Mascot
1469.7535	1469.755	0.0015	1	212	223	VRPYVSYFHSSK	84	100			Mascot
1469.7535	1469.755	0.0015	1	212	223	VRPYVSYFHSSK					Mascot
1730.9323	1730.9325	0.0002	0	250	265	AEEAGKGIDIQYVIPK					Mascot
1862.9019	1862.905	0.0031	2	323	339	SVSDSEEVYPPQAVLDK					Mascot
1877.9102	1877.9119	0.0017	1	266	282	EGANLWFDLMAIPADAK			Oxidation (M)[10]		Mascot
2103.0869	2103.0889	0.002	1	69	88	LVSGHSGYDIVVPSNNFLGK					Mascot
2137.0747	2137.0791	0.0044	2	303	322	VSDYVGYANAIPGARPLMDK					Mascot
2153.0696	2153.072	0.0024	1	303	322	VSDYVGYANAIPGARPLMDK	27	98.245	Oxidation (M)[18]		Mascot
2153.0696	2153.072	0.0024	1	303	322	VSDYVGYANAIPGARPLMDK			Oxidation (M)[18]		Mascot
2203.9878	2203.9944	0.0066	3	49	68	ESGIDVSYDVFDSNETLEGK					Mascot
2253.1914	2253.1897	-0.0017	-1	283	302	AADNAYAFIDYLLRPEVIAK	99	100			Mascot
2253.1914	2253.1897	-0.0017	-1	283	302	AADNAYAFIDYLLRPEVIAK					Mascot
2527.2869	2527.2864	-0.0005	0	28	48	SLHIYNWTDYIAPTTLKDFTK					Mascot
2779.3145	2779.2993	-0.0152	-5	224	249	YISDLANGNICVAFGYSGDVFQAAAR			Carbamidomethyl (C)[11]		Mascot
2904.5239	2904.5173	-0.0066	-2	323	349	SVSDSEEVYPPQAVLDKLYVSAVLPAK					Mascot

Rank	Protein Name	Accession No.	Protein Score	Protein Score C. I. %	Total Ion Score	Total Ion C. I. %
1	glycerophosphoryl diester phosphodiesterase GlpQ PA0347	gi|15595544|ref|NP_249038.1|	427	100	176	100

Peptide Information

Calc. Mass	Obsrv. Mass	± da	± ppm	Start Seq.	End Seq.	Sequence	Ion Score	C. I. %	Modification	Rank	Result Type
919.4785	919.4807	0.0022	2	336	342	VHPYTFR					Mascot
959.5269	959.5228	-0.0041	-4	194	201	SLQISQQR					Mascot
1094.6429	1094.6373	-0.0056	-5	166	175	IPTIRPGNAR					Mascot
1142.5549	1142.5537	-0.0012	-1	314	324	DANGNLGQPTR					Mascot
1226.7289	1226.7275	-0.0014	-1	218	228	LGLAMERPLVK					Mascot
1242.7239	1242.7169	-0.007	-6	218	228	LGLAMERPLVK			Oxidation (M)[5]		Mascot
1280.627	1280.6245	-0.0025	-2	343	353	AENSFLPAEFR					Mascot
1563.9078	1563.901	-0.0068	-4	162	175	AIERIPTIRPGNAR					Mascot

1605.8635	1605.8558	-0.0077	-5	240	253	APVFIQSFEVNNLK					Mascot
1684.8693	1684.8646	-0.0047	-3	299	313	YAYGVGPDKSYVIPR					Mascot
1742.8416	1742.8323	-0.0093	-5	354	370	SADGNPQSRGDLAGEIR					Mascot
1897.0079	1897.0026	-0.0053	-3	202	217	TIGLYPEIKHGTHFQR					Mascot
1976.0851	1976.076	-0.0091	-5	240	256	APVFIQSFEVNNLKELK					Mascot
1986.0344	1986.0251	-0.0093	-5	325	342	FVGDAHAAGLKVHPYTFR	57	100			Mascot
1986.0344	1986.0251	-0.0093	-5	325	342	FVGDAHAAGLKVHPYTFR					Mascot
2044.1212	2044.1111	-0.0101	-5	176	193	LDGTFEIPTLQEIIDLVK					Mascot
2122.0452	2122.0447	-0.0005	0	371	390	AYLDAGIDGLFSDQPDVAVR					Mascot
2181.0876	2181.0774	-0.0102	-5	336	353	VHPYTFRAENSFLPAEFR					Mascot
2193.032	2193.0569	0.0249	11	343	362	AENSFLPAEFRSADGNPQSR					Mascot
2209.1108	2209.1033	-0.0075	-3	314	335	DANGNLGQPTRFVGDAHAAGLK					Mascot
2265.053	2265.0454	-0.0076	-3	114	133	HDNELGLTTDVAQHPEFADR	120	100			Mascot
2363.2505	2363.2478	-0.0027	-1	233	253	NGYLGPRAPVFIQSFEVNNLK					Mascot
2393.1479	2393.1497	0.0018	1	114	134	HDNELGLTTDVAQHPEFADRK					Mascot
2704.3438	2704.3308	-0.013	-5	110	133	LVARHDNELGLTTDVAQHPEFADR					Mascot
3018.4512	3018.4954	0.0442	15	263	291	LVQLYGSGQPYDQQAAGGSLTYAEMATAK					Mascot
3034.4463	3034.4473	0.001	0	263	291	LVQLYGSGQPYDQQAAGGSLTYAEMATAK			Oxidation (M)[25]		Mascot
3484.6584	3484.6555	-0.0029	-1	74	106	GASGYVPEHTLGAYALAVMMGADYVEPDLVMTR					Mascot
3500.6533	3500.6743	0.021	6	74	106	GASGYVPEHTLGAYALAVMMGADYVEPDLVMTR			Oxidation (M)[19]		Mascot
3516.6482	3516.6716	0.0234	7	74	106	GASGYVPEHTLGAYALAVMMGADYVEPDLVMTR			Oxidation (M)[19,20]		Mascot
3532.6433	3532.6697	0.0264	7	74	106	GASGYVPEHTLGAYALAVMMGADYVEPDLVMTR			Oxidation (M)[19,20,31]		Mascot

Rank	Protein Name	Accession No.	Protein Score	Protein Score C. I. %	Total Ion Score	Total Ion C. I. %
1	hypothetical protein PA0423 (PasP)	gi|15595620|ref|NP_249114.1|	241	100	77	100

Peptide Information

Calc. Mass	Obsrv. Mass	± da	± ppm	Start Seq.	End Seq.	Sequence	Ion Score	C. I. %	Modification	Rank	Result Type
1233.6011	1233.6022	0.0011	1	31	40	EGQHAFIEFR					Mascot
1251.627	1251.6267	-0.0003	0	43	52	HLGYSWLYGR					Mascot
1433.6808	1433.6807	-0.0001	0	141	153	LIGQGDDPWGGYR					Mascot
1474.7802	1474.787	0.0068	5	31	42	EGQHAFIEFRIK					Mascot
1492.8059	1492.8058	-0.0001	0	41	52	IKHLGYSWLYGR					Mascot
1589.807	1589.8035	-0.0035	-2	28	40	IDKEGQHAFIEFR					Mascot
1632.8129	1632.8159	0.003	2	139	153	AKLIGQGDDPWGGYR					Mascot
1670.8093	1670.8073	-0.002	-1	74	88	VTINTNSVDTNHAER					Mascot
1913.9312	1913.9276	-0.0036	-2	74	90	VTINTNSVDTNHAERDK					Mascot
2180.9407	2180.9417	0.001	0	53	71	FNDFDGSFTFDEKNPSADK					Mascot
2271.114	2271.1226	0.0086	4	94	114	SGDFLNVSKNPTATFESTEVK					Mascot
2284.1853	2284.1873	0.002	1	171	191	MDLGPASQEVELLLSVEGIRQ					Mascot
2300.1802	2300.1858	0.0056	2	171	191	MDLGPASQEVELLLSVEGIRQ	77	100	Oxidation (M)[1]		Mascot
2300.1802	2300.1858	0.0056	2	171	191	MDLGPASQEVELLLSVEGIRQ			Oxidation (M)[1]		Mascot
2395.1677	2395.1672	-0.0005	0	141	163	LIGQGDDPWGGYRAGFEGSATLK					Mascot
2399.2639	2399.179	-0.0849	-35	4	27	KTLAALALGSALFTAGQAMAADYK			Oxidation (M)[19]		Mascot
2598.4097	2598.3318	-0.0779	-30	115	140	ANGDSADITGNLTLNGVTKPVTIKAK					Mascot

Rank	Protein Name	Accession No.	Protein Score	Protein Score C. I. %	Total Ion Score	Total Ion C. I. %
1	hypothetical protein PA0572 (ImpA)	gi|15595769|ref|NP_249263.1|	551	100	270	100

Peptide Information

Calc. Mass	Obsrv. Mass	± da	± ppm	Start	End	Sequence	Ion	C. I. %	Modification	Rank	Result Type

				Seq.	Seq.		Score				
980.4796	980.4695	-0.0101	-10	421	429	ELGSYAGQR					Mascot
1090.6003	1090.5908	-0.0095	-9	747	756	NAYNLIVAGR					Mascot
1172.6633	1172.6545	-0.0088	-8	124	135	VLASLSLEAGGR					Mascot
1244.6052	1244.5966	-0.0086	-7	636	646	TPAIQQECAAR			Carbamidomethyl (C)[8]		Mascot
1289.6848	1289.6757	-0.0091	-7	757	768	AEADPLAGVYKR					Mascot
1409.6655	1409.6587	-0.0068	-5	597	609	GSIDKDYGGDVQR					Mascot
1459.7805	1459.7722	-0.0083	-6	864	875	DQRPTFALWGIR	60	100			Mascot
1459.7805	1459.7722	-0.0083	-6	864	875	DQRPTFALWGIR					Mascot
1469.7747	1469.7681	-0.0066	-4	714	726	SGEISNQIFPLHK					Mascot
1488.8169	1488.8105	-0.0064	-4	360	373	AQNLFALPGTTSLR					Mascot
1562.7921	1562.7831	-0.009	-6	730	742	VLREFGQNLDDTR					Mascot
1597.8333	1597.8226	-0.0107	-7	161	176	AVQWLVNGDPGAATAK					Mascot
1620.7653	1620.7606	-0.0047	-3	769	782	LWEDPGTYALNGER					Mascot
1635.8085	1635.8008	-0.0077	-5	414	429	EAGAAQKELGSYAGQR					Mascot
1697.7799	1697.7939	0.014	8	331	345	TTCDDVPGLSDFSKR			Carbamidomethyl (C)[3]		Mascot
1714.8799	1714.8759	-0.004	-2	399	413	FQETFVADAIVGYVR	119	100			Mascot
1714.8799	1714.8759	-0.004	-2	399	413	FQETFVADAIVGYVR					Mascot
1726.8143	1726.8113	-0.003	-2	733	746	EFGQNLDDTRVNYR					Mascot
1742.9032	1742.8994	-0.0038	-2	469	485	IEDAGQASLAVGLNTQR					Mascot
1753.9343	1753.9242	-0.0101	-6	160	176	RAVQWLVNGDPGAATAK					Mascot
1832.8787	1832.8763	-0.0024	-1	691	706	GWGESHELGHNLQVNR					Mascot
1926.9821	1926.9818	-0.0003	0	714	729	SGEISNQIFPLHKDWR	91	100			Mascot
1926.9821	1926.9818	-0.0003	0	714	729	SGEISNQIFPLHKDWR					Mascot
1958.9258	1958.9781	0.0523	27	783	797	MAFYTQWVHYWADLK					Mascot
1988.0236	1988.0134	-0.0102	-5	161	179	AVQWLVNGDPGAATAKDFK					Mascot
2074.0576	2074.0579	0.0003	0	691	708	GWGESHELGHNLQVNRLK					Mascot
2205.1663	2205.1675	0.0012	1	747	767	NAYNLIVAGRAEADPLAGVYK					Mascot
2212.2043	2212.1941	-0.0102	-5	465	485	LSIRIEDAGQASLAVGLNTQR					Mascot
2367.0552	2367.0588	0.0036	2	196	219	SAGLQPADAACNALTDASCASTSK			Carbamidomethyl (C)[11,19]		Mascot
2410.3088	2410.3064	-0.0024	-1	136	159	GLAYGTNVLTQLSGTNAAHAPLLK					Mascot
2539.2715	2539.2766	0.0051	2	613	635	ELNEVFIDDAYTLAGFAIPNQAK					Mascot
2545.2681	2545.2737	0.0056	2	293	316	SVELGGAYGGQDPALVQQIVDGSWR					Mascot
2566.4099	2566.4131	0.0032	1	136	160	GLAYGTNVLTQLSGTNAAHAPLLKR					Mascot
2764.3113	2764.3167	0.0054	2	876	901	TSAAAQAQVAAYGFAEQPAFFYANNR					Mascot
2906.4563	2906.4641	0.0078	3	430	457	QQSMPVSGSEETLTLTLPSAQGFTAIGR					Mascot
2922.4514	2922.4692	0.0178	6	430	457	QQSMPVSGSEETLTLTLPSAQGFTAIGR			Oxidation (M)[4]		Mascot
3686.751	3686.7751	0.0241	7	876	909	TSAAAQAQVAAYGFAEQ PAFFYANNRTNEYSTVK					Mascot

Rank	Protein Name	Accession No.	Protein Score	Protein Score C. I. %	Total Ion Score	Total Ion C. I. %
1	probable bacteriophage protein (HR2) PA0620	gi|15595817|ref|NP_249311.1|	591	100	324	100

Peptide Information

Calc. Mass	Obsrv. Mass	± da	± ppm	Start Seq.	End Seq.	Sequence	Ion Score	C. I. %	Modification	Rank	Result Type
1014.5156	1014.5365	0.0209	21	432	438	EWLPWQR					Mascot
1046.5477	1046.531	-0.0167	-16	235	244	ADTDGKLAQK					Mascot
1071.6157	1071.6338	0.0181	17	342	351	LVSGVLAEQR					Mascot
1077.5687	1077.5878	0.0191	18	422	431	VSYAANPSIR					Mascot
1230.5803	1230.6028	0.0225	18	518	526	HSNTWFPWR	53	99.997			Mascot
1230.5803	1230.6028	0.0225	18	518	526	HSNTWFPWR					Mascot
1386.6814	1386.7025	0.0211	15	518	527	HSNTWFPWRR					Mascot
1422.7588	1422.7816	0.0228	16	248	261	ATTLAGYGITDALR					Mascot
1435.7539	1435.7738	0.0199	14	190	204	SIGLAPSGVTAGSYR					Mascot
1546.7122	1546.7312	0.019	12	516	526	CRHSNTWFPWR			Carbamidomethyl (C)[1]		Mascot
1598.8496	1598.8668	0.0172	11	173	189	ILAGNGLLGGGDLSADR					Mascot

1726.9446	1726.9628	0.0182	11	172	189	KILAGNGLLGGGDLSADR					Mascot
1755.0276	1755.0409	0.0133	8	342	357	LVSGVLAEQRLPVFAR					Mascot
1779.8159	1779.8307	0.0148	8	528	542	MWHGGDFNPSDYLLK					Mascot
1795.8108	1795.8208	0.01	6	528	542	MWHGGDFNPSDYLLK			Oxidation (M)[1]		Mascot
1801.9364	1801.9058	-0.0306	-17	323	341	VTAGMALAATDIPGLDASK					Mascot
1815.8812	1815.896	0.0148	8	676	691	NGYFAQAVLSWEAFGR	164	100			Mascot
1815.8812	1815.896	0.0148	8	676	691	NGYFAQAVLSWEAFGR					Mascot
1935.917	1935.9287	0.0117	6	527	542	RMWHGGDFNPSDYLLK					Mascot
1944.9927	1945.0045	0.0118	6	591	610	STIGAGVPATASLGASGWWR	107	100			Mascot
1944.9927	1945.0045	0.0118	6	591	610	STIGAGVPATASLGASGWWR					Mascot
1951.912	1951.9265	0.0145	7	527	542	RMWHGGDFNPSDYLLK			Oxidation (M)[2]		Mascot
2020.1073	2020.1107	0.0034	2	8	29	YGGLLTDIGAAALATASAAGKK					Mascot
2073.0664	2073.0718	0.0054	3	422	438	VSYAANPSIREWLPWQR					Mascot
2283.2495	2283.2168	-0.0327	-14	77	97	NANTLVAEVVLPVEVGGFWIR					Mascot
2395.2212	2395.2065	-0.0147	-6	248	271	ATTLAGYGITDALRVDGNAVSSSR					Mascot
2787.4211	2787.3948	-0.0263	-9	666	691	GATTTTAVIRNGYFAQAVLSWEAFGR					Mascot
2829.4277	2829.3877	-0.04	-14	591	618	STIGAGVPATASLGASGWWRDNDTGLIR					Mascot
3133.5693	3133.5264	-0.0429	-14	358	389	GLATAVSNSSDPNTATVPLMLTNHANGPVAGR					Mascot
3149.5645	3149.5168	-0.0477	-15	358	389	GLATAVSNSSDPNTATVPLMLTNHANGPVAGR			Oxidation (M)[20]		Mascot
3381.585	3381.5032	-0.0818	-24	487	515	AGLLHVYAASSNFIYQTYQAYDGESFYFR					Mascot

Rank	Protein Name	Accession No.	Protein Score	Protein Score C. I. %	Total Ion Score	Total Ion C. I. %
1	probable bacteriophage protein (FIR2), PA0622	gi|15595819|ref|NP_249313.1|	483	100	229	100

Peptide Information

Calc. Mass	Obsrv. Mass	± da	± ppm	Start Seq.	End Seq.	Sequence	Ion Score	C. I. %	Modification	Rank	Result Type
975.5118	975.5095	-0.0023	-2	119	126	SRFNAQPR					Mascot
1251.5865	1251.589	0.0025	2	261	270	DDGYRLWGNR					Mascot
1280.6844	1280.6849	0.0005	0	309	319	TYVKDVTEGLR					Mascot
1497.7697	1497.7744	0.0047	3	271	283	TLSSDSKWAFVTR					Mascot
1503.7227	1503.7269	0.0042	3	359	371	FTDVPPAENPNFR					Mascot
1510.87	1510.8734	0.0034	2	247	260	ANLLNNANIATIIR					Mascot
1719.8812	1719.8881	0.0069	4	2	17	SFFHGVTVTNVDIGAR	117	100			Mascot
1719.8812	1719.8881	0.0069	4	2	17	SFFHGVTVTNVDIGAR					Mascot
1785.9349	1785.9351	0.0002	0	284	299	VRTMDLVMDAILAGHK			Oxidation (M)[4]		Mascot
1801.9299	1801.927	-0.0029	-2	284	299	VRTMDLVMDAILAGHK			Oxidation (M)[4,8]		Mascot
1990.0127	1990.0143	0.0016	1	152	171	AIAILDGPNSTDEAAVAYAK					Mascot
2038.9498	2038.9515	0.0017	1	228	246	GVTGTSRPVEFLDGDETCR					Mascot
2077.0171	2077.0303	0.0132	6	178	196	LFMVDPGVQVWDSATNAAR					Mascot
2093.012	2093.0239	0.0119	6	178	196	LFMVDPGVQVWDSATNAAR			Oxidation (M)[3]		Mascot
2095.9712	2095.9939	0.0227	11	228	246	GVTGTSRPVEFLDGDETCR	21	96.116	Carbamidomethyl (C)[18]		Mascot
2095.9712	2095.9939	0.0227	11	228	246	GVTGTSRPVEFLDGDETCR			Carbamidomethyl (C)[18]		Mascot
2117.1099	2117.1199	0.01	5	247	265	ANLLNNANIATIIRDDGYR					Mascot
2142.0835	2142.0847	0.0012	1	286	304	TMDLVMDAILAGHKWAVDR					Mascot
2158.0784	2158.0747	-0.0037	-2	286	304	TMDLVMDAILAGHKWAVDR			Oxidation (M)[2]		Mascot
2249.113	2249.0667	-0.0463	-21	177	196	RLFMVDPGVQVWDSATNAAR			Oxidation (M)[4]		Mascot
2259.198	2259.2004	0.0024	1	150	171	LRAIAILDGPNSTDEAAVAYAK					Mascot
2276.1016	2276.1177	0.0161	7	54	75	DAAAAFGIGSSIYLACEAIYNR					Mascot
2294.1809	2294.1897	0.0088	4	127	149	LLVAPGHSAQQAVATAMDGLAEK			Oxidation (M)[17]		Mascot
2333.123	2333.1243	0.0013	1	54	75	DAAAAFGIGSSIYLACEAIYNR			Carbamidomethyl (C)[16]		Mascot
2461.218	2461.2422	0.0242	10	53	75	KDAAAAFGIGSSIYLACEAIYNR			Carbamidomethyl (C)[17]		Mascot
2547.3711	2547.384	0.0129	5	127	151	LLVAPGHSAQQAVATAMDGLAEKLR	91	100			Mascot
2547.3711	2547.384	0.0129	5	127	151	LLVAPGHSAQQAVATAMDGLAEKLR					Mascot
2563.366	2563.3826	0.0166	6	127	151	LLVAPGHSAQQAVATAMDGLAEKLR			Oxidation (M)[17]		Mascot
3105.5486	3105.5837	0.0351	11	324	352	DLKNQGAVINFEVYADPDLNSASQLAQGK					Mascot

3122.6326	3122.6545	0.0219	7	76	108	AQAVIVAVGVETAETPEAQASAVIGGISAAGER					Mascot
3201.5737	3201.6047	0.031	10	359	386	FTDVPPAENPNFRVEVTDQWLTEVLDVA					Mascot
3522.9241	3522.9568	0.0327	9	18	52	TIALPASSVIGLCDVFTPGAQASAKPNVPVLLTSK			Carbamidomethyl (C)[13]		Mascot

Rank	Protein Name	Accession No.	Protein Score	Protein Score C. I. %	Total Ion Score	Total Ion C. I. %
1	probable bacteriophage protein (PA0623)	gi|15595820|ref|NP_249314.1|	496	100	343	100

Peptide Information

Calc. Mass	Obsrv. Mass	± da	± ppm	Start Seq.	End Seq.	Sequence	Ion Score	C. I. %	Modification	Rank	Result Type
1063.5459	1063.5399	-0.006	-6	124	132	YAVAVSYYK					Mascot
1069.6364	1069.6289	-0.0075	-7	93	103	GASVPVVATLR					Mascot
1107.6157	1107.6056	-0.0101	-9	30	38	LAVKTEQYR					Mascot
1244.6416	1244.635	-0.0066	-5	151	162	AINGVDQLAGMR					Mascot
1260.6365	1260.6311	-0.0054	-4	151	162	AINGVDQLAGMR			Oxidation (M)[11]		Mascot
1356.7158	1356.7122	-0.0036	-3	104	115	GLLKEVDPGDWK					Mascot
1389.7008	1389.6935	-0.0073	-5	139	150	EVYEIDPVNGVR					Mascot
1732.8904	1732.8772	-0.0132	-8	124	138	YAVAVSYYKLEVDGR					Mascot
1756.901	1756.8971	-0.0039	-2	151	167	AINGVDQLAGMRNDLGL					Mascot
1772.896	1772.8942	-0.0018	-1	151	167	AINGVDQLAGMRNDLGL	36	99.867	Oxidation (M)[11]		Mascot
1772.896	1772.8942	-0.0018	-1	151	167	AINGVDQLAGMRNDLGL			Oxidation (M)[11]		Mascot
2024.8973	2024.9175	0.0202	10	39	58	AGGMDAPVSIDMGLEAMEAK			Oxidation (M)[4,12]		Mascot
2059.0454	2059.0444	-0.001	0	133	150	LEVDGREVYEIDPVNGVR	106	100			Mascot
2059.0454	2059.0444	-0.001	0	133	150	LEVDGREVYEIDPVNGVR					Mascot
2103.0293	2103.0039	-0.0254	-12	67	85	EALNFFGLADQSAFNGVFR	200	100			Mascot
2103.0293	2103.0039	-0.0254	-12	67	85	EALNFFGLADQSAFNGVFR					Mascot
2259.1304	2259.1077	-0.0227	-10	66	85	REALNFFGLADQSAFNGVFR					Mascot
2522.2461	2522.2073	-0.0388	-15	67	89	EALNFFGLADQSAFNGVFRGSFK					Mascot

Rank	Protein Name	Accession No.	Protein Score	Protein Score C. I. %	Total Ion Score	Total Ion C. I. %
1	hypothetical protein PA0633 (VF2)	gi|15595830|ref|NP_249324.1|	182	100	153	100

Peptide Information

Calc. Mass	Obsrv. Mass	± da	± ppm	Start Seq.	End Seq.	Sequence	Ion Score	C. I. %	Modification	Rank	Result Type
1410.7335	1410.7319	-0.0016	-1	62	75	TPGQATLGINADPR	33	99.784			Mascot
1410.7335	1410.7319	-0.0016	-1	62	75	TPGQATLGINADPR					Mascot
1580.8167	1580.8123	-0.0044	-3	82	95	LFQLSEKDGETSVK					Mascot
1736.9402	1736.933	-0.0072	-4	59	75	GLRTPGQATLGINADPR					Mascot
3028.5051	3028.5249	0.0198	7	96	123	WAIGWSDGIDVKPTVSTEGDDFVLPPAR	120	100			Mascot
3028.5051	3028.5249	0.0198	7	96	123	WAIGWSDGIDVKPTVSTEGDDFVLPPAR					Mascot

Rank	Protein Name	Accession No.	Protein Score	Protein Score C. I. %	Total Ion Score	Total Ion C. I. %
1	phosphate binding protein component of ABC	gi|15595885|ref|N	555	100	393	100

transporter (LapA) PA0688 **P_249379.1|**

Peptide Information

Calc. Mass	Obsrv. Mass	± da	± ppm	Start Seq.	End Seq.	Sequence	Ion Score	C. I. %	Modification	Rank	Result Type
1097.5521	1097.5519	-0.0002	0	186	194	HLNSICPTR			Carbamidomethyl (C)[6]		Mascot
1229.5909	1229.595	0.0041	3	195	205	FATNSTFTNAR	103	100			Mascot
1229.5909	1229.595	0.0041	3	195	205	FATNSTFTNAR					Mascot
1256.6117	1256.6112	-0.0005	0	174	185	TGSSGTTELFTR					Mascot
1746.9021	1746.9033	0.0012	1	154	169	TTWGQLLGTTDSTPIR	140	100			Mascot
1746.9021	1746.9033	0.0012	1	154	169	TTWGQLLGTTDSTPIR					Mascot
1787.9286	1787.9214	-0.0072	-4	170	185	IVYRTGSSGTTELFTR					Mascot
2100.0244	2100.0276	0.0032	2	50	71	GSADSILPANFSYAVTGSGTGK					Mascot
2184.0237	2184.0244	0.0007	0	133	153	DGNTTLNLTSAQLCDAFSGAK			Carbamidomethyl (C)[14]		Mascot
2278.219	2278.2253	0.0063	3	154	173	TTWGQLLGTTDSTPIRIVYR					Mascot
2308.1252	2308.1328	0.0076	3	186	205	HLNSICPTRFATNSTFTNAR			Carbamidomethyl (C)[6]		Mascot
2335.146	2335.1562	0.0102	4	174	194	TGSSGTTELFTRHLNSICPTR			Carbamidomethyl (C)[18]		Mascot
2412.3132	2412.3191	0.0059	2	206	230	LPAGGTLPSNWVGVAATSTVVSTVK					Mascot
2435.1797	2435.1863	0.0066	3	231	254	ATNGSLGYVSPDAVNINSNAEVSR					Mascot
2481.2673	2481.2725	0.0052	2	328	351	HYGGTTTNAAVAAHGFIPLTPAWK					Mascot
2605.3328	2605.3347	0.0019	1	255	281	VNGNLPTQANVSTALGSVAPPANAADR	150	100			Mascot
2605.3328	2605.3347	0.0019	1	255	281	VNGNLPTQANVSTALGSVAPPANAADR					Mascot
2732.3196	2732.3279	0.0083	3	352	377	SAIVSAFYTGTSENLAIGNTNVCNTK			Carbamidomethyl (C)[23]		Mascot
3103.5767	3103.5818	0.0051	2	255	286	VNGNLPTQANVSTALGSVAPPANAADRADPSK					Mascot
3622.8862	3622.9023	0.0161	4	195	230	FATNSTFTNARLPAGGTLPSNWVGVAATSTVVSTVK					Mascot

Rank	Protein Name	Accession No.	Protein Score	Protein Score C. I. %	Total Ion Score	Total Ion C. I. %
1	hypothetical protein PA0807 (AmpDh3)	gi|15596004|ref|NP_249498.1|	535	100	277	100

Peptide Information

Calc. Mass	Obsrv. Mass	± da	± ppm	Start Seq.	End Seq.	Sequence	Ion Score	C. I. %	Modification	Rank	Result Type
923.4944	923.4645	-0.0299	-32	11	18	TTTPYGKR					Mascot
934.4992	934.4695	-0.0297	-32	196	203	ADLLEAFR					Mascot
956.4584	956.429	-0.0294	-31	183	189	YREGFER					Mascot
1197.5912	1197.5594	-0.0318	-27	77	87	AWHAGVSGWAR					Mascot
1205.6161	1205.5824	-0.0337	-28	67	76	IFNLVAEEDR					Mascot
1223.649	1223.6157	-0.0333	-27	142	153	NVVGHSDIAVGR					Mascot
1275.6039	1275.5742	-0.0297	-23	1	10	MLTIDYNSYR					Mascot
1291.5988	1291.5725	-0.0263	-20	1	10	MLTIDYNSYR			Oxidation (M)[1]		Mascot
1351.744	1351.7124	-0.0316	-23	142	154	NVVGHSDIAVGRK					Mascot
1475.7676	1475.7422	-0.0254	-17	130	141	NILQRYPDMTPK					Mascot
1491.7625	1491.7411	-0.0214	-14	130	141	NILQRYPDMTPK			Oxidation (M)[9]		Mascot
1569.8384	1569.8203	-0.0181	-12	190	203	DGLPPRADLLEAFR					Mascot
1575.6598	1575.6359	-0.0239	-15	106	118	DDDGVFTFPDYER	51	99.997			Mascot
1575.6598	1575.6359	-0.0239	-15	106	118	DDDGVFTFPDYER					Mascot
1794.9789	1794.9618	-0.0171	-10	21	36	FLVLHYTALDFAASVK					Mascot
1829.8341	1829.8076	-0.0265	-14	165	180	ELYEAGIGAWYDDATR					Mascot
1856.9712	1856.943	-0.0282	-15	89	105	DNLNDTSIGIEIVNLAR					Mascot
2013.0723	2013.0591	-0.0132	-7	88	105	RDNLNDTSIGIEIVNLAR	87	100			Mascot
2013.0723	2013.0591	-0.0132	-7	88	105	RDNLNDTSIGIEIVNLAR					Mascot
2050.1484	2050.1157	-0.0327	-16	19	36	VRFLVLHYTALDFAASVK					Mascot
2219.1382	2219.1104	-0.0278	-13	204	223	LYGYALPATVDDAYFASLLR	139	100			Mascot
2219.1382	2219.1104	-0.0278	-13	204	223	LYGYALPATVDDAYFASLLR					Mascot

2281.1611	2281.1548	-0.0063	-3	37	58	ALTTGAASAHYLIPAPHD PSYK					Mascot
2330.0935	2330.0701	-0.0234	-10	106	125	DDDGVFTFPDYERSQINALK					Mascot
2384.1895	2384.1865	-0.003	-1	67	87	IFNLVAEEDRAWHAGVSGWAR					Mascot
3673.7632	3673.7751	0.0119	3	224	255	AFQMHFRPENYDGALDVETAAILYALNEKYPA			Oxidation (M)[4]		Mascot

Rank	Protein Name	Accession No.	Protein Score	Protein Score C. I. %	Total Ion Score	Total Ion C. I. %
1	hemolytic phospholipase C precursor (PlcH) PA0844	gi|15596041|ref|NP_249535.1|	611	100	312	100

Peptide Information

Calc. Mass	Obsrv. Mass	± da	± ppm	Start Seq.	End Seq.	Sequence	Ion Score	C. I. %	Modification	Rank	Result Type
919.4268	919.4315	0.0047	5	281	287	DSDLWQR					Mascot
971.4581	971.4596	0.0015	2	234	241	VYQEGGYR					Mascot
972.4461	972.4539	0.0078	8	495	501	YWSYEPK					Mascot
974.5054	974.4999	-0.0055	-6	225	233	LSAAGVDWR					Mascot
1041.58	1041.5808	0.0008	1	638	646	NGNLQLNIR					Mascot
1432.7219	1432.7274	0.0055	4	434	445	FGVVEENISPWR					Mascot
1459.7539	1459.7603	0.0064	4	416	428	VSAEVFDHTSVLR					Mascot
1540.7332	1540.7456	0.0124	8	602	614	YAFEVHGPNGFFR					Mascot
1548.7343	1548.7422	0.0079	5	63	75	SFDHYFGHLNGVR	86	100			Mascot
1548.7343	1548.7422	0.0079	5	63	75	SFDHYFGHLNGVR					Mascot
1584.838	1584.8453	0.0073	5	336	349	VLEALTSNPEVWAR					Mascot
1588.823	1588.8323	0.0093	6	434	446	FGVVEENISPWRR					Mascot
1675.8473	1675.8497	0.0024	1	651	665	LPCSVTVTPNPAYTR			Carbamidomethyl (C)[3]		Mascot
1681.8445	1681.8564	0.0119	7	85	97	RQDGKPVWYQNYK					Mascot
1701.8918	1701.8973	0.0055	3	413	428	GGKVSAEVFDHTSVLR					Mascot
1942.9446	1942.9548	0.0102	5	480	494	KEDAYWQQFYRPSPK	112	100			Mascot
1942.9446	1942.9548	0.0102	5	480	494	KEDAYWQQFYRPSPK					Mascot
1960.0175	1959.9827	-0.0348	-18	671	686	YELEPNQAISEVWLLR					Mascot
2041.9185	2041.9297	0.0112	5	258	273	LQEQNNYDCNALAWFR			Carbamidomethyl (C)[9]		Mascot
2081.0808	2081.094	0.0132	6	45	62	TGTIQDVQHVVILMQENR					Mascot
2097.0757	2097.0847	0.009	4	45	62	TGTIQDVQHVVILMQENR			Oxidation (M)[14]		Mascot
2100.0225	2100.0833	0.0608	29	242	257	SSSLWYLYVDAYWKYR					Mascot
2116.1187	2116.1189	0.0002	0	670	686	RYELEPNQAISEVWLLR					Mascot
2161.0447	2161.0549	0.0102	5	568	586	FYTVTSYPVVQESGEELGR	114	100			Mascot
2161.0447	2161.0549	0.0102	5	568	586	FYTVTSYPVVQESGEELGR					Mascot
2235.0479	2235.064	0.0161	7	63	81	SFDHYFGHLNGVRGFNDPR					Mascot
2248.1931	2248.2017	0.0086	4	709	730	LAGHVETGKPSRSDPLLDIAAT					Mascot
2361.083	2361.0979	0.0149	6	256	273	YRLQEQNNYDCNALAWFR			Carbamidomethyl (C)[11]		Mascot
2504.3984	2504.4143	0.0159	6	509	532	GQRPTLAVPYQLHATLALDIAAGK					Mascot
2518.1958	2518.2122	0.0164	7	687	708	SSQGWYDLSVTASNTEANYLRR					Mascot
2654.3257	2654.3435	0.0178	7	615	637	EFHGNLHLAAQMARPEV SVTYQR					Mascot
2773.5835	2773.592	0.0085	3	509	534	GQRPTLAVPYQLHATLALDIAAGKLR					Mascot
2791.3943	2791.4353	0.041	15	535	561	LTLGNDGMSLPGNPQGHSAAVFQVQPR					Mascot
2807.3894	2807.4202	0.0308	11	535	561	LTLGNDGMSLPGNPQGHSAAVFQVQPR			Oxidation (M)[8]		Mascot
2926.3767	2926.4072	0.0305	10	109	133	VTSAQWVSSQNHEWSAFHAIWNQGR					Mascot
2979.3735	2979.3955	0.022	7	86	108	QDGKPVWYQNYKYEFSPYHWDTK					Mascot
3465.6226	3465.6636	0.041	12	447	479	AVCGDLTSLFDFQGAGDTQVAPDLTNVPQSDAR			Carbamidomethyl (C)[3]		Mascot

Rank	Protein Name	Accession No.	Protein Score	Protein Score C. I. %	Total Ion Score	Total Ion C. I. %
1	chitin-binding protein CbpD precursor, PA0852	gi|15596049|ref|NP_249543.1|	488	100	256	100

Peptide Information

Calc. Mass	Obsrv. Mass	± da	± ppm	Start Seq.	End Seq.	Sequence	Ion Score	C. I. %	Modification	Rank	Result Type
1054.6044	1054.5959	-0.0085	-8	263	271	QWPLALAQK					Mascot
1096.535	1096.5319	-0.0031	-3	133	140	YFDFYITK					Mascot
1102.6731	1102.6721	-0.001	-1	94	103	ALFKGLNLAR					Mascot
1169.5626	1169.557	-0.0056	-5	369	378	GWDLYYAPGK					Mascot
1214.6429	1214.6504	0.0075	6	186	194	HVIYNVWQR	67	100			Mascot
1214.6429	1214.6504	0.0075	6	186	194	HVIYNVWQR					Mascot
1276.6393	1276.6385	-0.0008	-1	239	249	LFDAQGRDAQR					Mascot
1440.7805	1440.7834	0.0029	2	225	238	AQQDLPAGATVTLR	109	100			Mascot
1440.7805	1440.7834	0.0029	2	225	238	AQQDLPAGATVTLR					Mascot
1500.807	1500.8134	0.0064	4	183	194	TGKHVIYNVWQR	80	100			Mascot
1500.807	1500.8134	0.0064	4	183	194	TGKHVIYNVWQR					Mascot
1509.7042	1509.704	-0.0002	0	36	48	VYGCFLEGPENPK			Carbamidomethyl (C)[4]		Mascot
1687.7356	1687.736	0.0004	0	356	368	YQCKPYPNSGWCK			Carbamidomethyl (C)[3,12]		Mascot
1737.899	1737.8983	-0.0007	0	246	262	DAQRHSLTLAQGANGAK					Mascot
1843.8367	1843.8379	0.0012	1	355	368	RYQCKPYPNSGWCK			Carbamidomethyl (C)[4,13]		Mascot
1887.9388	1887.9427	0.0039	2	125	140	ASAPHATRYFDFYITK					Mascot
1893.8945	1893.9131	0.0186	10	167	182	LENGTYRMNCPLPQGK			Carbamidomethyl (C)[10], Oxidation (M)[8]		Mascot
2026.936	2026.9353	-0.0007	0	36	53	VYGCFLEGPENPKSAACK			Carbamidomethyl (C)[4,17]		Mascot
2171.0403	2171.0449	0.0046	2	331	349	VDFDYPQGLQQYDAGTVVR					Mascot
2228.1782	2228.178	-0.0002	0	225	245	AQQDLPAGATVTLRLFDAQGR					Mascot
2301.0823	2301.1221	0.0398	17	104	124	SDWPSTAIAPDASGNFQFVYK					Mascot
2703.3008	2703.3025	0.0017	1	305	330	QAGYRFQVDIELPVEGGGEQPGGDGK					Mascot
2838.2803	2838.2952	0.0149	5	356	378	YQCKPYPNSGWCKGWDLYYAPGK			Carbamidomethyl (C)[3,12]		Mascot
2921.4026	2921.4131	0.0105	4	141	166	DGYNPEKPLAWSDLEPAPFCSITSVK			Carbamidomethyl (C)[20]		Mascot
3092.4861	3092.4844	-0.0017	-1	104	132	SDWPSTAIAPDASGNFQFVYKASAPHATR					Mascot
3478.7449	3478.7734	0.0285	8	272	304	VNQDSTLVNIGVLDAYGAVSPVASSQDNQVYVR					Mascot

Rank	Protein Name	Accession No.	Protein Score	Protein Score C. I. %	Total Ion Score	Total Ion C. I. %
1	arginine/ornithine binding protein AotJ PA0888	gi|15596085|ref|NP_249579.1|	545	100	373	100

Peptide Information

Calc. Mass	Obsrv. Mass	± da	± ppm	Start Seq.	End Seq.	Sequence	Ion Score	C. I. %	Modification	Rank	Result Type
990.5366	990.5556	0.019	19	230	238	FNAAIDALR					Mascot
1167.6156	1167.631	0.0154	13	210	220	YFGEGVGIAVR					Mascot
1360.7332	1360.7462	0.013	10	230	242	FNAAIDALRANGK					Mascot
1366.7001	1366.715	0.0149	11	197	209	GYAFVGPQLTDAK					Mascot
1405.7726	1405.7837	0.0111	8	28	40	IGIEAAYPPFSLK					Mascot
1561.822	1561.8402	0.0182	12	145	159	YASAELTPAGVEVVR					Mascot
1644.8632	1644.8715	0.0083	5	67	80	WVEQEFDGLIPALK					Mascot
1675.8075	1675.8206	0.0131	8	100	113	SVDFTNKYYNTPAR	102	100			Mascot
1675.8075	1675.8206	0.0131	8	100	113	SVDFTNKYYNTPAR					Mascot
1692.8837	1692.8931	0.0094	6	84	98	IDAILSSMTITDERK					Mascot
1695.7755	1695.8193	0.0438	26	160	174	YNSQQEANMDLVAGR					Mascot
1708.8787	1708.8861	0.0074	4	84	98	IDAILSSMTITDERK			Oxidation (M)[8]		Mascot
1711.7704	1711.8102	0.0398	23	160	174	YNSQQEANMDLVAGR			Oxidation (M)[9]		Mascot
1810.8282	1810.8372	0.009	5	245	259	QIQDKYFSFDVYGSN					Mascot
1838.9282	1838.9348	0.0066	4	192	209	TDAGKGYAFVGPQLTDAK					Mascot
1900.0327	1900.0442	0.0115	6	67	82	WVEQEFDGLIPALKVR					Mascot
2149.0884	2149.0938	0.0054	3	139	159	GSTADRYASAELTPAGVEVVR	112	100			Mascot
2149.0884	2149.0938	0.0054	3	139	159	GSTADRYASAELTPAGVEVVR					Mascot
2515.2979	2515.2871	-0.0108	-4	197	220	GYAFVGPQLTDAKYFGEGVGIAVR	160	100			Mascot

2515.2979	2515.2871	-0.0108	-4	197	220	GYAFVGPQLTDAKYFGEGVGIAVR					Mascot
3481.6902	3481.6375	-0.0527	-15	160	191	YNSQQEANMDLVAGRLDAVVADSVNLEDGFL					Mascot

Rank	Protein Name	Accession No.	Protein Score	Protein Score C. I. %	Total Ion Score	Total Ion C. I. %
1	**flagellar hook-associated protein (FlgK) PA1086**	**gi|15596283|ref|NP_249777.1|**	**550**	**100**	**318**	**100**

Peptide Information

Calc. Mass	Obsrv. Mass	± da	± ppm	Start Seq.	End Seq.	Sequence	Ion Score	C. I. %	Modification	Rank	Result Type
916.521	916.518	-0.003	-3	614	622	VGTLTAQAR					Mascot
1018.5792	1018.5787	-0.0005	0	422	429	FTLQPTRR					Mascot
1192.6069	1192.6042	-0.0027	-2	440	451	NASQLAFAGSAR					Mascot
1214.674	1214.6654	-0.0086	-7	131	142	EAVLAQAQGLSK					Mascot
1220.6633	1220.6613	-0.002	-2	66	76	LASDFLTGQLR	74	100			Mascot
1220.6633	1220.6613	-0.002	-2	66	76	LASDFLTGQLR					Mascot
1256.5906	1256.5922	0.0016	1	366	376	LDFDGTNFTAR					Mascot
1298.6699	1298.6656	-0.0043	-3	188	200	SAGAVPNDLLDAR					Mascot
1357.6635	1357.6847	0.0212	16	143	153	TFNTLYDQLDK					Mascot
1376.7645	1376.7622	-0.0023	-2	65	76	RLASDFLTGQLR					Mascot
1412.6917	1412.6937	0.002	1	366	377	LDFDGTNFTARR					Mascot
1421.757	1421.7539	-0.0031	-2	270	283	LVSGGQMGGLLAYR					Mascot
1437.7518	1437.7532	0.0014	1	270	283	LVSGGQMGGLLAYR			Oxidation (M)[7]		Mascot
1490.8213	1490.8201	-0.0012	-1	294	307	LGQLALTFADTVNK					Mascot
1822.9293	1822.9208	-0.0085	-5	254	269	YQVQLTLGDSTQNVTR					Mascot
1868.946	1868.9407	-0.0053	-3	188	205	SAGAVPNDLLDARDEAVR					Mascot
1872.9449	1872.9376	-0.0073	-4	319	336	AGANLFGDINDPDITALR	87	100			Mascot
1872.9449	1872.9376	-0.0073	-4	319	336	AGANLFGDINDPDITALR					Mascot
1925.9352	1925.9211	-0.0141	-7	113	130	FFSALQTAAQNPSSTEAR	157	100			Mascot
1925.9352	1925.9211	-0.0141	-7	113	130	FFSALQTAAQNPSSTEAR					Mascot
1943.9417	1943.9281	-0.0136	-7	519	538	DGNALPGSPTLNSGTSNSVR					Mascot
2075.978	2075.9749	-0.0031	-1	359	376	LNSSDFRLDFDGTNFTAR					Mascot
2208.1255	2208.1094	-0.0161	-7	430	451	GASDIETTLKNASQLAFAGSAR					Mascot
2246.0684	2246.0525	-0.0159	-7	400	421	DANGVDQGFSVTLDQLPAAGDR					Mascot
2284.2295	2284.1965	-0.033	-14	319	340	AGANLFGDINDPDITALRVLAK					Mascot
2446.1919	2446.1919	0	0	377	399	RLGDDASMQVTVSGTGPYTLSFK			Oxidation (M)[8]		Mascot
2560.2712	2560.2439	-0.0273	-11	270	293	LVSGGQMGGLLAYRDTVLDSSYNK			Oxidation (M)[7]		Mascot
3121.5913	3121.4946	-0.0967	-31	113	142	FFSALQTAAQNPSSTEAREAVLAQAQGLSK					Mascot
3497.7141	3497.6287	-0.0854	-24	580	613	NALNLNALQTKPTVGGTDSTGSTYNDAYGGLVER					Mascot

Rank	Protein Name	Accession No.	Protein Score	Protein Score C. I. %	Total Ion Score	Total Ion C. I. %
1	**flagellin type B (FliC), PA1092**	**gi|15596289|ref|NP_249783.1|**	**576**	**100**	**366**	**100**

Peptide Information

Calc. Mass	Obsrv. Mass	± da	± ppm	Start Seq.	End Seq.	Sequence	Ion Score	C. I. %	Modification	Rank	Result Type
943.4955	943.4907	-0.0048	-5	417	425	ADLGAVQNR					Mascot
1054.5164	1054.5098	-0.0066	-6	126	135	ISDTTTFGGR					Mascot
1144.5779	1144.5692	-0.0087	-8	225	235	MDGAIPNLSAR					Mascot
1159.5702	1159.5626	-0.0076	-7	43	53	DDAAGLQISNR					Mascot
1160.5729	1160.5663	-0.0066	-6	225	235	MDGAIPNLSAR			Oxidation (M)[1]		Mascot
1182.6113	1182.6064	-0.0049	-4	126	136	ISDTTTFGGRK					Mascot
1215.6328	1215.6268	-0.006	-5	115	125	EVAAQQAELTR					Mascot

Calc. Mass	Obsrv. Mass	± da	± ppm	Start Seq.	End Seq.	Sequence	Ion Score	C. I. %	Modification	Rank	Result Type
1307.6589	1307.6528	-0.0061	-5	325	337	VQGSDGKFEAAAK					Mascot
1372.7543	1372.7468	-0.0075	-5	54	66	LSNQISGLNVATR					Mascot
1506.791	1506.7844	-0.0066	-4	309	324	FGAQTGTATAGQVAVK					Mascot
1580.8167	1580.8163	-0.0004	0	447	461	IKDTDFAAETAALSK					Mascot
1615.8762	1615.8718	-0.0044	-3	2	16	ALTVNTNIASLNTQR	144	100			Mascot
1615.8762	1615.8718	-0.0044	-3	2	16	ALTVNTNIASLNTQR					Mascot
1646.8093	1646.8049	-0.0044	-3	17	31	NLNASSNDLNTSLQR					Mascot
1656.8738	1656.8706	-0.0032	-2	220	235	AIAEKMDGAIPNLSAR					Mascot
1672.8613	1672.858	-0.0033	-2	38	53	INSAKDDAAGLQISNR					Mascot
1726.9446	1726.9425	-0.0021	-1	110	125	AALQKEVAAQQAELTR					Mascot
1860.9047	1860.9073	0.0026	1	428	444	NTIDNLTNISENATNAR					Mascot
1902.9264	1902.9333	0.0069	4	92	109	IRDLALQSANGSNSDADR					Mascot
2136.0679	2136.0671	-0.0008	0	426	444	FKNTIDNLTNISENATNAR	174	100			Mascot
2136.0679	2136.0671	-0.0008	0	426	444	FKNTIDNLTNISENATNAR					Mascot
2145.053	2145.0603	0.0073	3	94	114	DLALQSANGSNSDADRAALQK					Mascot
2402.3137	2402.3257	0.012	5	285	308	LGITASINDKGVLTITSATGENVK					Mascot
2613.3228	2613.3311	0.0083	3	67	91	NANDGISLAQTAEGALQQSTNILQR	49	99.994			Mascot
2613.3228	2613.3311	0.0083	3	67	91	NANDGISLAQTAEGALQQSTNILQR					Mascot
2858.6211	2858.6345	0.0134	5	462	488	NQVLQQAGTAILAQANQLPQAVLSLLR					Mascot
3113.5708	3113.5935	0.0227	7	385	416	SSVASVDISTADGAQNAIAVVDNALAAIDAQR					Mascot

Rank	Protein Name	Accession No.	Protein Score	Protein Score C. I. %	Total Ion Score	Total Ion C. I. %
2	flagellar capping protein FliD PA1094	gi|15596291|ref|NP_249785.1|	84	99.998		

Peptide Information

Calc. Mass	Obsrv. Mass	± da	± ppm	Start Seq.	End Seq.	Sequence	Ion Score	C. I. %	Modification	Rank	Result Type
1295.7318	1295.728	-0.0038	-3	108	120	IALQAIADPANAK					Mascot
1343.7278	1343.7288	0.001	1	229	241	AANGEITVDGLKR					Mascot
1542.885	1542.8849	-0.0001	0	261	275	AVTEAGKPITLTVSR					Mascot
1577.7765	1577.776	-0.0005	0	160	175	EAGVSATIITDNSGSR					Mascot
1759.9008	1759.9028	0.002	1	135	151	LPAITVDSSNNTLAGMR					Mascot
1775.8956	1775.8965	0.0009	1	135	151	LPAITVDSSNNTLAGMR			Oxidation (M)[16]		Mascot
1938.9655	1938.9623	-0.0032	-2	242	260	SIASNSVSDVIDGVSFDVK					Mascot
2095.0667	2095.0693	0.0026	1	241	260	RSIASNSVSDVIDGVSFDVK					Mascot
2375.1797	2375.1873	0.0076	3	152	175	DAINQAGKEAGVSATIITDNSGSR					Mascot
2383.21	2383.2144	0.0044	2	84	107	ASATQSAVAGTYQIQVNSLATSSK					Mascot
2563.2158	2563.2224	0.0066	3	191	216	VEVSDDGSGGNTSLSQLAFDPATAPK					Mascot
3209.6106	3209.6331	0.0225	7	121	151	FNSGTLNISVGDTKLPAITVDSSNNTLAGMR			Oxidation (M)[30]		Mascot
3269.6497	3269.6924	0.0427	13	76	107	SSNEDILKASATQSAVAGTYQIQVNSLATSSK					Mascot

Rank	Protein Name	Accession No.	Protein Score	Protein Score C. I. %	Total Ion Score	Total Ion C. I. %
1	exotoxin A precursor (Eta/ToxA) PA1148	gi|15596345|ref|NP_249839.1|	473	100	245	100

Peptide Information

Calc. Mass	Obsrv. Mass	± da	± ppm	Start Seq.	End Seq.	Sequence	Ion Score	C. I. %	Modification	Rank	Result Type
912.5737	912.5737	0	0	518	525	IRNGALLR					Mascot
926.473	926.4743	0.0013	1	531	538	SSLPGFYR					Mascot
989.6141	989.6111	-0.003	-3	319	327	LVALYLAAR					Mascot

1090.5276	1090.5258	-0.0018	-2	438	446	GTQNWTVER					Mascot
1103.548	1103.545	-0.003	-3	484	492	SQDLDAIWR					Mascot
1106.5378	1106.5288	-0.009	-8	211	219	RWSEWASGK					Mascot
1244.6844	1244.6854	0.001	1	363	374	LALTLAAAESER					Mascot
1294.7113	1294.7129	0.0016	1	100	111	LEGGVEPNKPVR					Mascot
1330.6863	1330.6874	0.0011	1	482	492	ARSQDLDAIWR					Mascot
1357.7223	1357.7219	-0.0004	0	328	338	LSWNQVDQVIR					Mascot
1365.7737	1365.7711	-0.0026	-2	589	601	TVVIPSAIPTDPR					Mascot
1584.8228	1584.8171	-0.0057	-4	539	554	TGLTLAAPEAAGEVER					Mascot
1612.7424	1612.7751	0.0327	20	239	251	CNLDDTWEGKIYR					Mascot
1646.9224	1646.9215	-0.0009	-1	363	377	LALTLAAAESERFVR					Mascot
1669.7639	1669.7633	-0.0006	0	239	251	CNLDDTWEGKIYR			Carbamidomethyl (C)[1]		Mascot
1692.7799	1692.7834	0.0035	2	305	318	GWEQLEQCGYPVQR					Mascot
1749.8013	1749.8029	0.0016	1	305	318	GWEQLEQCGYPVQR			Carbamidomethyl (C)[8]		Mascot
1772.9752	1772.9723	-0.0029	-2	83	99	LAIDNALSITSDGLTIR					Mascot
2103.9617	2103.9619	0.0002	0	418	437	NYPTGAEFLGDGGDISFSTR	96	100			Mascot
2103.9617	2103.9619	0.0002	0	418	437	NYPTGAEFLGDGGDISFSTR					Mascot
2250.1587	2250.157	-0.0017	-1	220	238	VLCLLDPLDGVYNYLAQQR			Carbamidomethyl (C)[3]		Mascot
2262.2029	2262.2029	0	0	120	139	GSWSLNWLVPIGHEKPSNIK					Mascot
2281.2776	2281.2756	-0.002	-1	252	272	VLAGNPAKHDLDIKPTVISHR					Mascot
2423.1909	2423.1934	0.0025	1	339	362	NALASPGSGGDLGEAIREQPEQAR					Mascot
2488.1416	2488.1421	0.0005	0	493	515	GFYIAGDPALAYGYAQDQEPDAR	121	100			Mascot
2488.1416	2488.1421	0.0005	0	493	515	GFYIAGDPALAYGYAQDQEPDAR					Mascot
2574.314	2574.3191	0.0051	2	458	481	GYVFVGYHGTFLEAAQSIVFGGVR					Mascot
2701.2642	2701.2632	-0.001	0	493	517	GFYIAGDPALAYGYAQDQEPDARGR					Mascot
2959.4512	2959.46	0.0088	3	180	207	AHESNEMQPTLAISHAGVSVVMAQAQPR					Mascot
2974.4993	2974.5034	0.0041	1	273	299	LHFPEGGSLAALTAHQACHLPLETFTR	28	99.478	Carbamidomethyl (C)[18]		Mascot
2974.4993	2974.5034	0.0041	1	273	299	LHFPEGGSLAALTAHQACHLPLETFTR			Carbamidomethyl (C)[18]		Mascot
2991.4412	2991.4773	0.0361	12	180	207	AHESNEMQPTLAISHAGVSVVMAQAQPR			Oxidation (M)[7,22]		Mascot

Rank	Protein Name	Accession No.	Protein Score	Protein Score C. I. %	Total Ion Score	Total Ion C. I. %
1	**LasA protease precursor, PA1871**	**gi|15597068|ref|NP_250562.1|**	**254**	**100**	**211**	**100**

Peptide Information

Calc. Mass	Obsrv. Mass	± da	± ppm	Start Seq.	End Seq.	Sequence	Ion Score	C. I. %	Modification	Rank	Result Type
1527.6493	1527.6484	-0.0009	-1	380	392	INVGTSNYDNDCR			Carbamidomethyl (C)[12]		Mascot
1683.7504	1683.7583	0.0079	5	380	393	INVGTSNYDNDCRR			Carbamidomethyl (C)[12]		Mascot
1732.8766	1732.8796	0.003	2	280	296	WGSATYSVVAAHAGTVR	118	100			Mascot
1732.8766	1732.8796	0.003	2	280	296	WGSATYSVVAAHAGTVR					Mascot
2867.3611	2867.3767	0.0156	5	394	418	YYFYNQSAGTTHCAFRPLYNPGLAL					Mascot
2924.3823	2924.3813	-0.001	0	394	418	YYFYNQSAGTTHCAFRPLYNPGLAL	92	100	Carbamidomethyl (C)[13]		Mascot
2924.3823	2924.3813	-0.001	0	394	418	YYFYNQSAGTTHCAFRPLYNPGLAL			Carbamidomethyl (C)[13]		Mascot
3080.4836	3080.4719	-0.0117	-4	393	418	RYYFYNQSAGTTHCAFRPLYNPGLAL			Carbamidomethyl (C)[14]		Mascot
3362.5496	3362.5396	-0.01	-3	305	334	VTHPSGWATNYYHMDQIQVSNGQQVSADTK					Mascot
3378.5444	3378.5535	0.0091	3	305	334	VTHPSGWATNYYHMDQIQVSNGQQVSADTK			Oxidation (M)[14]		Mascot
3482.4734	3482.4592	-0.0142	-4	249	279	QGYSWQPNGAHSNTGSGYPYSSFDASYDWPR					Mascot

Rank	Protein Name	Accession No.	Protein Score	Protein Score C. I. %	Total Ion Score	Total Ion C. I. %
1	**hypothetical protein PA2377**	**gi|15597573|ref|NP_251067.1|**	**359**	**100**	**168**	**100**

Peptide Information

Calc. Mass	Obsrv. Mass	± da	± ppm	Start Seq.	End Seq.	Sequence	Ion Score	C. I. %	Modification	Rank	Result Type
937.5253	937.5095	-0.0158	-17	166	173	YKPVGFAR	55	99.998			Mascot
937.5253	937.5095	-0.0158	-17	166	173	YKPVGFAR					Mascot
955.5107	955.4933	-0.0174	-18	244	251	RYGSGFLR					Mascot
962.4625	962.4471	-0.0154	-16	370	376	WMADRQR					Mascot
978.4574	978.4401	-0.0173	-18	370	376	WMADRQR			Oxidation (M)[2]		Mascot
1102.6003	1102.5754	-0.0249	-23	202	210	LPAERYPTR	46	99.984			Mascot
1102.6003	1102.5754	-0.0249	-23	202	210	LPAERYPTR					Mascot
1155.6091	1155.5939	-0.0152	-13	383	392	HMAQIFGPVR					Mascot
1171.6041	1171.5862	-0.0179	-15	383	392	HMAQIFGPVR			Oxidation (M)[2]		Mascot
1199.6459	1199.6289	-0.017	-14	211	220	FADYLKPAFK					Mascot
1228.7161	1228.7002	-0.0159	-13	252	262	ALAAQKPNFLR	69	100			Mascot
1228.7161	1228.7002	-0.0159	-13	252	262	ALAAQKPNFLR					Mascot
1311.7103	1311.6971	-0.0132	-10	382	392	RHMAQIFGPVR					Mascot
1327.7052	1327.6941	-0.0111	-8	382	392	RHMAQIFGPVR			Oxidation (M)[3]		Mascot
1592.853	1592.8541	0.0011	1	345	360	ADVGEAPGLPPLESLK					Mascot
1819.9912	1819.9816	-0.0096	-5	343	360	ARADVGEAPGLPPLESLK					Mascot
2093.1238	2093.1125	-0.0113	-5	55	75	KPALGPETAGATLVIPGETDR					Mascot
2134.0603	2134.0461	-0.0142	-7	324	342	LLLSYLGSAEFQSSYGGWR					Mascot
2159.1494	2159.1406	-0.0088	-4	113	131	FPGVTLDVTVDLSKFHDLR					Mascot
2182.1389	2182.1121	-0.0268	-12	107	126	TAFESRFPGVTLDVTVDLSK					Mascot
2537.2922	2537.2793	-0.0129	-5	223	243	LVLTYPHDDDAVLYVYDKLER					Mascot
2676.3628	2676.3621	-0.0007	0	345	369	ADVGEAPGLPPLESLKNVDVHDFTR					Mascot
2682.2471	2682.241	-0.0061	-2	177	201	GYADPDGAYVTAYNNAFVPTYASVR					Mascot
2810.342	2810.3232	-0.0188	-7	176	201	KGYADPDGAYVTAYNNAFVPTYASVR					Mascot
2867.5625	2867.5601	-0.0024	-1	55	82	KPALGPETAGATLVIPGETDRLASLYAR					Mascot

Rank	Protein Name	Accession No.	Protein Score	Protein Score C. I. %	Total Ion Score	Total Ion C. I. %
1	hypothetical protein PA2451	gi|15597647|ref|NP_251141.1|	261	100	178	100

Peptide Information

Calc. Mass	Obsrv. Mass	± da	± ppm	Start Seq.	End Seq.	Sequence	Ion Score	C. I. %	Modification	Rank	Result Type
905.4628	905.4553	-0.0075	-8	167	173	GYPLHYR					Mascot
1150.5375	1150.5311	-0.0064	-6	69	78	YALSEEPADR					Mascot
1665.917	1665.9174	0.0004	0	11	26	DVPTPTILDNLIAAGR	130	100			Mascot
1665.917	1665.9174	0.0004	0	11	26	DVPTPTILDNLIAAGR					Mascot
1768.0076	1768.0048	-0.0028	-2	27	43	IRPTLAVIVGNVDASSR	48	99.995			Mascot
1768.0076	1768.0048	-0.0028	-2	27	43	IRPTLAVIVGNVDASSR					Mascot
1817.885	1817.8768	-0.0082	-5	79	96	VLSGSSYGGLASGCIAYR			Carbamidomethyl (C)[14]		Mascot
2363.1448	2363.1506	0.0058	2	79	100	VLSGSSYGGLASGCIAYRYPER			Carbamidomethyl (C)[14]		Mascot
2675.3926	2675.3975	0.0049	2	133	157	LPLTFFLSSGLLEEPEADGILGSNR					Mascot
2797.3438	2797.3684	0.0246	9	46	68	ELPCNEAFADMLAHELLPWLQQR			Carbamidomethyl (C)[4], Oxidation (M)[11]		Mascot
3434.759	3434.7974	0.0384	11	126	157	QFAAGERLPLTFFLSSGLLEEPEADGILGSNR					Mascot

Rank	Protein Name	Accession No.	Protein Score	Protein Score C. I. %	Total Ion Score	Total Ion C. I. %
2	hypothetical protein PA2452	gi|15597648|ref|NP_251142.1|	239	100	51	99.997

Peptide Information

Calc. Mass	Obsrv. Mass	± da	± ppm	Start	End	Sequence	Ion	C. I. %	Modification	Rank	Result Type

				Seq.	Seq.		Score				
955.5068	955.4993	-0.0075	-8	178	185	QQRPGDVR					Mascot
1058.559	1058.5487	-0.0103	-10	83	92	QSLGLQAGER					Mascot
1220.6382	1220.6304	-0.0078	-6	197	207	RFEALAGSDVR					Mascot
1307.6703	1307.6655	-0.0048	-4	296	307	LGYSSQALANQR					Mascot
1325.7212	1325.7156	-0.0056	-4	186	196	LLWPTPEVNTR					Mascot
1424.738	1424.7297	-0.0083	-6	157	169	QGTPLVEPLDAER					Mascot
1478.8438	1478.8384	-0.0054	-4	240	253	LALQAAARPDPLSR					Mascot
1523.8904	1523.8827	-0.0077	-5	135	148	LQAELAEKRPGALK					Mascot
1918.9506	1918.9474	-0.0032	-2	219	235	VSYQLSADLPDLQQADR					Mascot
1992.0284	1992.0245	-0.0039	-2	97	114	LTFSADEDTALTLIPTQR					Mascot
2072.0911	2072.0952	0.0041	2	42	59	YLDLAPGELQLLDLEVDR					Mascot
2146.1504	2146.1562	0.0058	3	115	134	LTPQTPALDSPVPQSPTLQR	51	99.997			Mascot
2146.1504	2146.1562	0.0058	3	115	134	LTPQTPALDSPVPQSPTLQR					Mascot
2224.0994	2224.1021	0.0027	1	265	285	AEQASLVQLGGAPAETWDQPR					Mascot
2285.2136	2285.2166	0.003	1	42	61	YLDLAPGELQLLDLEVDRGR					Mascot
2346.2048	2346.2048	0	0	219	239	VSYQLSADLPDLQQADRGTLR					Mascot
2352.1943	2352.1992	0.0049	2	265	286	AEQASLVQLGGAPAETWDQPRK					Mascot
2356.2507	2356.2217	-0.029	-12	40	59	QRYLDLAPGELQLLDLEVDR					Mascot
2446.2937	2446.3115	0.0178	7	93	114	QGLRLTFSADEDTALTLIPTQR					Mascot
2661.3743	2661.3833	0.009	3	261	285	ALPRAEQASLVQLGGAPAETWDQPR					Mascot

Rank	Protein Name	Accession No.	Protein Score	Protein Score C. I. %	Total Ion Score	Total Ion C. I. %
1	hypothetical protein PA2699	gi|15597895|ref|NP_251389.1|	696	100	273	100

Peptide Information

Calc. Mass	Obsrv. Mass	± da	± ppm	Start Seq.	End Seq.	Sequence	Ion Score	C. I. %	Modification	Rank	Result Type
970.5104	970.5259	0.0155	16	103	110	TPSPQWVR					Mascot
1053.5687	1053.5808	0.0121	11	413	422	AAEATPPIQR					Mascot
1070.5953	1070.6108	0.0155	14	387	397	ALGGGIAIQDR					Mascot
1076.4796	1076.4948	0.0152	14	283	291	YGQGSDFFR	80	100			Mascot
1076.4796	1076.4948	0.0152	14	283	291	YGQGSDFFR					Mascot
1148.6058	1148.6224	0.0166	14	73	82	GGLNYNLELR					Mascot
1229.6484	1229.6639	0.0155	13	247	257	ELADQGQLTVR					Mascot
1251.6401	1251.6519	0.0118	9	351	360	MLDVFEKVDR					Mascot
1291.6277	1291.645	0.0173	13	340	350	LHATYDESISR					Mascot
1313.6848	1313.701	0.0162	12	83	94	WEGVPSLADALR					Mascot
1315.7329	1315.7286	-0.0043	-3	46	57	GSATQVIDLQKR					Mascot
1319.6954	1319.7118	0.0164	12	204	214	LPLEYQVNSTR					Mascot
1322.7467	1322.7402	-0.0065	-5	258	268	IAYNLFTQKPK					Mascot
1362.6147	1362.6289	0.0142	10	398	408	MAFQGEYFVDR					Mascot
1378.6096	1378.6229	0.0133	10	398	408	MAFQGEYFVDR			Oxidation (M)[1]		Mascot
1459.7726	1459.7859	0.0133	9	456	469	TVGGMALYPQGLPR					Mascot
1475.7675	1475.7788	0.0113	8	456	469	TVGGMALYPQGLPR			Oxidation (M)[5]		Mascot
1510.7324	1510.7429	0.0105	7	111	123	VVGGWNEFQFAEK					Mascot
1543.7686	1543.781	0.0124	8	32	45	FLAVGNDAEAMLHR					Mascot
1544.7737	1544.7866	0.0129	8	423	438	MLAEGVPVGAGTDATR					Mascot
1559.7634	1559.7792	0.0158	10	32	45	FLAVGNDAEAMLHR			Oxidation (M)[11]		Mascot
1601.8646	1601.8783	0.0137	9	201	214	GPKLPLEYQVNSTR					Mascot
1623.8013	1623.8119	0.0106	7	269	282	EELADFKNWTGSVK					Mascot
1666.8336	1666.8478	0.0142	9	111	124	VVGGWNEFQFAEKR					Mascot
1684.9493	1684.9592	0.0099	6	58	72	TVIPGLNDSHLHLIR	97	100			Mascot
1684.9493	1684.9592	0.0099	6	58	72	TVIPGLNDSHLHLIR					Mascot
1713.8959	1713.9044	0.0085	5	134	148	AAPDTPVFVLHLYDR	96	100			Mascot
1713.8959	1713.9044	0.0085	5	134	148	AAPDTPVFVLHLYDR					Mascot

Calc. Mass	Obsrv. Mass	± da	± ppm	Start Seq.	End Seq.	Sequence	Ion Score	C. I. %	Modification	Rank	Result Type
1781.8315	1781.8416	0.0101	6	398	412	MAFQGEYFVDRYGAK					Mascot
1797.8264	1797.8403	0.0139	8	398	412	MAFQGEYFVDRYGAK			Oxidation (M)[1]		Mascot
1841.0504	1841.0591	0.0087	5	57	72	RTVIPGLNDSHLHLIR					Mascot
1877.9293	1877.9364	0.0071	4	336	350	WPFRLHATYDESISR					Mascot
1898.9971	1899.0042	0.0071	4	158	175	VVGYTKDTPNPPGGEIVR					Mascot
1926.9603	1926.9766	0.0163	8	95	110	MLNNQAERTPSPQWVR					Mascot
2015.0021	2015.0044	0.0023	1	439	455	VSSYNPWTSLYWLVSGR					Mascot
2155.1433	2155.1372	-0.0061	-3	258	275	IAYNLFTQKPKEELADFK					Mascot
2224.0669	2224.0637	-0.0032	-1	470	489	ETALQLFTHGSAWFSSEQGK					Mascot
2352.1619	2352.1597	-0.0022	-1	470	490	ETALQLFTHGSAWFSSEQGKK					Mascot
2373.1663	2373.1582	-0.0081	-3	361	380	DIPFNGLPWFFDHAETITPR					Mascot
2443.2727	2443.2634	-0.0093	-4	73	94	GGLNYNLELRWEGVPSLADALR					Mascot
2570.2522	2570.2358	-0.0164	-6	223	246	LGLTSAIDAGGGFQNYPDDYQVIR					Mascot
2620.3474	2620.3252	-0.0222	-8	176	200	DSSGEPTGMLIARPNAMILYATLAK					Mascot
2636.3423	2636.3308	-0.0115	-4	176	200	DSSGEPTGMLIARPNAMILYATLAK			Oxidation (M)[9]		Mascot
2743.3628	2743.3464	-0.0164	-6	358	380	VDRDIPFNGLPWFFDHAETITPR					Mascot
2769.428	2769.3987	-0.0293	-11	125	148	MPTLEELNKAAPDTPVFVLHLYDR					Mascot
2834.5127	2834.4866	-0.0261	-9	530	556	VVYAAAEFDKLGPPAPPVLPDWSPVAK					Mascot

Rank	Protein Name	Accession No.	Protein Score	Protein Score C. I. %	Total Ion Score	Total Ion C. I. %
1	probable aminopeptidase (PaAP), PA2939	gi|15598135|ref|NP_251629.1|	421	100	252	100

Peptide Information

Calc. Mass	Obsrv. Mass	± da	± ppm	Start Seq.	End Seq.	Sequence	Ion Score	C. I. %	Modification	Rank	Result Type
945.4828	945.483	0.0002	0	402	408	LFEAYFR	59	100			Mascot
945.4828	945.483	0.0002	0	402	408	LFEAYFR					Mascot
1117.6146	1117.6102	-0.0044	-4	61	70	CLQASNVVKR					Mascot
1172.4678	1172.4622	-0.0056	-5	461	469	AYDECYHSK			Carbamidomethyl (C)[5]		Mascot
1215.6216	1215.6294	0.0078	6	501	512	VVDDEIAAAGQK					Mascot
1316.644	1316.641	-0.003	-2	74	85	LEDIASLNDGNR					Mascot
1349.8151	1349.8141	-0.001	-1	48	60	SPLLVSTPLGLPR	97	100			Mascot
1349.8151	1349.8141	-0.001	-1	48	60	SPLLVSTPLGLPR					Mascot
1366.6234	1366.6193	-0.0041	-3	411	422	GQQSEGTEIDFR					Mascot
1410.7223	1410.7157	-0.0066	-5	272	283	KTETYNVVAETR					Mascot
1540.7642	1540.798	0.0338	22	86	100	AAATPGYQASVDYVK					Mascot
1635.8085	1635.8046	-0.0039	-2	409	422	LRGQQSEGTEIDFR					Mascot
1643.8468	1643.8425	-0.0043	-3	110	123	VSVQPFPFTAYYPK					Mascot
1685.8817	1685.8792	-0.0025	-1	71	85	LQKLEDIASLNDGNR					Mascot
2063.0637	2063.0608	-0.0029	-1	106	123	AGYKVSVQPFPFTAYYPK					Mascot
2139.1082	2139.1084	0.0002	0	86	105	AAATPGYQASVDYVKQTLQK					Mascot
2246.0796	2246.0828	0.0032	1	203	225	AENAAAAGAAGVIIFNQGNTDDR					Mascot
2374.1746	2374.1775	0.0029	1	203	226	AENAAAAGAAGVIIFNQGNTDDRK	96	100			Mascot
2374.1746	2374.1775	0.0029	1	203	226	AENAAAAGAAGVIIFNQGNTDDRK					Mascot
2889.3843	2889.397	0.0127	4	334	359	FAWWGAEEAGLVGSTHYVQNLAPEEK					Mascot
3153.4653	3153.5647	0.0994	32	195	225	GTCNFEQKAENAAAAGAAGVIIFNQGNTDDR					Mascot
3210.4868	3210.5583	0.0715	22	195	225	GTCNFEQKAENAAAAGAAGVIIFNQGNTDDR			Carbamidomethyl (C)[3]		Mascot
3312.5693	3312.5911	0.0218	7	423	453	SDYAEFFNSGIAFGGLFTGAEGLKTEEQAQK					Mascot
3606.7744	3606.7908	0.0164	5	159	194	VVPVDLSLGAGNTSTSGCEAEDFANFPAGSIALIQR					Mascot
3663.7959	3663.8267	0.0308	8	159	194	VVPVDLSLGAGNTSTSGCEAEDFANFPAGSIALIQR			Carbamidomethyl (C)[18]		Mascot

Rank	Protein Name	Accession No.	Protein Score	Protein Score C. I. %	Total Ion Score	Total Ion C. I. %

Rank	Protein Name	Accession No.	Protein Score	Protein Score C. I. %	Total Ion Score	Total Ion C. I. %
1	probable binding protein component of ABC PA3190	gi|15598386|ref|NP_251880.1|	302	100	193	100

Peptide Information

Calc. Mass	Obsrv. Mass	± da	± ppm	Start Seq.	End Seq.	Sequence	Ion Score	C. I. %	Modification	Rank	Result Type
1223.5547	1223.5497	-0.005	-4	278	288	AGMQIMGDWAK			Oxidation (M)[3]		Mascot
1562.756	1562.7577	0.0017	1	313	325	AFDYNIDSLVMFK					Mascot
1578.7509	1578.7454	-0.0055	-3	313	325	AFDYNIDSLVMFK			Oxidation (M)[11]		Mascot
1617.802	1617.7975	-0.0045	-3	147	160	YDGDYVAVPINVHR					Mascot
1649.9049	1649.9038	-0.0011	-1	161	173	VNWLYINPEVFKK					Mascot
1751.8962	1751.8958	-0.0004	0	341	355	SVLDPSFQKDFNLNK					Mascot
1793.9829	1793.9391	-0.0438	-24	236	252	ATLTGAQMVEVFAALKK			Oxidation (M)[8]		Mascot
1834.9156	1834.9113	-0.0043	-2	297	312	VPGKDYQCLPFPGTQK			Carbamidomethyl (C)[8]		Mascot
2058.1118	2058.1082	-0.0036	-2	174	193	AGATPPTTLDELFVAADKLK					Mascot
2203.0964	2203.0918	-0.0046	-2	72	93	GHTWKDFAVAGGGGEAAMTVLK					Mascot
2219.0913	2219.0847	-0.0066	-3	72	93	GHTWKDFAVAGGGGEAAMTVLK			Oxidation (M)[18]		Mascot
2353.124	2353.1233	-0.0007	0	255	277	GYVDADAAGREWSAATAMVINGK					Mascot
2416.2441	2416.2336	-0.0105	-4	140	160	QVAQIMKYDGDYVAVPINVHR	111	100			Mascot
2416.2441	2416.2336	-0.0105	-4	140	160	QVAQIMKYDGDYVAVPINVHR					Mascot
2432.239	2432.2368	-0.0022	-1	140	160	QVAQIMKYDGDYVAVPINVHR			Oxidation (M)[6]		Mascot
2483.2849	2483.2788	-0.0061	-2	229	251	AFVEQDKATLTGAQMVEVFAALK			Oxidation (M)[15]		Mascot
2524.1628	2524.1667	0.0039	2	356	378	GSIPVRLDADMAPFDSCAQQSMK			Carbamidomethyl (C)[17]		Mascot
2540.1577	2540.1519	-0.0058	-2	356	378	GSIPVRLDADMAPFDSCAQQSMK			Carbamidomethyl (C)[17], Oxidation (M)[11]		Mascot
2870.447	2870.4485	0.0015	1	194	220	AAGFTPLAHGSQPWQDGTVFENLVLSK	82	100			Mascot
2870.447	2870.4485	0.0015	1	194	220	AAGFTPLAHGSQPWQDGTVFENLVLSK					Mascot

Rank	Protein Name	Accession No.	Protein Score	Protein Score C. I. %	Total Ion Score	Total Ion C. I. %
1	hypothetical protein PA3250	gi|15598446|ref|NP_251940.1|	289	100	173	100

Peptide Information

Calc. Mass	Obsrv. Mass	± da	± ppm	Start Seq.	End Seq.	Sequence	Ion Score	C. I. %	Modification	Rank	Result Type
1006.4952	1006.5102	0.015	15	331	338	AWEETSKR					Mascot
1249.6938	1249.7012	0.0074	6	193	203	NIKPGLEFFGK	86	100			Mascot
1249.6938	1249.7012	0.0074	6	193	203	NIKPGLEFFGK					Mascot
1274.674	1274.6794	0.0054	4	312	322	AQLLPNEQYAK					Mascot
1465.7434	1465.7479	0.0045	3	150	162	DVPHSWADLLQGK					Mascot
1568.7955	1568.8002	0.0047	3	280	293	EYIFSDAGQINLAK					Mascot
1909.0177	1909.0216	0.0039	2	277	293	LAREYIFSDAGQINLAK					Mascot
1934.0494	1934.0537	0.0043	2	146	162	QLVKDVPHSWADLLQGK					Mascot
1939.9548	1939.9651	0.0103	5	223	239	GEVEVGIVWDFNGLSYR					Mascot
1952.0559	1952.0594	0.0035	2	294	311	GNARPIRAESLTLPDDVK	50	99.995			Mascot
1952.0559	1952.0594	0.0035	2	294	311	GNARPIRAESLTLPDDVK					Mascot
2220.1084	2220.1042	-0.0042	-2	126	145	DQDGHWALAYTGTIAFIVNK					Mascot
2443.2715	2443.2573	-0.0142	-6	301	322	AESLTLPDDVKAQLLPNEQYAK					Mascot
2873.5002	2873.4714	-0.0288	-10	163	192	YKVTIGDVSAAAQAVNGVLAAAIANGGDEK					Mascot
3119.626	3119.5706	-0.0554	-18	240	267	QQIDPERFEVLIPSDGSVISGYTTIINK	37	99.902			Mascot
3119.626	3119.5706	-0.0554	-18	240	267	QQIDPERFEVLIPSDGSVISGYTTIINK					Mascot

Rank	Protein Name	Accession No.	Protein Score	Protein Score	Total Ion Score	Total Ion C. I. %

				C. I. %		
1	Pyrophosphate-specific outer membrane porin PA3280 (OprO)	gi|15598476|ref|NP_251970.1|	426	100	205	100

Peptide Information

Calc. Mass	Obsrv. Mass	± da	± ppm	Start Seq.	End Seq.	Sequence	Ion Score	C. I. %	Modification	Rank	Result Type
1067.5044	1067.5204	0.016	15	152	160	FDPDFGLEK					Mascot
1154.4961	1154.5205	0.0244	21	249	258	DLDDTAFDSR					Mascot
1199.5439	1199.5695	0.0256	21	88	98	NGNTADAAYFR					Mascot
1269.6262	1269.6462	0.02	16	370	379	EIGAWEVFYR	12	53.818			Mascot
1269.6262	1269.6462	0.02	16	370	379	EIGAWEVFYR					Mascot
1355.645	1355.6631	0.0181	13	88	99	NGNTADAAYFRR					Mascot
1427.6953	1427.7162	0.0209	15	149	160	FGRFDPDFGLEK					Mascot
1598.8213	1598.84	0.0187	12	100	113	AFIELGGTAYKDWK					Mascot
1628.8167	1628.8315	0.0148	9	55	69	GGLEVATTDKEFSFK					Mascot
1692.8228	1692.8436	0.0208	12	338	352	GYYAQLAYTLTGESR	120	100			Mascot
1692.8228	1692.8436	0.0208	12	338	352	GYYAQLAYTLTGESR					Mascot
1699.8551	1699.87	0.0149	9	406	420	AHNLGVNWYVNDAVK					Mascot
1709.7362	1709.7882	0.052	30	433	448	ITNNNGDDDGDGFVTR					Mascot
1710.8123	1710.8287	0.0164	10	74	87	LQADYSRFDGFYTK	74	100			Mascot
1710.8123	1710.8287	0.0164	10	74	87	LQADYSRFDGFYTK					Mascot
1815.7781	1815.7932	0.0151	8	211	226	DADDSDGDSVKQFNFR					Mascot
1891.9548	1891.972	0.0172	9	336	352	AKGYYAQLAYTLTGESR					Mascot
1898.9871	1898.9988	0.0117	6	404	420	AKAHNLGVNWYVNDAVK					Mascot
1902.9385	1902.9626	0.0241	13	370	384	EIGAWEVFYRYDNIK					Mascot
2037.9724	2037.9788	0.0064	3	380	397	YDNIKVEDDNVVADTATR					Mascot
2053.9058	2053.9211	0.0153	7	430	448	TDKITNNNGDDDGDGFVTR					Mascot
2314.1501	2314.1533	0.0032	1	361	379	FDSVKPENKEIGAWEVFYR					Mascot
2401.187	2401.1848	-0.0022	-1	227	248	GVFAPMHEAGNVLHVGVNYAYR					Mascot
2417.1819	2417.1873	0.0054	2	227	248	GVFAPMHEAGNVLHVGVNYAYR			Oxidation (M)[6]		Mascot
2432.272	2432.2393	-0.0327	-13	406	427	AHNLGVNWYVNDAVKISAAYVK					Mascot

Rank	Protein Name	Accession No.	Protein Score	Protein Score C. I. %	Total Ion Score	Total Ion C. I. %
1	alkaline phosphatase (PhoA), PA3296	gi|15598492|ref|NP_251986.1|	600	100	366	100

Peptide Information

Calc. Mass	Obsrv. Mass	± da	± ppm	Start Seq.	End Seq.	Sequence	Ion Score	C. I. %	Modification	Rank	Result Type
1038.485	1038.4736	-0.0114	-11	40	49	GELSEYGGAR					Mascot
1300.7219	1300.7124	-0.0095	-7	50	60	RVEQDLTQALK					Mascot
1393.6819	1393.6768	-0.0051	-4	37	49	QARGELSEYGGAR					Mascot
1398.795	1398.7842	-0.0108	-8	262	273	IVENLDELKAVR					Mascot
1609.8883	1609.8739	-0.0144	-9	279	293	QPLIGLFAPGNMPVR					Mascot
1625.8832	1625.8708	-0.0124	-8	279	293	QPLIGLFAPGNMPVR			Oxidation (M)[12]		Mascot
1792.8726	1792.8683	-0.0043	-2	141	156	SYNGAIGVDIHEQPHR					Mascot
1860.9813	1860.9731	-0.0082	-4	224	242	TRPDVVLGGGAATFAETAK	86	100			Mascot
1860.9813	1860.9731	-0.0082	-4	224	242	TRPDVVLGGGAATFAETAK					Mascot
1973.0371	1973.0762	0.0391	20	70	88	NVILLIGDGMGDSEITVAR					Mascot
1989.0321	1989.0194	-0.0127	-6	70	88	NVILLIGDGMGDSEITVAR			Oxidation (M)[10]		Mascot
2014.0393	2014.0284	-0.0109	-5	100	117	GIDALPLTGQYTHYSLHK					Mascot
2051.1218	2051.1111	-0.0107	-5	275	293	ANQKQPLIGLFAPGNMPVR					Mascot
2067.1167	2067.106	-0.0107	-5	275	293	ANQKQPLIGLFAPGNMPVR			Oxidation (M)[16]		Mascot
2145.1411	2145.1318	-0.0093	-4	224	245	TRPDVVLGGGAATFAETAKAGR					Mascot

Calc. Mass	Obsrv. Mass	± da	± ppm	Start Seq.	End Seq.	Sequence	Ion Score	C. I. %	Modification	Rank	Result Type
2172.1692	2172.1602	-0.009	-4	68	88	AKNVILLIGDGMGDSEITVAR					Mascot
2188.1641	2188.1479	-0.0162	-7	68	88	AKNVILLIGDGMGDSEITVAR			Oxidation (M)[12]		Mascot
2478.2947	2478.2908	-0.0039	-2	168	192	ATGNVSTAELQDATPAALLAHVTAR	147	100			Mascot
2478.2947	2478.2908	-0.0039	-2	168	192	ATGNVSTAELQDATPAALLAHVTAR					Mascot
2574.3423	2574.3413	-0.001	0	141	163	SYNGAIGVDIHEQPHRNLLELAK					Mascot
2606.3896	2606.3855	-0.0041	-2	168	193	ATGNVSTAELQDATPAALLAHVTARK					Mascot
2625.2515	2625.2512	-0.0003	0	294	317	WLGPTATYHGNLNQPAVSCEANPK			Carbamidomethyl (C)[19]		Mascot
2647.2229	2647.219	-0.0039	-1	420	444	DGAPLAISYGNSEESSQEHTGTQLR	132	100			Mascot
2647.2229	2647.219	-0.0039	-1	420	444	DGAPLAISYGNSEESSQEHTGTQLR					Mascot
2683.3726	2683.3621	-0.0105	-4	445	469	IAAYGPQAANVTGLTDQTDLFFTIR					Mascot
2694.3674	2694.3572	-0.0102	-4	93	117	GAGGYFKGIDALPLTGQYTHYSLHK					Mascot
2781.3525	2781.3518	-0.0007	0	294	318	WLGPTATYHGNLNQPAVSCEANPKR			Carbamidomethyl (C)[19]		Mascot
2839.4736	2839.4641	-0.0095	-3	445	470	IAAYGPQAANVTGLTDQTDLFFTIRR					Mascot
2890.5381	2890.5298	-0.0083	-3	164	192	LNGKATGNVSTAELQDATPAALLAHVTAR					Mascot
3695.949	3695.9556	0.0066	2	384	419	ADGETLVIVTADHAHSSQIIPPETAAPGLTQLLTTK					Mascot

Rank	Protein Name	Accession No.	Protein Score	Protein Score C. I. %	Total Ion Score	Total Ion C. I. %
1	heme acquisition protein HasAp PA3407	gi|15598603|ref|NP_252097.1|	73	99.973	61	100

Peptide Information

Calc. Mass	Obsrv. Mass	± da	± ppm	Start Seq.	End Seq.	Sequence	Ion Score	C. I. %	Modification	Rank	Result Type
2281.1743	2281.175	0.0007	0	136	157	VVYGLMSGDSSALQGQIDALLK			Oxidation (M)[6]		Mascot
2918.4927	2918.5059	0.0132	5	130	157	DGTVHKVVYGLMSGDSSALQGQIDALLK	61	100	Oxidation (M)[12]		Mascot
2918.4927	2918.5059	0.0132	5	130	157	DGTVHKVVYGLMSGDSSALQGQIDALLK			Oxidation (M)[12]		Mascot

Rank	Protein Name	Accession No.	Protein Score	Protein Score C. I. %	Total Ion Score	Total Ion C. I. %
1	elastase LasB PA3724	gi|15598919|ref|NP_252413.1|	294	100	136	100

Peptide Information

Calc. Mass	Obsrv. Mass	± da	± ppm	Start Seq.	End Seq.	Sequence	Ion Score	C. I. %	Modification	Rank	Result Type
1101.5034	1101.4976	-0.0058	-5	253	261	FACPTNTYK			Carbamidomethyl (C)[3]		Mascot
1166.6139	1166.6122	-0.0017	-1	297	305	LYMKVHYGR					Mascot
1167.5792	1167.5913	0.0121	10	444	453	AFEVFVDANR					Mascot
1224.5426	1224.5369	-0.0057	-5	396	405	YMDQPSRDGR					Mascot
1240.5375	1240.5328	-0.0047	-4	396	405	YMDQPSRDGR			Oxidation (M)[2]		Mascot
1288.6321	1288.6241	-0.008	-6	286	296	DWFGTSPLTHK					Mascot
1295.6742	1295.6707	-0.0035	-3	443	453	KAFEVFVDANR					Mascot
1382.7314	1382.7301	-0.0013	-1	377	388	GKNDFLIGYDIK					Mascot
1552.7462	1552.7443	-0.0019	-1	472	485	SAQNRNYSAADVTR					Mascot
1610.7961	1610.7985	0.0024	1	429	442	AFYLLANSPGWDTR	89	100			Mascot
1610.7961	1610.7985	0.0024	1	429	442	AFYLLANSPGWDTR					Mascot
1720.8806	1720.8822	0.0016	1	283	296	LYRDWFGTSPLTHK					Mascot
1732.8177	1732.8171	-0.0006	0	212	226	YTYGSDYGPLIVNDR					Mascot
1738.8911	1738.8894	-0.0017	-1	429	443	AFYLLANSPGWDTRK	29	99.425			Mascot
1738.8911	1738.8894	-0.0017	-1	429	443	AFYLLANSPGWDTRK					Mascot
2031.0182	2031.0184	0.0002	0	209	226	IGKYTYGSDYGPLIVNDR					Mascot
2082.9338	2082.9358	0.002	1	454	471	YYWTATSNYNSGACGVIR			Carbamidomethyl (C)[14]		Mascot

2267.1243	2267.126	0.0017	1	262	282	QVNGAYSPLNDAHFFGGVVFK					Mascot
2455.9951	2456.0039	0.0088	4	354	376	GQSGGMNEAFSDMAGEAAEFYMR					Mascot
2471.99	2472.0037	0.0137	6	354	376	GQSGGMNEAFSDMAGEAAEFYMR			Oxidation (M)[6]		Mascot
2487.9849	2488.0073	0.0224	9	354	376	GQSGGMNEAFSDMAGEAAEFYMR			Oxidation (M)[6,13]		Mascot
2503.9797	2504.0146	0.0349	14	354	376	GQSGGMNEAFSDMAGEAAEFYMR			Oxidation (M)[6,13,22]		Mascot
2596.1812	2596.1934	0.0122	5	406	428	SIDNASQYYNGIDVHHSSGVYNR	18	93.461			Mascot
2596.1812	2596.1934	0.0122	5	406	428	SIDNASQYYNGIDVHHSSGVYNR					Mascot
2699.3728	2699.3735	0.0007	0	262	285	QVNGAYSPLNDAHFFGGVVFKLYR					Mascot
3231.4951	3231.5164	0.0213	7	444	471	AFEVFVDANRYYWTATSNYNSGACGVIR			Carbamidomethyl (C)[24]		Mascot

Rank	Protein Name	Accession No.	Protein Score	Protein Score C. I. %	Total Ion Score	Total Ion C. I. %
1	hypothetical protein PA3734	gi|15598929|ref|NP_252423.1|	405	100	265	100

Peptide Information

Calc. Mass	Obsrv. Mass	± da	± ppm	Start Seq.	End Seq.	Sequence	Ion Score	C. I. %	Modification	Rank	Result Type
985.604	985.5997	-0.0043	-4	134	142	IVLLDGSIR					Mascot
1210.5891	1210.5864	-0.0027	-2	368	376	DYFVGRYYK					Mascot
1307.6815	1307.6793	-0.0022	-2	226	237	LDRDPASALHGR					Mascot
1388.7169	1388.7252	0.0083	6	377	388	LASDIQFNDPLR	83	100			Mascot
1388.7169	1388.7252	0.0083	6	377	388	LASDIQFNDPLR					Mascot
1411.6713	1411.6691	-0.0022	-2	362	373	NDAQARDYFVGR					Mascot
1787.0537	1787.0541	0.0004	0	126	142	GFVGTIARIVLLDGSIR					Mascot
1832.0891	1832.0846	-0.0045	-2	275	293	LVGALPIEPGPLAIGSTVK					Mascot
1842.9385	1842.938	-0.0005	0	374	388	YYKLASDIQFNDPLR					Mascot
1999.0032	1999.0055	0.0023	1	323	341	DVPAWGATATTATHFSPLR					Mascot
2073.2681	2073.2651	-0.003	-1	273	293	LKLVGALPIEPGPLAIGSTVK					Mascot
2111.1721	2111.1716	-0.0005	0	377	394	LASDIQFNDPLRLLRPSR					Mascot
2970.4744	2970.4888	0.0144	5	342	367	QIEYNEFAGISVAWVLYQGKNDAQAR					Mascot
2975.5908	2975.6013	0.0105	4	243	272	TLVAGHSAGGQATLQSASLSAQALQLIEPR	183	100			Mascot
2975.5908	2975.6013	0.0105	4	243	272	TLVAGHSAGGQATLQSASLSAQALQLIEPR					Mascot
3112.4243	3112.4592	0.0349	11	143	171	CNEAFPYGFASPISTSVYYPADIAASDAK			Carbamidomethyl (C)[1]		Mascot
3216.7698	3216.7896	0.0198	6	243	274	TLVAGHSAGGQATLQSASLSAQALQLIEPRLK					Mascot
3551.8564	3551.8811	0.0247	7	238	272	ADFSRTLVAGHSAGGQATLQSASLSAQALQLIEPR					Mascot

Rank	Protein Name	Accession No.	Protein Score	Protein Score C. I. %	Total Ion Score	Total Ion C. I. %
1	hypothetical protein PA3910	gi|15599105|ref|NP_252599.1|	608	100	279	100

Peptide Information

Calc. Mass	Obsrv. Mass	± da	± ppm	Start Seq.	End Seq.	Sequence	Ion Score	C. I. %	Modification	Rank	Result Type
977.5162	977.507	-0.0092	-9	73	80	SVFSEPRR					Mascot
1049.5084	1049.4977	-0.0107	-10	65	72	MIVEWDTR					Mascot
1065.5034	1065.4902	-0.0132	-12	65	72	MIVEWDTR			Oxidation (M)[1]		Mascot
1071.6045	1071.5951	-0.0094	-9	394	402	ELEIADLLR					Mascot
1100.6211	1100.6129	-0.0082	-7	338	345	EQLQWLKR					Mascot
1135.5742	1135.5668	-0.0074	-7	224	232	YNLLDDNLR					Mascot
1207.647	1207.6376	-0.0094	-8	277	286	QAFLEYAPLR	82	100			Mascot
1207.647	1207.6376	-0.0094	-8	277	286	QAFLEYAPLR					Mascot

1246.6538	1246.6488	-0.005	-4	192	201	IETESGRIWR					Mascot
1266.6477	1266.6432	-0.0045	-4	100	110	GLPADQSIFYR	94	100			Mascot
1266.6477	1266.6432	-0.0045	-4	100	110	GLPADQSIFYR					Mascot
1291.6753	1291.6696	-0.0057	-4	224	233	YNLLDDNLRR					Mascot
1390.7689	1390.7679	-0.001	-1	263	274	YQVKDIDVLAAR					Mascot
1393.6931	1393.6932	0.0001	0	315	327	SYRGGNSANLQAR					Mascot
1434.7852	1434.7836	-0.0016	-1	275	286	ARQAFLEYAPLR					Mascot
1446.6819	1446.683	0.0011	1	39	52	LTDGVQSGDVQGDR					Mascot
1499.7754	1499.7726	-0.0028	-2	118	130	DGSLSKPWFGHLR	103	100			Mascot
1499.7754	1499.7726	-0.0028	-2	118	130	DGSLSKPWFGHLR					Mascot
1521.8173	1521.809	-0.0083	-5	100	112	GLPADQSIFYRVR					Mascot
1569.7656	1569.7644	-0.0012	-1	211	223	VAETLDEFRGNYR					Mascot
1707.9138	1707.9144	0.0006	0	300	314	VAYGPLLDVFVLDMR					Mascot
1723.9087	1723.9058	-0.0029	-2	300	314	VAYGPLLDVFVLDMR			Oxidation (M)[14]		Mascot
1734.9021	1734.9014	-0.0007	0	81	95	LVSPVTDERLDYTAR					Mascot
1763.9438	1763.9458	0.002	1	96	110	IDLRGLPADQSIFYR					Mascot
1836.0088	1836.0098	0.001	1	299	314	KVAYGPLLDVFVLDMR					Mascot
1852.0037	1851.9736	-0.0301	-16	299	314	KVAYGPLLDVFVLDMR			Oxidation (M)[15]		Mascot
1862.976	1862.9796	0.0036	2	329	344	SAATDFLGREQLQWLK					Mascot
2118.0515	2118.0583	0.0068	3	113	130	FEDARDGSLSKPWFGHLR					Mascot
2159.084	2159.093	0.009	4	39	58	LTDGVQSGDVQGDRALVWSR					Mascot
2183.0947	2183.0962	0.0015	1	356	376	VIAADMPIGLCVPDGKDAQGR			Carbamidomethyl (C)[11]		Mascot
2199.0896	2199.0916	0.002	1	356	376	VIAADMPIGLCVPDGKDAQGR			Carbamidomethyl (C)[11], Oxidation (M)[6]		Mascot
2371.0593	2371.0564	-0.0029	-1	141	162	FVWSGDTCGQGFGINPDIGGMR			Carbamidomethyl (C)[8]		Mascot
2387.0542	2387.0542	0	0	141	162	FVWSGDTCGQGFGINPDIGGMR			Carbamidomethyl (C)[8], Oxidation (M)[21]		Mascot
2527.1572	2527.1528	-0.0044	-2	411	431	NTVWLTADVHYCAAHHYSPER			Carbamidomethyl (C)[12]		Mascot
2571.2937	2571.2878	-0.0059	-2	497	520	ALTVTLRDLDGEPVFSQELQPDGA					Mascot
2857.3792	2857.3657	-0.0135	-5	471	496	APLVQNSSPFAGYQFFGEVEIDAQSR					Mascot
2910.3853	2910.374	-0.0113	-4	407	431	AGVRNTVWLTADVHYCAAHHYSPER			Carbamidomethyl (C)[16]		Mascot
2919.2969	2919.2866	-0.0103	-4	234	257	FNAEVPQIWQWDDHETTNNWSSSK					Mascot
3075.3979	3075.3748	-0.0231	-8	233	257	RFNAEVPQIWQWDDHETTNNWSSSK					Mascot

Rank	Protein Name	Accession No.	Protein Score	Protein Score C. I. %	Total Ion Score	Total Ion C. I. %
**1**	**hypothetical protein PA4140**	**gi|15599335|ref|NP_252829.1|**	**306**	**100**	**97**	**100**

Peptide Information

Calc. Mass	Obsrv. Mass	± da	± ppm	Start Seq.	End Seq.	Sequence	Ion Score	C. I. %	Modification	Rank	Result Type
985.4989	985.493	-0.0059	-6	301	308	TFDSFLQK					Mascot
1164.6372	1164.6361	-0.0011	-1	413	423	VTANGYAVLTR					Mascot
1285.6536	1285.655	0.0014	1	301	311	TFDSFLQKSGR					Mascot
1368.7092	1368.7115	0.0023	2	117	128	GMGHNWSPLLLK			Oxidation (M)[2]		Mascot
1404.7556	1404.7563	0.0007	0	586	597	LFSSPLLDRLMP			Oxidation (M)[11]		Mascot
1431.8569	1431.856	-0.0009	-1	401	412	DLLLYIKPSTLR					Mascot
1449.8424	1449.8431	0.0007	0	137	148	IVLVETSRYLTR					Mascot
1544.7791	1544.7045	-0.0746	-48	327	339	VWTPTPRCPFGAR			Carbamidomethyl (C)[8]		Mascot
1545.7292	1545.7181	-0.0111	-7	563	576	QGLVADNDWDSAVR					Mascot
1554.7482	1554.7386	-0.0096	-6	447	460	ANGHYPMNGPVEIR					Mascot
1567.7686	1567.7694	0.0008	1	249	262	DDPACAPFLVHLGR			Carbamidomethyl (C)[5]		Mascot
1570.743	1570.748	0.005	3	447	460	ANGHYPMNGPVEIR			Oxidation (M)[7]		Mascot
1616.9119	1616.9119	0	0	263	277	AFIVEATLQAGVNKR	97	100			Mascot
1616.9119	1616.9119	0	0	263	277	AFIVEATLQAGVNKR					Mascot
1723.8698	1723.8708	0.001	1	248	262	RDDPACAPFLVHLGR			Carbamidomethyl (C)[6]		Mascot
1779.87	1779.866	-0.004	-2	340	355	AVNGPFNYPFSDNIPK					Mascot
1805.881	1805.8865	0.0055	3	129	144	GGENCESRIVLVETSR			Carbamidomethyl (C)[5]		Mascot
2105.0449	2105.0642	0.0193	9	95	112	TNEEVLAVVNWAWQNGFK					Mascot

Calc. Mass	Obsrv. Mass	± da	± ppm	Start Seq.	End Seq.	Sequence	Ion Score	C. I. %	Modification	Rank	Result Type
2108.0884	2108.0747	-0.0137	-6	577	594	QLNEADPHRLFSSPLLDR					Mascot
2109.0474	2109.0691	0.0217	10	430	446	VINEFYLQYQTMVAAYR					Mascot
2125.0422	2125.0449	0.0027	1	430	446	VINEFYLQYQTMVAAYR			Oxidation (M)[12]		Mascot
2172.98	2172.9722	-0.0078	-4	280	300	CQSYVNIPASEMFAAAGSGGR			Carbamidomethyl (C)[1]		Mascot
2188.9749	2188.9736	-0.0013	-1	280	300	CQSYVNIPASEMFAAAGSGGR			Carbamidomethyl (C)[1], Oxidation (M)[12]		Mascot
2244.2598	2244.249	-0.0108	-5	356	377	ALSDLLAAINTGHPELTPLLGK					Mascot
2623.2974	2623.3325	0.0351	13	426	446	DVQRVINEFYLQYQTMVAAYR			Oxidation (M)[16]		Mascot
2681.3967	2681.384	-0.0127	-5	149	173	VRIDAQGEFGLFSAQTGVTMEALLK					Mascot
2697.3916	2697.3718	-0.0198	-7	149	173	VRIDAQGEFGLFSAQTGVTMEALLK			Oxidation (M)[20]		Mascot
3244.696	3244.708	0.012	4	180	213	LGFVATPAPGDLTLGGVLAIDGHGTGIPAQGESR					Mascot
3471.8594	3471.8691	0.0097	3	178	213	VKLGFVATPAPGDLTLGGVLAIDGHGTGIPAQGESR					Mascot

Rank	Protein Name	Accession No.	Protein Score	Protein Score C. I. %	Total Ion Score	Total Ion C. I. %
1	**PvdS-regulated endoprotease, lysyl class (PrpL) PA4175**	**gi|15599370|ref|NP_252864.1|**	**454**	**100**	**320**	**100**

Peptide Information

Calc. Mass	Obsrv. Mass	± da	± ppm	Start Seq.	End Seq.	Sequence	Ion Score	C. I. %	Modification	Rank	Result Type
1100.6674	1100.6681	0.0007	1	333	341	DTLLLELKR					Mascot
1727.7805	1727.781	0.0005	0	426	442	GGLYGGPSYCGAPTSQR	135	100	Carbamidomethyl (C)[10]		Mascot
1727.7805	1727.781	0.0005	0	426	442	GGLYGGPSYCGAPTSQR			Carbamidomethyl (C)[10]		Mascot
1884.8398	1884.8416	0.0018	1	443	458	NDYFSDFSGVYSQISR	163	100			Mascot
1884.8398	1884.8416	0.0018	1	443	458	NDYFSDFSGVYSQISR					Mascot
2196.0679	2196.0652	-0.0027	-1	376	396	YSQGNVSAVGVTYDGHTALTR					Mascot
2231.9292	2231.927	-0.0022	-1	216	237	DGFGASGSCEVDAVCATQSGTR			Carbamidomethyl (C)[9,15]		Mascot
2324.1628	2324.1614	-0.0014	-1	375	396	KYSQGNVSAVGVTYDGHTALTR					Mascot
2679.1521	2679.1643	0.0122	5	212	237	AGYRDGFGASGSCEVDAVCATQSGTR	22	98.75	Carbamidomethyl (C)[13,19]		Mascot
2679.1521	2679.1643	0.0122	5	212	237	AGYRDGFGASGSCEVDAVCATQSGTR			Carbamidomethyl (C)[13,19]		Mascot
2691.2388	2691.2615	0.0227	8	249	274	MVFTSSADGGSYICTGTLLNNGNSPK			Carbamidomethyl (C)[14]		Mascot
2707.2339	2707.241	0.0071	3	249	274	MVFTSSADGGSYICTGTLLNNGNSPK			Carbamidomethyl (C)[14], Oxidation (M)[1]		Mascot
2847.3401	2847.3464	0.0063	2	249	275	MVFTSSADGGSYICTGTLLNNGNSPKR			Carbamidomethyl (C)[14]		Mascot
2863.335	2863.3408	0.0058	2	249	275	MVFTSSADGGSYICTGTLLNNGNSPKR			Carbamidomethyl (C)[14], Oxidation (M)[1]		Mascot
2864.406	2864.3889	-0.0171	-6	397	425	VDWPSAVVEGGSSGSGLLTVAGDGSYQLR					Mascot
3083.5234	3083.5432	0.0198	6	342	370	TPPAGVFYQGWSATPIANGSLGHDIHHPR					Mascot
3239.6245	3239.6265	0.002	1	341	370	RTPPAGVFYQGWSATPIANGSLGHDIHHPR					Mascot
3307.459	3307.4788	0.0198	6	216	248	DGFGASGSCEVDAVCATQSGTRAYDNATAAVAK			Carbamidomethyl (C)[9,15]		Mascot
3454.7039	3454.7168	0.0129	4	342	374	TPPAGVFYQGWSATPIANGSLGHDIHHPRGDAK					Mascot
3593.6025	3593.6414	0.0389	11	426	458	GGLYGGPSYCGAPTSQRNDYFSDFSGVYSQISR			Carbamidomethyl (C)[10]		Mascot

Rank	Protein Name	Accession No.	Protein Score	Protein Score C. I. %	Total Ion Score	Total Ion C. I. %
1	**hypothetical protein PA4625**	**gi|15599821|ref|NP_253315.1|**	**232**	**100**	**147**	**100**

Peptide Information

Calc. Mass	Obsrv. Mass	± da	± ppm	Start Seq.	End Seq.	Sequence	Ion Score	C. I. %	Modification	Rank	Result Type
1027.6146	1027.6232	0.0086	8	1797	1806	ALLNVIADAK					Mascot
1061.599	1061.6053	0.0063	6	1958	1967	ALLNVFADAK					Mascot
1127.5116	1127.5271	0.0155	14	792	801	EGAFNSFAER					Mascot

1281.662	1281.6697	0.0077	6	782	791	VDTRLDLFMR			Oxidation (M)[9]	Mascot
1371.6975	1371.717	0.0195	14	1987	2001	NGDSAGQVLAGGLGR				Mascot
1431.7186	1431.7345	0.0159	11	1907	1921	NGDSAGSILTGGLNR				Mascot
1445.7343	1445.7533	0.019	13	1181	1195	NGDTAGSILTGGLNR				Mascot
1470.7659	1470.7806	0.0147	10	1745	1760	NGDTAGAVLNGGGLVR				Mascot
1488.7401	1488.7615	0.0214	14	1826	1841	NGDTAGAVLNGGSLSR				Mascot
1530.7982	1530.8065	0.0083	5	1019	1034	RGDTAGAVLNGGSLSR				Mascot
1654.9235	1654.9371	0.0136	8	568	584	ISQQAGTALIAANLAGR	147	100		Mascot
1654.9235	1654.9371	0.0136	8	568	584	ISQQAGTALIAANLAGR				Mascot
1684.9229	1684.9281	0.0052	3	487	503	LSVEAPLITSNLGGASR				Mascot
1812.9014	1812.9064	0.005	3	1890	1906	VYGDADPSLTYQVSGLK				Mascot
1841.9828	1841.9851	0.0023	1	392	411	ILARGGAQGGNGGLVETSGK				Mascot
1918.9116	1918.9111	-0.0005	0	786	801	LDLFMREGAFNSFAER			Oxidation (M)[5]	Mascot
1983.0869	1983.0927	0.0058	3	901	919	QDGRLTVTPAQLIVSADAK				Mascot
1995.0546	1995.0557	0.0011	1	1002	1019	VYGDLDPALTYQVSGLKR				Mascot
2042.0441	2042.0448	0.0007	0	1888	1906	TKVYGDADPSLTYQVSGLK				Mascot
2181.2278	2181.2253	-0.0025	-1	504	525	GLELIATGPAGAVDISAPILFR				Mascot
2230.0623	2230.0605	-0.0018	-1	684	704	VSGSASFPTNDSSDYLVTNLR				Mascot

Table S2. Strains and plasmids used in this study

Bacterial Strains and plasmids	Description	Reference
E. coli		
TOP10F'	F'[*lacI*^{q} Tn10(tet^{R})] *mcrA* Δ(*mrr*-*hsd*RMS-*mcr*BC) Φ80*lac*ZΔM15 Δ*lac*X74 *deo*R *nup*G *rec*A1 *ara*D139 Δ(*ara-leu*)7697 *gal*U *gal*K *rps*L(Str^{R}) *end*A1 λ^{-}	Invitrogen
DH5α	F$^{–}$ Φ80*lac*ZΔM15 Δ(*lac*ZYA-*arg*F) U169 *rec*A1 *end*A1 *hsd*R17 (r$_{k}^{–}$, m$_{k}^{+}$) *pho*A *sup*E44 *thi*-1 *gyr*A96 *rel*A1 λ$^{–}$	Invitrogen
CC118 (λpir)	Δ(*ara-leu*) *ara*D Δ*lac*X74 *gal*E *gal*K *pho*A20 *thi*-1 *rps*E *rpo*B *arg*E (Am) *rec*A1 Rf^{R} (λpir)	(17)
SM10	*thi*-1, *thr*, *leu*, *ton*A, *lac*Y, *sup*E, *rec*A::RP4-2-Tc::Mu; Km^{R}	Laboratory collection
MC4100	F^{-}Δ*lacU169 araD139 rpsL150 relA1 ptsF rbs flbB5301*	(18)
B1LK0	MC4100 Δ*tatC*	(19)
MCDSSAC	as MC4100, *amiA*Δ2–33, *amiC*Δ2–32	(20)
P. aeruginosa		
PAO1	Wild type	Laboratory collection
PA14	Wild type	Laboratory collection
PAOΔ*tat*	*tatABC* deletion mutant	(21)
PA14Δ*tat*	*tatABC* deletion mutant	This work
PA14Δ*tat attB*::*tat*	As PA14Δ*tat* but with *tatABC* under control of their own promoter integrated at the *attB* site; Tc^{R}	This work
PAOΔ*tat*Δ*lapA*	*tatABC* and *lapA* deletion mutant	This work
PA14Δ*xcpT*	*xcpT* deletion mutant	This work
PA14-3910H	chromosomally encoded PA3910$_{His6}$ translational fusion in PA14	This work
PA14Δ*tat*-3910H	chromosomally encoded PA3910$_{His6}$ translational fusion in PA14Δ*tat*	This work
PA14Δ*tat attB*::*tat*-3910H	chromosomally encoded PA3910$_{His6}$ translational fusion in PA14Δ*tat attB*::*tat;* Tc^{R}	This work
PA14Δ*xcpT*-3910H	chromosomally encoded PA3910$_{His6}$ translational fusion in PA14Δ*xcpT*	This work
PA14-4140H	chromosomally encoded PA4140$_{His6}$ translational fusion in PA14	This work
PA14Δ*tat*-4140H	chromosomally encoded PA4140$_{His6}$ translational fusion in PA14Δ*tat*	This work
PA14Δ*tat attB*::*tat*-4140H	chromosomally encoded PA4140$_{His6}$ translational fusion in PA14Δ*tat attB*::*tat;* Tc^{R}	This work
PA14Δ*xcpT*-4140H	chromosomally encoded PA4140$_{His6}$ translational fusion in PA14Δ*xcpT*	This work
PA14-2699H	chromosomally encoded PA2699$_{His6}$ translational fusion in PA14	This work
PA14Δ*tat*-2699H	chromosomally encoded PA2699$_{His6}$ translational fusion in PA14Δ*tat*	This work
**Plasmids**		
pKNG101	Sm^{R}, *oriR6K, oriTRK2, mobRK2, sacBR*$^{+}$	(17)

	(suicide vector)	
pKNGΔ*tatABC*	Suicide vector for *tatABC* deletion	(21)
Mini-CTX1	Contains *attP* site for integration at the *attB* site of *P. aerugionosa* chromosome; Tc^R	(22)
Mini-CTX1-*tatABC*	*tatABC* under control of their own promoter cloned in EcoRI of Mini-CTX1; Tc^R	This work
pCR2.1	TA cloning, *lacZ*α, ColE1, f1 ori, Ap^R Km^R	Invitrogen
pMMB67EH	*P. aeruginosa* expression vector with an IPTG inducible *tac* promoter; Ap^R (Cb^R)	(23)
pFLP2	Source of inducible *Flp* recombinase; Ap^R (Cb^R)	(24)
pKNGΔ*lapA*	Suicide vector for *lapA* deletion	This work
pRK2013	Km^R, ColE1, Tra+ Mob+	(25)
pKNGDT-11	Suicide vector for *xcpT* deletion; Sm^R	(26)
pCR2.1-3910H	$pa3910_{His6}$ in pCR2.1	This work
pCR2.1-4140H	$pa4140_{His6}$ in pCR2.1	This work
pKNG3910H	Suicide vector for $pa3910_{His6}$ insertion	This work
pKNG4140H	Suicide vector for $pa4140_{His6}$ insertion	This work
pCR2.1-2699H	$pa2699_{His6}$ in pCR2.1	This work
pKNG2699H	Suicide vector for $pa2699_{His6}$ insertion	This work
pT7-5	T_7 promoter preceding multiple cloning site; Ap^R	(27)
p*xcpT*	pMMB190, carrying *xcpT* gene under the P_{tac} promoter	(28)
pJN105	*P. aeruginosa* expression vector with an arabinose inducible P_{BAD} promoter; Gm^R	(29)
pJN2377H	*pa2377* and 150bp promoter region cloned into EcoRI of pJN105	This work
pJN2377H-M_1I	pJN2377H with a ATG>ATA substitution in *pa2377* codon 1 (M_1I)	This work
pJN2377H-M_{24}I	pJN2377H with a ATG>ATA substitution in *pa2377* codon 24 (M_{24}I)	This work
pJN2699H	*pa2699* and 279bp promoter region cloned into EcoRI of pJN105; Gm^R	This work
pJN2699H-M_1I	pJN2699H with a ATG>ATA substitution in *pa2699* codon 1 (M_1I)	This work
pJN2699H-M_{-47}I	pJN2699H with a ATG>ATA substitution in *pa2699* codon -47 (M_{-47}I)	This work
pUNI-PROM	pT7.5-derived vector allowing constitutive expression under the control of the *E. coli tat* promoter or inducible expression from the upstream T7 promoter (Ap^R)	(30)
pssAmiA-AmiAH	ssAmiA fused to mature AmiA carrying a C-terminal hexa-histidine tag in pUNI-PROM	Ize & Palmer, unpublished
pss2699-AmiAH	As pssAmiA-AmiAH but where AmiA signal peptide has been replaced by PA2699 signal peptide (M_{-47} to A_{-13})	This work
pss2699KK-AmiAH	As pss2699-AmiAH but where $R_{-38}R_{-37}$ have been substituded in $K_{-38}K_{-37}$	This work

Table S3. Primer sequences used in this study

Primer name	Primer Sequence
***tatABC* complementation**	
Promtat	5’-GTCGAGCAGGTCGGCGGGATC-3’
Endtat	5’-CCTCCGGCCACCCGCCAGTGA-3’
**qRT-PCR**	
2699RTFor	5’- CAACGCGATGATTCTCTACG-3’
2699RTRev	5’- TGAACAGGTTGTAGGCGATG -3’
16SRNAup	5’-CAGCTCGTGTCGTGAGATGT-3’
16SRNAdown	5’-GATCCGGACTACGATCGGTT-3’
2377RTFor2	5’-CGGTGGACCTCTCCAAGTTC-3’
2377RTRev2	5’-TTGTAGGCCGTGACATAGGC-3’
PA0688RTup	5’- CAACTTCAGCTATGCCGTGA -3’
PA0688RTdown	5’- GACCGTAGGTGCCGTTGTAG -3’
3910RTup	5’-AGCTGACCGATGGCGTGCAA-3’
3910RTdown2	5’-GCACCCGGTAGAAGATCGAC-3’
4140RTup	5’-GCACCAACGAAGAGGTTCTC-3’
4140RTdown	5’-GTCAGGTAACGGCTGGTTTC-3’
ToxARTFor2	5’-ATGCCACCTTCTTCGTCAGG-3’
ToxARTRev2	5’-TTCCCAGGTATCGTCGAGGT-3’
**Δ*lapA* deletion strain**	
ΔlapA -500 (1)	5’-AAGCCCTTCCCGTGCTGGGCGATTAAC-3’
ΔlapA -500 (2)	5’-GATTCGCCTAGGCGAAGCGC-3’
ΔlapA +500 (1)	5’-ACGATGAAAGGAGATTCTTC-3’
ΔlapA +500 (2)	5’-AAGGCATCGCAGAGCTGGGCACTGGTCA-3’
**PA3910H strain derivative strains**	
3910up2	5’-CGCAGTGGAAGGTGATCG-3’
3910hisdown	5’-TCAGTGATGGTGATGGTGATGGGCGCCGTCGGGCTGCAGT-3’
3910down2	5’-GGTCGAGGGTCTGCACAT-3’
3910hisup	5’-CATCACCATCACCATCACTGAGCCGCGGCTACCCCATTCC-3’
**PA4140H derivative strains**	
4140up2	5’-CGCGGTGCTGACCCGGCG-3’
4140hisdown2	5’-TCAGTGATGGTGATGGTGATGTGGCATCAGCCGGTCGAGCA-3’
PA4140down2	5’-CGCCCATTGCGGTTGGCACG-3’
4140hisup2	5’-CATCACCATCACCATCACTGAAATGCCGCTATGCGAGGC-3’
**pJN2377H and derivatives**	
2377SDup	5’-AAGTCTGCCGCGATTTTCC-3’
2377hisdown	TCAGTGATGGTGATGGTGATGGTAGATCGGCAGGTCCGGT-3’
2377M_1IFor	5’-CAGTCCCATCCATAAGGGGCGCGCCTG-3’
2377M_1IRev	5’-CAGGCGCGCCCCTTATGGATGGGACTG-3’
2377M_{24}IFor	5’-CAGAGGAACGGCACATAATCGACAGGGTGAAG-3’
2377M_{24}IRev	5’-CTTCACCCTGTCGATTATGTGCCGTTCCTCTG-3’

Table S3. (continued)

Primer name	Primer Sequence
**pJN2699H and derivatives**	
2699SDup	5’-TTGCGCTTGCAGGAGGATGCGTCA-3’
2699hisdown	5’TCAGTGATGGTGATGGTGATGGAAAGCGAAGCAGGAACAG-3’
2699M$_{-47}$IFor	5’-GGAGCACGCCATAAGCCACGATCCGC-3’
2699M$_{-47}$1IRev	5’-GCGGATCGTGGCTTATGGCGTGCTCC-3’
2699M$_{1}$IFor	5’-CCAGGAGGTTCCATAACCGCCGACCTGATC-3’
2699M$_{1}$IRev	5’-GATCAGGTCGGCGGTTATGGAACCTCCTGG-3’
**PA2699H derivative strains**	
2699up	5’-TGGACCTCGCTGTACTGG-3’
2699hisdown	5’-TCAGTGATGGTGATGGTGATGGAAAGCGAAGCAGGAACAG-3’
2699down	5’-GGTCTCGACGGTCATCTCG-3’
2699hisup	5’-CATCACCATCACCATCACTGACGTACCCGGGCGGGCCCT-3’
**pssAmiA-AmiAH and derivatives**	
AmiAEcFor	5’-TCTAGAAAAGACGAACTTTTA-3’
T7.5Rev2	5’-TGATTTAATTCTCATGTTTGA-3’
amiAR	5’-GCGCAAGCTTTTAGTGATGGTGATGGTGATGTCGCTTTTTCGAATGTGC-3’
T7	5’-GTAATACGACTCACTATAGGGC-3’
2699SSFor	5’-CGGGATCCATGAGCCACGATCCGCCGA-3’
2699SSRev	5’-TAAAAGTTCGTCTTTTCTAGAGGCGTTGGCGAAGGGCAG-3’
2699KKFor	5’-CGATCCGCCGAGCAAGGACAAGAAGCATTTCCTCACTACCAGTTC-3’
2699KKRev	5’-GAACTGGTAGTGAGGAAATGCTTCTTGTCCTTGCTCGGCGGATCG-3’
